# Packing-accelerated consecutive NIR excitation (PACE) enables high energy photocatalysis and antitumor immunity in vivo

**DOI:** 10.1126/sciadv.aeg3842

**Published:** 2026-10-07

**Authors:** Yishen Liu, Shuo Wang, Wenbo Hu, Haotian Guo, Zafar Mahmood, Yuqin Liao, Yanna Pan, Xiaofen Wang, Wumei Wang, Zhiyun Zhang, Mingxuan Jia, Haolin Zhang, Xiaodong Zeng, Huaiyi Huang, Xinyuan Fan, Xuechuan Hong, Zhijun Sun, Yuling Xiao

**Affiliations:** ^1^ State Key Laboratory of Drug Research & Center of Pharmaceutics, Shanghai Institute of Materia Medica, Chinese Academy of Sciences, Shanghai, 201203, China.; ^2^ State Key Laboratory of Oral & Maxillofacial Reconstruction and Regeneration, Key Laboratory of Oral Biomedicine Ministry of Education, Hubei Key Laboratory of Stomatology, School & Hospital of Stomatology, Frontier Science Center for Immunology and Metabolism, School of Pharmaceutical Sciences, Wuhan University, Wuhan, 430079, China.; ^3^ Shandong Laboratory of Yantai Drug Discovery, Bohai Rim Advanced Research Institute for Drug Discovery Yantai, 264117, China.; ^4^ State Key Laboratory of Flexible Electronics (LoFE), and Xi’an Institute of Flexible Electronics (IFE), Northwestern Polytechnical University, Xi’an, 710072, China.; ^5^ College of Chemistry and Molecular Engineering, Peking University, Beijing, China.; ^6^ School of Chemical Engineering and Light Industry, Guangdong University of Technology, Guangzhou 510006, China.; ^7^ School of Pharmaceutical Sciences (Shenzhen), Shenzhen Campus of Sun Yat-sen University, Shenzhen, Guangdong 518107, P.R. China.; ^8^ School of Pharmaceutical Sciences, Key Laboratory of Smart Drug Delivery, Ministry of Education, National Key Laboratory of Advanced Drug Formulations for Overcoming Delivery Barriers, Fudan University, Shanghai 201203, China.

## Abstract

Near-infrared (NIR) photocatalysis promises deep-tissue compatibilities but remains fundamentally constrained by two bottlenecks: insufficient photon energy and rapid charge recombination. Here, we report packing-accelerated consecutive excitation (PACE), a mechanism enabling low energy near-infrared (NIR) photons to drive high energy single electron transfer (SET) reactions. We design a self-assembling ruthenium complex, **Ru-3**, that forms densely packed nanoaggregates exhibiting aggregation-stabilized excited states, a pronounced excited state absorption band at ∼1315 nm, and nanosecond scale triplet lifetimes. Under dual NIR-I/IIa irradiation (808 nm/1310 nm), **Ru-3** nanoparticles exhibit photophysical behavior consistent with consecutive photon harvesting and access to a higher-energy reactive state that supports efficient SET catalysis under aqueous conditions. This PACE-driven reactivity enables robust oxidation of mitochondrial NADH to NAD^+^, disrupting electron-transport homeostasis, inducing cGAS-STING dependent pyroptosis, and reprogramming tumor-associated macrophages toward an M1 phenotype. In multiple murine tumor models, PACE-mediated photocatalysis elicits strong antitumor immunity and leads to tumor regression with minimal systemic toxicity. This work establishes PACE as a new photochemical paradigm that overcomes the intrinsic energy and charge-separation limits of NIR photoredox chemistry, opening a route toward high-energy photocatalysis in complex biological environments.

## INTRODUCTION

Photocatalysis harnesses photon energy to drive thermodynamically demanding transformations, enabling mechanistically diverse reactions in chemical synthesis and biological systems (*1*, *2*). Applications ranging from endogenous-substrate conversion in photodynamic therapy (*3*) to prodrug activation (*4*, *5*) and photocatalytic proximity labeling (*6*, *7*) have demonstrated the unique spatiotemporal precision attainable in living environments. Nevertheless, high-energy photocatalysis in deep tissue remains constrained by the fundamental trade-off between photon wavelength and photochemical driving force. UV and visible light (100–700 nm) (*8*) deliver sufficient energy to support single electron transfer (SET) (*9*–*12*) and energy transfer (EnT) (*13*–*15*) pathways, but their poor tissue penetration, nonspecific absorption by biomacromolecules, and inherent phototoxicity severely limit in vivo applicability (*16*–*20*) (Fig. 1A). In contrast, near-infrared light (NIR, λ > 700 nm) penetrates tissue more effectively and minimizes off-target interactions (*21*, *22*), yet its intrinsically low photon energy (∼35 kcal mol^−1^) (*23*, *24*) remains a barrier to efficient photocatalysis.

**Fig. 1.**
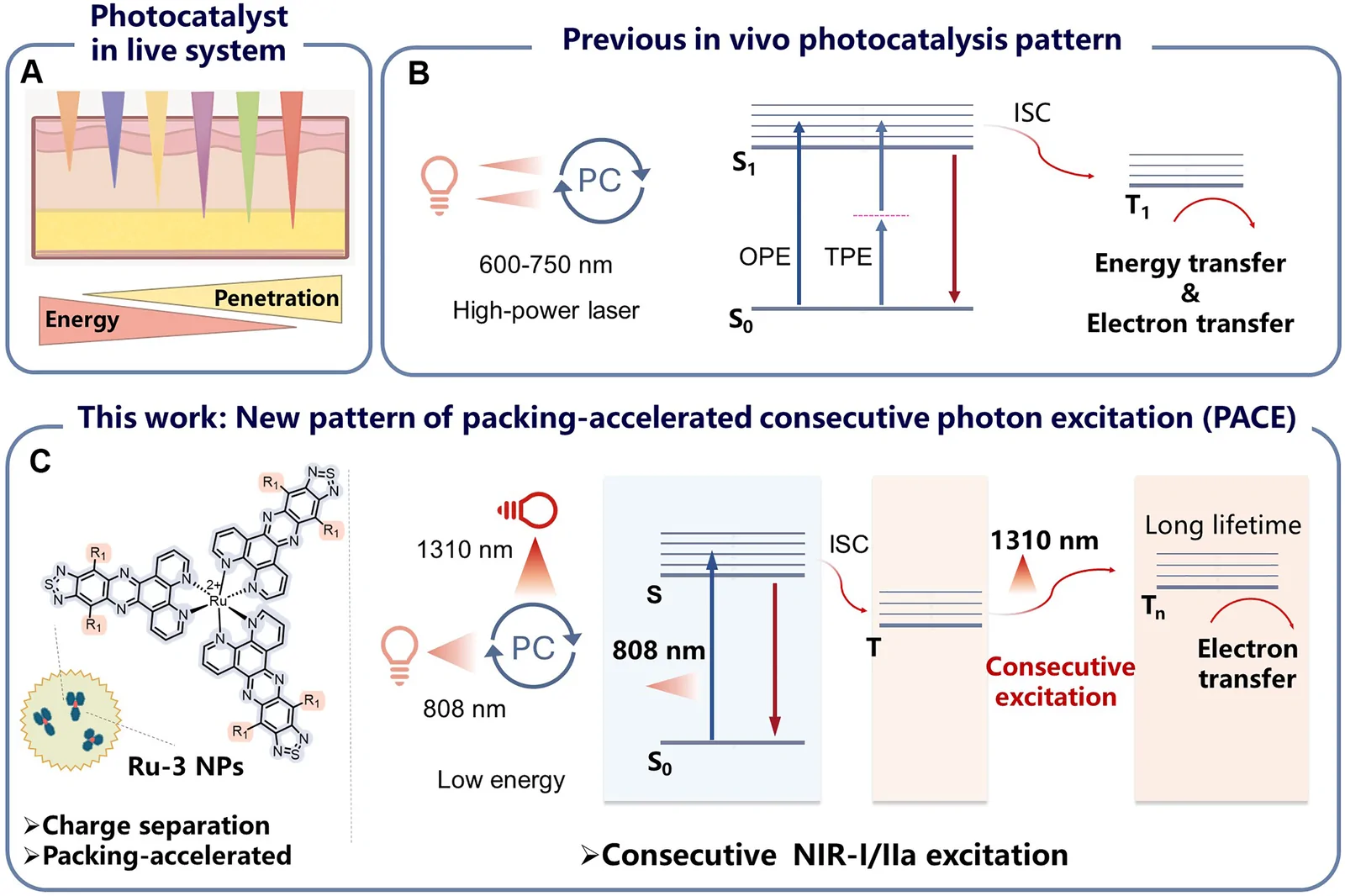
Reported in vivo photocatalytic pattern and the PACE mechanism in this work. (**A**) Schematic illustrating the trade-off between photon wavelength (penetration depth increases with longer wavelengths) and energy (decreases with longer wavelengths). Created in BioRender. lou, Z. (2026) https://BioRender.com/1kngn91 (**B**) Scheme of reported one-photon excitation (OPE) and two-photon excitation (TPE) photocatalytic mechanisms. (**C**) Scheme of the packing-accelerated consecutive photon excitation (PACE) mechanism, where under dual NIR-I/IIa (808 nm/1310 nm) excitation, **Ru-3** nanoparticles undergo the PACE process through consecutive photon absorption, transitioning to higher-energy reactive states to achieve efficient single electron transfer catalysis.

To bridge this energy gap, sophisticated multi-photon strategies have emerged. Two-photon excitation (TPE), the most widely adopted in vivo approach, simultaneously absorbs two low energy photons to achieve high energy excited states (*25*–*27*) (Fig. 1B). However, TPE processes require high-intensity pulsed lasers and predominantly operate through energy transfer mechanisms, which limit reaction scope compared to electron transfer processes essential for applications like photodynamic therapy (*25*–*27*). Alternative approaches including triplet–triplet annihilation upconversion (TTA-UC) (*28*–*30*) and consecutive photoinduced electron transfer (conPET) (*27*, *31*, *32*) achieve excitation by sequentially photon pooling. However, these methods demand precise energy-level matching and nanoscale assembly of sensitizer–catalyst pairs (*29*), or rely on oxygen-sensitive intermediates (*27*–*31*), restricting applications to highly controlled or deoxygenated conditions. Overall, no existing NIR strategy (beyond 900 nm light) simultaneously achieves deep-tissue compatibility, oxygen tolerance, and SET-level photochemical driving force.

Here, we report packing-accelerated consecutive excitation (PACE), a previously unrecognized NIR photocatalytic mechanism that enables sequential harvesting of NIR-I/IIa photons (700–900/1300–1400 nm) to generate high-energy reactive states in vivo (Fig. 1C). Central to this strategy is a self-assembling ruthenium complex, **Ru(H7)_3_(PF_6_)_2_** (**Ru-3**), which upon nanoparticle formation (**Ru-3 NPs**) exhibits stabilized excited-state absorption at ∼1315 nm and prolonged charge separation independent of the Ru center (*33*, *34*). **Ru-3 NPs** are initially excited by 808 nm light, followed by further excitation to a higher energy state via 1310 nm light. This excited state exhibits an extended lifetime, enabling electron transfer processes that drive the catalytic reaction. Through this PACE-enabled SET chemistry, we achieve robust oxidation of mitochondrial NADH to NAD^+^, thereby inducing gasdermin-mediated pyroptosis and reprogramming macrophages toward an antitumor phenotype. These findings establish PACE as a distinct photochemical paradigm that overcomes longstanding energetic barriers in NIR photocatalysis and enables deep-tissue catalytic manipulation.

## RESULTS

### Photophysical foundation of PACE: Aggregation-stabilized charge-separated states in Ru-3

To develop an effective NIR photocatalyst, several key requirements must be met: (i) low excitation energy, (ii) efficient charge separation, and (iii) prolonged excited-state lifetimes for sustained catalytic activity. Our previous work has shown that a donor-acceptor-donor (D-A-D) molecule **H7** exhibits effective intramolecular charge separation characteristics (*33*, *34*) (Fig. 2A). To further improve the charge separation efficiency, we designed and synthesized a ruthenium complex **Ru-3** by condensing 4,7-bis(4-(diphenylamino)phenyl) benzo[c][1,2,5]thiadiazole-5,6-diamine **3** with Ru(Phen)_3_(PF_6_)_2_ (Fig. 2A, figs. S1 to S7) (*35*–*38*). TD-DFT showed a lower HOMO-LUMO energy gap of **Ru-3** compared to H7, indicating a redshift in its optical spectrum (Fig. 2B). The electron–hole overlap ratio (O_e/h_) decreased from 68.81% in H7 to 63.72% in **Ru-3**, reflecting a substantial enhancement in charge-separation efficiency (Fig. 2C). Interestingly, electron-hole analyses revealed that **Ru-3** does not exhibit a dominant conventional metal-to-ligand charge-transfer (MLCT) character, suggesting that the Ru core may promote charge separation through alternative mechanisms rather than a classical MLCT pathway (fig. S8A).

**Fig. 2.**
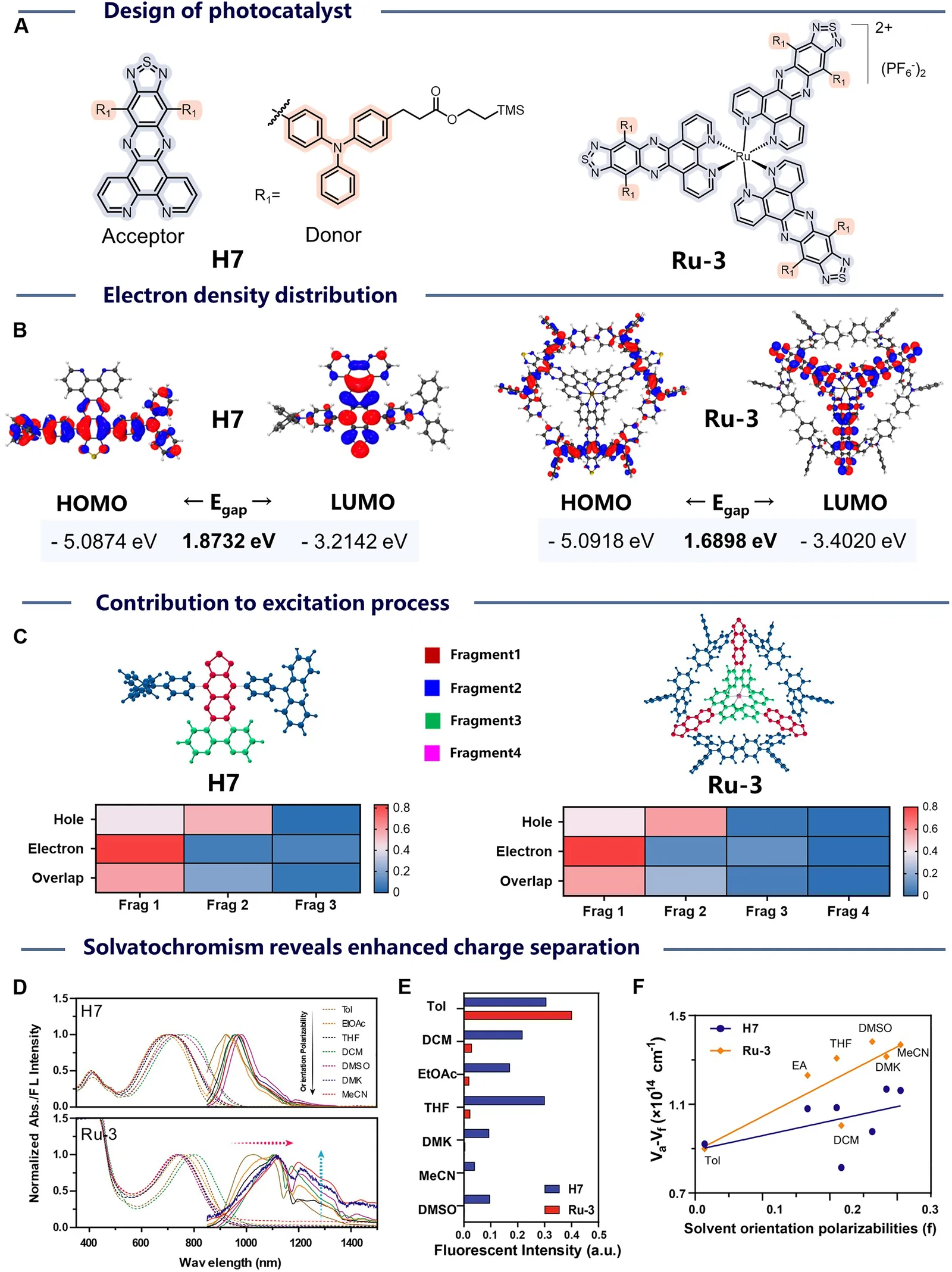
Design and photophysical properties of H7 and Ru-3. (**A**) Chemical structure of H7 and Ru-3. (**B**) Electron density distributions of the highest occupied molecular orbital (HOMO) and the lowest unoccupied molecular orbital (LUMO) for **H7** (left) and **Ru-3** (right). (**C**) The contribution of each fragment to the excitation process in the S_0_ (hole) → S_1_ (electron) transition. The pink fragment represents the Ru element. (**D** and **E**) Absorption (dotted line) and emission (solid line) spectra (D) (λ_ex_ = 800 nm) and fluorescent intensity (E) of **H7** and **Ru-3** in different solvents. Tol, toluene; EtOAc, ethyl acetate; THF, tetrahydrofuran; DCM, dichloromethane; DMSO, dimethylsulfoxide; DMK, acetone; MeCN, acetonitrile. (**F**) Lippert-Mataga plot of Stokes shift (v_a_ − v_f_) for **H7** and **Ru-3** versus solvent orientation polarizability; v_a_ and v_f_ represent the absorption and emission frequency, respectively.

Spectroscopic analysis showed that **Ru-3** exhibits strong solvatochromic behavior, with its emission being strongly quenched in polar solvents (Fig. 2E). Notably, **Ru-3** displayed a dual-emission profile with a secondary peak at ∼1200 nm that grows with increasing solvent polarity (Fig. 2D), indicating the presence of multiple excited states with varying degrees of charge separation, where the more polar states are stabilized in polar environments. Lippert–Mataga analysis indicated that the excited-state dipole moment of **Ru-3** is significantly larger than that of **H7** (Fig. 2F), further corroborating enhanced spatial charge separation. Photostability tests under 808 nm laser irradiation demonstrated **Ru-3**’s robustness (fig. S8, B and C), highlighting its potential as an efficient broadband photocatalyst.

### Mechanistic evolution of PACE: Aggregation-induced stabilization of long-lived triplet manifolds

Molecular aggregation significantly influences the photophysical properties and photocatalytic performance of chromophores, which has emerged as a promising strategy to stabilizing excited-state and enhancing catalytic activity. To elucidate how molecular packing influences photoactivity, we titrated **H7** and **Ru-3** solutions in DMSO into water to induce aggregation (*39*–*41*). Dynamic light scattering (DLS) analysis shows the formation of nanoaggregates when water content reached 10% (fig. S9). Increasing water fractions caused red-shifted absorption and emission bands, indicative of J-aggregates (Fig. 3A, fig. S10). Notably, **Ru-3**_**50**_ (DMSO/H_2_O = 50/50) exhibited minimal fluorescence but a remarkable 76.45-fold increase in reactive oxygen species (ROS) generation, outperforming **H7**, previously reported **Ru-1**, and potent NIR photosensitizer ICG by approximately 9.75-, 18.77-, and 5.27-fold, respectively (Fig. 3B, figs. S11 to S14, tables S1 to S3).

**Fig. 3.**
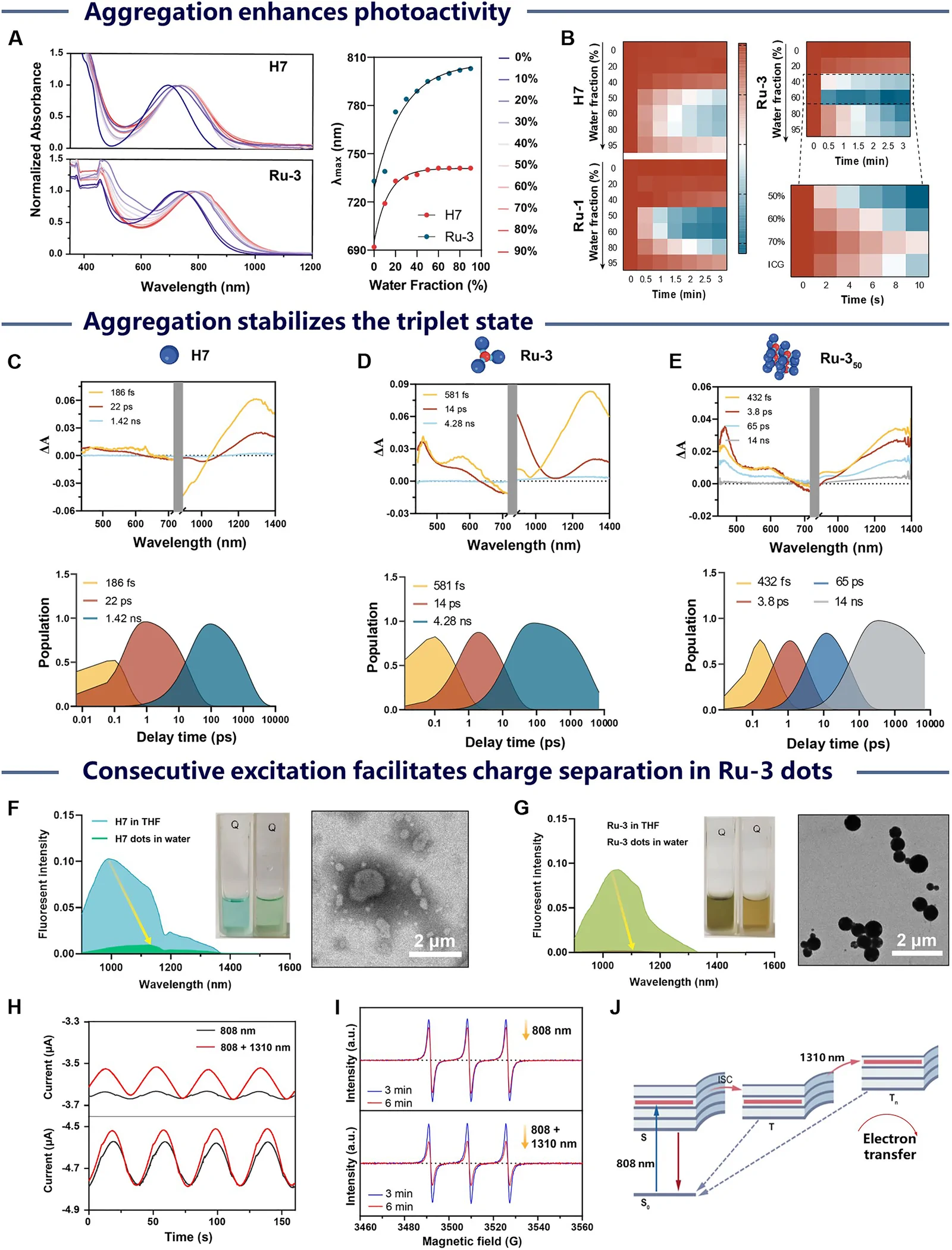
Photophysical evidence supporting the proposed PACE mechanism in Ru-3 aggregates. (**A**) Normalized absorption spectra (left) of **H7** and **Ru-3** in DMSO/H_2_O solutions with varying water fractions and corresponding shifts in maximum absorption wavelengths (right) as the water fraction increases. (**B**) Heatmap depicting the photodegradation efficiency of 1,3-diphenylisobenzofuran (DPBF) by **H7**, **Ru-1**, and **Ru-3** in DMSO/H_2_O solutions with varying water fractions, under 808 nm laser irradiation (power density: 0.5 W cm^−2^). Normalized absorption of DPBF at 415 nm is reported as mean ± s.d. (**C** to **E**) EADS derived from global analysis of fs-TA spectra for **H7** in DMSO (C), **Ru-3** in DMSO (D), and **Ru-3** in a DMSO/H_2_O (50:50) mixture (**Ru-3**_**50**_) (E), following photoexcitation with an 800 nm pulse (80 μW) at varying pump-probe delay times. (**F**) Fluorescence emission spectra of **H7** in THF and **H7** dots in water. Inset: Photographs of **H7** monomers and **H7** dots under ambient light. Right: Representative TEM image of **H7** dots (scale bar, 2 μm). (**G**) Fluorescence emission spectra of **Ru-3** in THF and **Ru-3** dots in water. Inset: Photographs of **Ru-3** monomers and **Ru-3** dots under ambient light. Right: Representative TEM image of **Ru-3** dots (scale bar, 2 μm). (**H**) Photocurrent responses of **H7** and **Ru-3** dots were measured using a photoelectrochemical workstation under either 808 nm laser irradiation or consecutive 808 nm and 1310 nm laser irradiation. (**I**) ESR spectra of TEMPO radicals were recorded after 6 minutes of irradiation with an 808 nm laser or consecutive irradiation with 808 nm and 1310 nm lasers. (**J**) Proposed mechanism of PACE. Aggregation of **Ru-3** stabilizes the intermediate excited state, enabling sequential absorption of 808 nm and 1310 nm photons. This promotes **Ru-3** to a higher-energy reactive triplet state, enhancing charge separation to drive a SET process.

To probe the excited-state dynamics underlying this enhanced activity, femtosecond transient absorption (fs-TA) spectroscopy was performed (fig. S15). Upon 800 nm excitation (∼100 fs pulses), both **H7** and **Ru-3** in DMSO displayed excited-state absorption (ESA) at ∼500 nm and ∼1315 nm, with ground-state bleach (GSB) spanning 600–800 nm (Fig. 3, C and D). In contrast, **Ru-3**_**50**_ revealed an additional evolution-associated difference spectrum (EADS) component absent in monomers, indicating a distinct excited-state species (Fig. 3, C and E). The initial EADS, associated with S_1_, evolved within 432 fs into a second EADS consistent with triplet-state (T_1_) formation. A third EADS emerged at 3.8 ps, persisting for 65 ps, while notably, **Ru-3**_**50**_ exhibited a long-lived EADS (∼14 ns) tied to T_1_ ESA (∼1315 nm) (Fig. 3E, fig. S16). This extended triplet lifetime suggested that the dense packing within **Ru-3**_**50**_ aggregates stabilizes triplet states, thereby increasing triplet population and significantly enhancing photosensitization efficiency.

To further quantify the aggregation effect, transient absorption spectroscopy was performed on **Ru-3** in DMSO/H_2_O mixtures with varying DMSO fractions. As the DMSO fraction decreased from 100% to 25%, resulting in progressively enhanced aggregation, the ESA intensity at ∼1315 nm increased from 1.67 to 2.61 mOD under identical excitation conditions (fig. S17). Consistently, kinetic traces monitored at 1315 nm exhibited slower decay dynamics and a more pronounced long-lived component in the more aggregated samples (fig. S18). These results indicate that molecular packing within the aggregates stabilizes the triplet-related excited state and extends its lifetime.

### Consecutive NIR excitation of aggregated Ru-3 drives access to a higher-energy triplet state for SET catalysis

The nanoaggregates of **H7** and **Ru-3** (**H7** and **Ru-3** dots) were synthesized via nanoprecipitation methods to investigate the charge separation dynamics and efficiency (*42*, *43*). Transmission electron microscopy (TEM) analysis demonstrated that **Ru-3** dots formed uniform nanospheres with highly ordered molecular packing, resulting in nearly complete emission quenching compared to **Ru-3** in its non-aggregated form (Fig. 3, F and G). Photocurrent response measurements further revealed that **Ru-3** dots generated a strong positive photocurrent, whereas **H7** dots produced only a faint response under 808 nm laser irradiation (Fig. 3H).

To further investigate the charge separation mechanism, electron spin resonance (ESR) spectroscopy, employing TEMPO as a spin trap, was conducted. The results indicated faster decay of the TEMPO signal under combined 808 nm and 1310 nm irradiation compared to 808 nm alone (Fig. 3I), indicating enhanced charge separation under dual-wavelength excitation. Additionally, ESR spectra of **Ru-3** dots under 808 nm irradiation revealed the presence of TEMPO and DMPO-OH signals, verifying the production of singlet oxygen (^1^O_2_) and hydroxyl radicals (HO•), respectively (fig. S19, A and B) (*37*). Notably, consecutive excitation with 808 nm and 1310 nm intensified the DMPO-OH signal while reducing the TEMPO signal. Given that ^1^O_2_ generation arises from T_1_ energy transfer to oxygen, we propose that 1310 nm excitation may promote the formation of more reactive excited states and facilitate charge-transfer processes (Fig. 3J, fig. S19C) and thereby contributing to the single electron transfer-based (SET) catalytic mechanism. These findings highlight PACE as a promising strategy to address the challenges of charge separation and low photon energy in NIR photoredox catalysis.

### PACE-enabled NADH photoredox catalysis in aqueous environments

Building on the exciting PACE system with enhanced SET catalytic ability, we aimed to apply PACE process into biological systems. Given that SET offers advantages over EnT in photodynamic therapy (PDT) due to its efficacy in hypoxic environments, we focused on oxidizing important biomolecules such as NADH, which could potentially be utilized for therapeutic applications. To this end, we conducted a self-assembly system using PEO_114_-b-PS_100_ which preserved **Ru-3** aggregates’ photocatalytic activity in aqueous media via a solvent-switch method (fig. S20, A and B). TEM confirmed effective encapsulation within polymersomes (fig. S20, C and D). Polymersome size decreased with increasing water/THF ratio to 1:1.175, yielding ∼150 nm **Ru-3 NPs** (fig. S20E). To evaluate their colloidal stability, **Ru-3 NPs** were incubated in PBS, 10% FBS medium, and serum, maintaining nanoscale diameters without noticeable aggregation over a period of 3 days (fig. S21). In addition, **Ru-3 NPs** exhibited negligible changes in particle size upon serial dilution (0–200×), demonstrating their robust physicochemical stability under physiological conditions (fig. S22).

NADH photocatalytic oxidation in aqueous media was assessed using ^1^H NMR and UV-Vis spectroscopy. Initial tests without **Ru-3 NPs** under 808 nm, 1310 nm, or both showed no detectable changes (figs. S23 and S24A). Similarly, **Ru-3 NPs** alone or with 1310 nm irradiation did not oxidize NADH in the dark (fig. S24B). However, under 808 nm or combined 808 + 1310 nm irradiation, **Ru-3 NPs** effectively oxidized NADH to NAD^+^, as indicated by a reduced absorbance peak at 339 nm and altered ^1^H NMR spectra (Fig. 4A, figs. S25 and S26). The addition of 1310 nm irradiation significantly enhanced oxidation, increasing the turnover number (TON) by ∼1.84-fold, with a temperature rise to 37.8°C (figs. S27 and S28A). In contrast, **H7 NPs** achieved only a ∼1.19-fold TON increase (fig. S28, B and C). The enhanced performance of **Ru-3 NPs** was attributed to their prolonged ∼1315 nm transient absorption lifetime and improved charge separation.

**Fig. 4.**
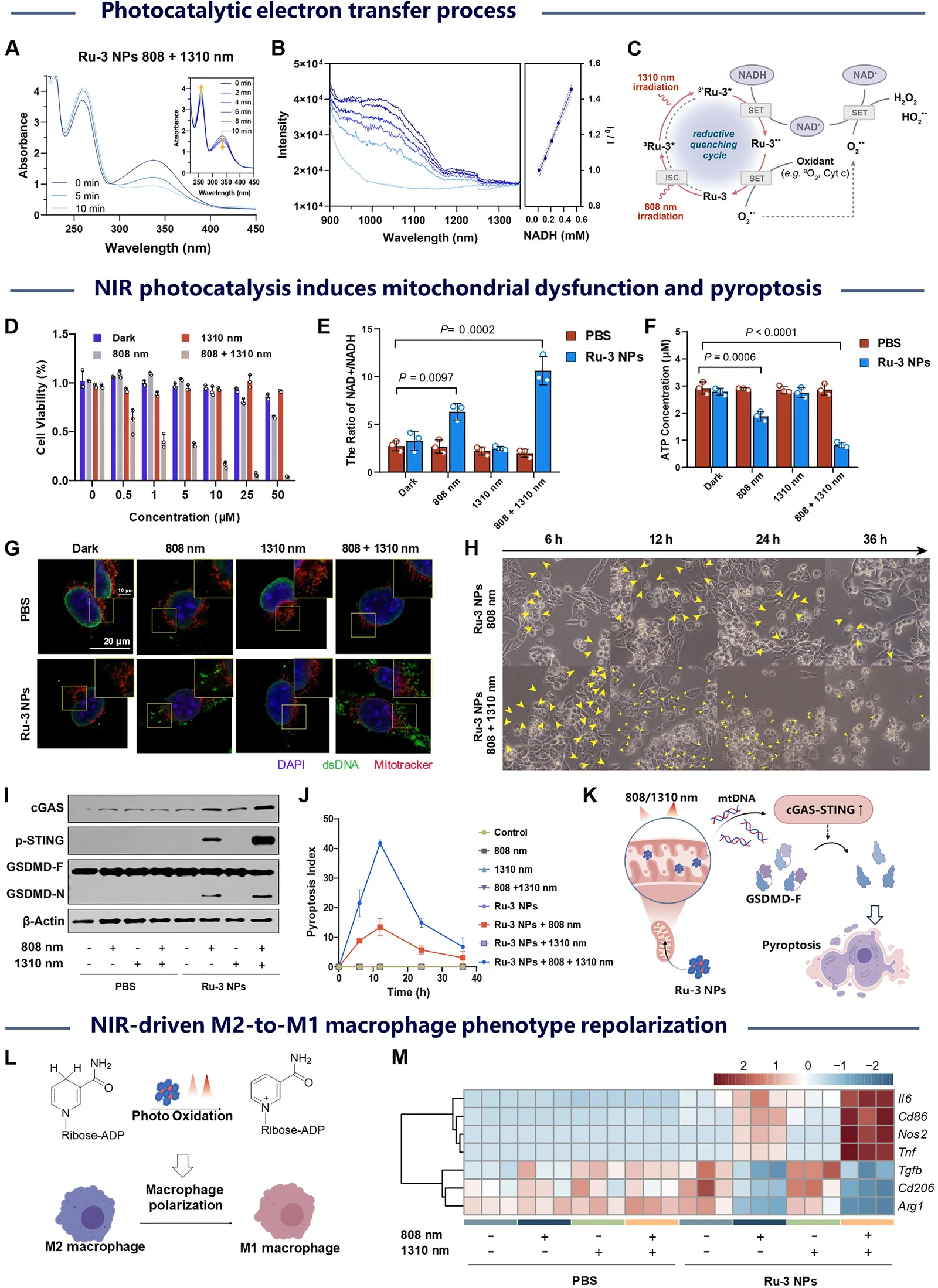
Induction of tumor cell pyroptosis and macrophage polarization by PACE. (**A**) UV-vis spectra shows the photo-oxidation of NADH (180 μM) under 808 (0.5 W cm^−2^) + 1310 nm irradiation (1.0 W cm^−2^) in the presence of **Ru-3 NPs** (10 μM). (**B**) Stern−Volmer plot depicting the interaction of NADH with **Ru-3 NPs** excited state. I_0_ and I represent emission intensities in the absence and presence of NADH. (**C**) Proposed reductive cycle illustrating the photocatalytic conversion of NADH to NAD^+^ through PACE. (**D**) Dark and photo cytotoxicity of **Ru-3 NPs** against 4 T1 cells (CCK-8 assay, *n* = 3). (**E** and **F**) NAD^+^/NADH ratio (E) and ATP concentration (F) in 4 T1 cells treated with **Ru-3 NPs** (10 μM) under different irradiation conditions (*n* = 3). (**G**) Confocal images of 4 T1 cells post-treatment show nucleus (DAPI, blue), dsDNA (anti-dsDNA antibody, green), and mitochondria (MitoTracker Green, red). (**H**) Representative microscopy images of 4 T1 cells at various time points post-treatment show pyroptotic cells (*n* = 3). (**I**) Immunoblot analysis of cGAS, p-STING, GSDMD-N, and GSDMD-F in 4 T1 cells treated with **Ru-3 NPs** (10 μM) under different conditions (*n* = 3). (**J**) Pyroptosis index of 4 T1 cells assessed at 6, 12, 24, and 36 hours after treatments. (**K**) Proposed signal transduction activating pyroptosis after NADH photo-oxidation. Created with BioGDP.com (agreement number: GDP2026N3TE0F) (**L**) Proposed macrophage polarization regulation after NADH photo-oxidation. Created with BioGDP.com (agreement number: GDP2026N3TE0F) (**M**) Heatmap illustrating the mRNA level of M1 (top) and M2 macrophage markers (bottom) after **Ru-3 NPs** (10 μM) treatment under different conditions (808 nm, 0.5 W cm^−2^; 1310 nm, 1.0 W cm^−2^; *n* = 3). Normalized mRNA level is reported as mean ± s.d. For ANOVA tests with Dunnett’s multiple comparisons test, *P < 0.05; **P < 0.01; ***P < 0.001; ****P < 0.0001; ns, not significant.

To further assess the catalytic kinetics and substrate dependence, complementary studies were conducted using **Ru-3**_**50**_ aggregates under dual-wavelength (808 + 1310 nm) irradiation. Time-dependent UV–vis measurements performed at different initial NADH concentrations (90–270 μM) revealed similar apparent consumption rates (7.55–8.31 μM min^−1^) and comparable TON values (20.9–24.2), indicating a saturation-like catalytic behavior within the investigated concentration range (fig. S29). Notably, the reaction rates remained essentially constant throughout over 20–30 min of continuous irradiation, demonstrating sustained catalytic activity and good cycle stability of the **Ru-3** based aggregated systems under dual-wavelength irradiation.

Subsequently, a Stern-Volmer titration was conducted to elucidate the photocatalytic NADH oxidation mechanism. Increasing NADH to 0.6 mM reduced **Ru-3 NP** fluorescence, yielding a quenching constant of 0.92 mM^−1^ (Fig. 4B). ESR spectroscopy with 5-(2,2-dimethyl-1,3-propoxycyclo-phosphoryl)-5-methyl-1-pyrroline-*N*-oxide (CYPMPO) as a radical trap (fig. S30A) confirmed NAD• radical formation, suggesting a SET process between excited Ru-3 aggregates and NADH (Fig. 4C). To further support the thermodynamic feasibility of NADH oxidation, the excited-state reduction potential of **Ru-3** was estimated to be +1.21 V vs NHE based on electrochemical and spectroscopic analysis (fig. S31), which is more positive than the oxidation potential of NADH (+0.93 V vs NHE), suggesting that photoexcited **Ru-3** is thermodynamically capable of oxidizing NADH. Control experiments further showed negligible NADH oxidation under elevated temperature conditions (fig. S32), while the reaction persisted in the presence of NaN_3_ (fig. S33), supporting a SET-dominated mechanism rather than a purely ^1^O_2_-mediated process.

Given the hypoxic nature of biological systems, particularly in the tumor microenvironment, we further tested O_2_-independent NADH oxidation using cytochrome c (cyt c), an electron transport chain carrier, as an alternative electron acceptor. Under deoxygenated conditions, NADH oxidation was largely suppressed, indicating that electron transfer requires an external electron acceptor in the absence of O_2_ (fig. S34). Notably, when oxidized cyt c was added to a solution containing **Ru-3 NPs** and NADH, irradiation at 808 nm generated distinct absorption bands at 520 and 550 nm, characteristic of reduced Fe^2+^-cyt c (*44*) (fig. S30B). The subsequent application of 1310 nm irradiation further promoted cyt c reduction (fig. S30C). These results suggest that cyt c can function as an alternative electron acceptor under hypoxic conditions, supporting NADH oxidation via an oxygen-independent electron-transfer process.

### PACE-driven mitochondrial redox collapse induces pyroptosis and reprograms macrophage polarization

Given the NADH oxidation capability of **Ru-3 NPs** in vitro, we then evaluate its photocatalytic activity in cells. ICP-MS revealed significant accumulation of **Ru-3** in mitochondria via electrostatic interactions (table S4). No dark toxicity was observed for **Ru-3 NPs** up to 50 μM. Photocytotoxicity was moderate under 808 nm irradiation alone but significantly enhanced with combined 808 nm and 1310 nm irradiation (Fig. 4D).

Treatment with **Ru-3 NPs** alone or with irradiation induced minimal changes in NAD^+^/NADH ratios, indicating that neither **Ru-3 NPs** nor irradiation alone drives NADH oxidation. However, combining **Ru-3 NPs** with 808 nm or dual 808/1310 nm irradiation elevated the NAD^+^/NADH ratio by ∼2.31- and 3.88-fold, respectively, compared to the light-shielded group (Fig. 4E). This shift disrupted NAD^+^/NADH homeostasis, leading to lowered ATP levels and elevated mitochondrial reactive oxygen species (mtROS), consistent with impaired mitochondrial electron transport chain function (Fig. 4F and fig. S35A). Consequently, mitochondrial DNA (mtDNA) was released (Fig. 4G), activating the cGAS-STING pathway and triggering gasdermin D (GSDMD) cleavage (Fig. 4I and fig. S35B). Optical microscopy revealed characteristic cellular swelling and ballooning, along with elevated LDH and ATP release (Fig. 4H, fig. S35, C and D), indicative of GSDMD-mediated pyroptosis (Fig. 4K, fig. S36). In 4 T1 cells treated with Ru-3 NPs under dual 808 and 1310 nm irradiation, the pyroptosis index increased steadily over 12 hours, reaching 41.76%, a ∼3.1-fold increase relative to 808 nm irradiation alone (*45*) (Fig. 4J). In 4MOSC1 cells, the peak index reached ∼32.26% (fig. S35E). NAD^+^ serves as a key cofactor in α-ketoglutarate (αKG) and acetyl-CoA biosynthesis, both vital for epigenetic and transcriptional control of inflammation (*46*) (Fig. 4L). NIR photocatalysis was further evaluated in RAW264.7-derived M2 macrophages. Treatment with **Ru-3 NPs** under NIR irradiation significantly increased the expression of *Il6*, *Tnf*, *Cd86*, and *Nos2*, while reducing *Tgfb* and *Cd206* levels, consistent with M2-to-M1 macrophage repolarization (Fig. 4M, fig. S37). Together with its ability to induce pyroptosis, these results establish NIR-I/IIa photocatalysis as a versatile platform for both tumor cell killing and immune microenvironment reprogramming, offering promising opportunities for cancer therapy.

### PACE-modulated photocatalytic therapy enhances antitumor efficacy in vivo

The therapeutic efficacy of PACE-modulated photocatalytic therapy was investigated in murine models of subcutaneous breast carcinoma and orthotopic head and neck squamous cell carcinoma (HNSCC). We first evaluated the in vivo safety and pharmacokinetic properties of **Ru-3 NPs**. Treated mice showed stable body weight, no detectable organ dysfunction or inflammatory responses, and a prolonged blood half-life of 44.88 h, indicating favorable biocompatibility (fig. S38). ICP–MS analysis further revealed that Ru was primarily eliminated through fecal excretion within 24–72 hours after administration, whereas urinary excretion was negligible, consistent with hepatobiliary/gastrointestinal clearance (fig. S39). At day 50, only minimal residual Ru was detected in major organs, with the highest level found in the kidney (∼0.109% of the injected dose) (fig. S40). Moreover, serum biochemical and hematological indices remained within normal ranges (figs. S41 and S42). Together, these results demonstrate the efficient clearance and favorable long-term biosafety of **Ru-3 NPs** in vivo.

We then validated the tissue penetration capability of dual-NIR irradiation. Using 1% intralipid layers of 0–6 mm to mimic tissue, NADH photo-oxidation was still measurable through 6 mm, though the reaction extent decreased with increasing thickness (fig. S43). This confirms that dual-NIR PACE photocatalysis retains activity under moderate tissue-like optical scattering. Supporting its potential for in vivo applications. Subcutaneous 4 T1 tumors were established by implanting 4 T1 cells into the right flanks of Balb/c mice, while orthotopic 4MOSC1 tumors were generated by injecting 4MOSC1 cells into the dorsum linguae of C57BL/6 mice, with an 8-day period allowed for tumor development (Fig. 5A). Mice were randomized into eight groups receiving either phosphate-buffered saline (PBS) or **Ru-3 NPs** under dark conditions, 808 nm irradiation, 1310 nm irradiation, or combined 808 nm and 1310 nm irradiation. Minimal tumor growth inhibition was observed in both 4 T1- and 4MOSC1-bearing mice treated with **Ru-3 NPs** alone, 808 nm irradiation alone, 1310 nm irradiation alone, or their combination (Fig. 5B), consistent with cellular photocytotoxicity results. In comparison, **Ru-3 NPs** combined with 808 nm irradiation modestly reduced tumor growth in 4 T1 models (Fig. 5B). Notably, PACE-modulated photocatalytic therapy utilizing NIR-I/IIa light produced significantly enhanced antitumor effects, achieving near-complete tumor regression in 40% of 4 T1-bearing mice and demonstrating similar efficacy in the HNSCC model (Fig. 5B and figs. S44 and S45). Given the involvement of pyroptosis and M1 macrophage polarization in antitumor immunity, immune cell profiles were analyzed at the study endpoint. Flow cytometry of tumor-draining lymph nodes (tdLNs) (fig. S46) showed that the PACE strategy significantly increased CD8^+^ memory T cells (49.88% vs. 16.29%) and dendritic cells (22.52% vs. 8.88%) while reducing regulatory T cells (6.90% vs. 20.76%) compared to the dark control group, thereby mitigating immunosuppression and immune evasion (Fig. 5, C and D). Immunohistochemistry further confirmed increased CD8^+^ T cell infiltration into 4 T1 and 4MOSC1 tumors treated with **Ru-3 NPs** combined with 808 nm or dual-wavelength irradiation, reflecting enhanced antitumor immunity (fig. S47).

**Fig. 5.**
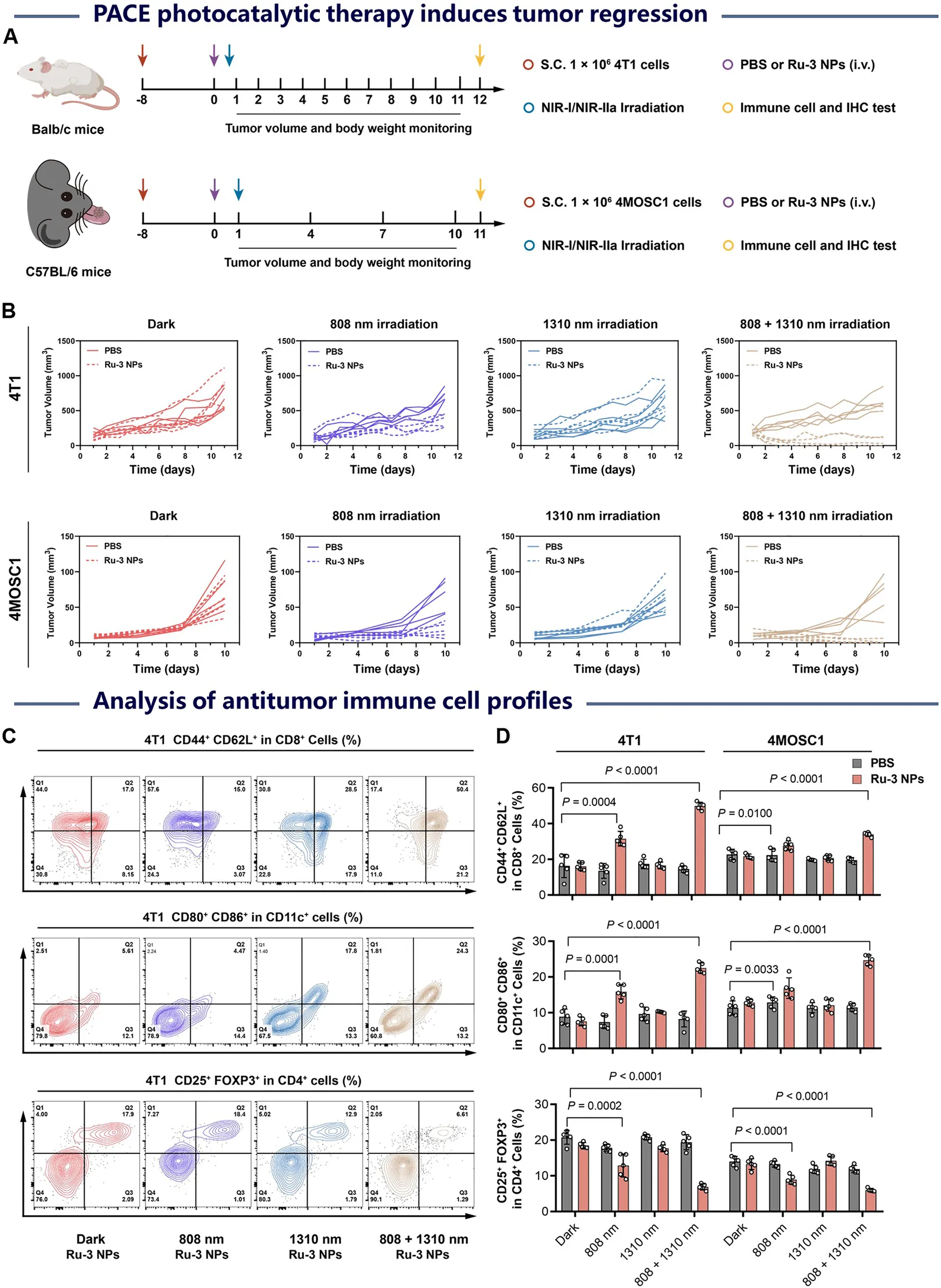
Therapeutic efficiency and immune effects in 4 T1 and 4MOSC1 xenograft model. (**A**) Scheme of tumor inoculation and treatment schedule in 4 T1 tumor-bearing Balb/c mice and 4MOSC1 tumor-bearing C57BL/6 mice. Eight days after tumor inoculation, mice were treated with PBS or **Ru-3 NPs** (45 μM, 200 μl). 808 nm and 1310 nm laser irradiation were performed the following day at a power intensity of 0.5 and 1.0 W cm^−2^, respectively. Tumors and tdLN were harvested 12 days after drug administration for immune cell and IHC test. Created in BioRender. lou, Z. (2026) https://BioRender.com/1kngn91. (**B**) Tumor growth curves of 4 T1 tumor-bearing mice and 4MOSC1 tumor-bearing mice with various treatments (*n* = 5). (**C** and **D**) Representative flow cytometry results (C) and analysis results (D) of the percentage of memory CD8^+^ T cell (CD44^+^CD62L^+^CD8^+^), dendritic cell (CD80^+^CD86^+^), and regulatory T cells (CD25^+^FOXP3^+^CD4^+^) isolated from the draining lymph node of 4 T1 tumor-bearing mice with various treatments (*n* = 5). Gating strategies are shown in fig. S46. For ANOVA tests with Dunnett’s multiple comparisons test, *P < 0.05; **P < 0.01; ***P < 0.001; ****P < 0.0001; ns, nonsignificant.

## DISCUSSION

In this work, we establish packing-accelerated consecutive excitation (PACE) as a previously unrecognized mode of NIR photoredox catalysis. Enabled by the self-assembling **Ru-3** complex, PACE couples aggregation-stabilized charge-separated states, long-lived triplet manifolds, and excited-state absorption in the NIR-IIa window to accesses a higher-energy triplet state, which enhances charge separation and supports efficient SET catalysis in aqueous and hypoxic environments.

PACE addresses the longstanding energetic and photophysical bottlenecks of NIR photocatalysis including insufficient photon energy, rapid charge recombination, and dependence on oxygen or organic media, and enables oxygen-independent photoredox conversion of NADH under hypoxic conditions. The resulting mitochondrial redox collapse induces mtDNA release, cGAS–STING activation, GSDMD-mediated pyroptosis, and M2-to-M1 macrophage polarization, ultimately generating potent antitumor immunity in multiple carcinoma models. Together, these findings position PACE as a generalizable strategy for achieving high-energy photochemistry with biologically compatible NIR light. By bridging the gap between deep-tissue optical penetration and high-energy catalytic reactivity, PACE opens new opportunities for remote-controlled redox modulation, immune engineering, and therapeutic intervention within complex biological systems.

## MATERIALS AND METHODS

### Computational details

All computational tasks were performed using the Gaussian 16 program (Revision A.03) with density functional theory (DFT) and time-dependent DFT (TD-DFT). Ground-state and excited-state structures were fully optimized using the PBE0 functional and a hybrid basis set comprising 6-31G* for ligand atoms and SDD for the Ru element. Dispersion effects were accounted for using the Becke-Johnson damping scheme (D3BJ). The range-separated functional CAM-B3LYP was employed to investigate electronic vertical transition processes, given its superior performance in calculating transitions with charge transfer (CT) characteristics. A mixed basis set (6-311G* for ligand atoms and SDD for the Ru element) was selected to balance calculation accuracy and computational cost. The SMD implicit solvent model was applied to simulate the DMSO environment, ensuring an accurate representation of solvent effects during calculations. Electron-hole analyses were performed using the Multiwfn (3.8 dev) program. Molecular structures and orbital visualizations were rendered using the VMD 1.9.3 program.

### TA spectroscopy

Transient absorption (TA) spectra were acquired using an automated femtosecond transient absorption spectrometer (Helios). The pump light was generated by a Coherent Astrella laser operating at 800 nm, with a pulse width of less than 100 fs and a repetition rate of 1 kHz. For wavelength selection, an optical parametric amplifier (OPerA-Solo) with a wavelength tuning range of 240–2600 nm was utilized. UV-VIS spectroscopy detection was carried out using a linear array back - illuminated thin CCD sensor. This sensor had a spectral coverage of 200–1000 nm, with specific bands of 320–650 nm and 420–780 nm. NIR spectra were recorded using a linear array InGaAs sensor, which had a detection range of 820–1600 nm. For sample preparation, **H7** or **Ru-3** was dissolved in either pure DMSO or a DMSO/H_2_O mixture (1/1, v/v) to a concentration of 150 μM. The prepared solutions were then transferred into quartz cuvettes with a path length of 1 mm for TA measurement.

### ROS measurement

The ROS level was assessed using the molecular probe DPBF, which undergoes decomposition in the presence of singlet oxygen. Detection was performed by monitoring changes in the absorbance of DPBF at 415 nm. Stock solutions of DPBF, **H7**, and **Ru-3** were freshly prepared prior to each experiment by dissolving the respective complexes in DMSO to a concentration of 1 mM. These stock solutions were subsequently diluted with DMSO and introduced into the aqueous phase according to a predefined DMSO/water volume ratio. The absorbance at 415 nm and 808 nm of the resulting solution was adjusted to approximately 2.0 and 0.3, respectively. The prepared solution was immediately transferred to a quartz cuvette for spectrophotometric analysis and irradiated using an 808 nm laser at a power intensity of 0.5 W cm^−2^. Intermittent irradiation cycles of 30 s were applied, with the absorption spectrum recorded after each irradiation step, until a cumulative irradiation time of 3 min was achieved. For comparative studies evaluating the efficiency of **Ru-3** and ICG, the irradiation time was reduced to 2 s, and the laser power intensity was lowered to 0.1 W cm^−2^, accounting for the higher efficiency of **Ru-3**_**50**_.

### Dual-wavelength optical irradiation platform

A custom dual-wavelength optical irradiation platform was constructed for co-linear 808 and 1310 nm near-infrared laser irradiation. The 808 and 1310 nm lasers were purchased from Blueprint Optics/Blueprint, Beijing, China. The optical system was assembled on an M6 optical breadboard (300 mm × 300 mm × 12.7 mm; LOTEK, MBB-3030) using post-based optomechanical components, including 50 mm and 100 mm post-holder assemblies (LOTEK, KPH-50-D and KPH-100-D), right-angle adapter plates (LOTEK, RAP-90A), a V-type clamp holder for cylindrical optical components (LOTEK, HM-V25), and a 45° optical mount for 25.4 mm optics (LOTEK, CM-3B3C)

The two laser beams were combined using a 25.4 mm UV-grade fused silica short-pass dichroic mirror with an 1180 nm transition wavelength (LOTEK, DM10-1180SP). The dichroic mirror was mounted at 45°, allowing transmission of the 808 nm beam and reflection of the 1310 nm beam, thereby merging the two beams into a shared optical path. Beam alignment was performed sequentially using a near-infrared detection card covering 700–1600 nm (85 mm × 20 mm × 1 mm; LOTEK, IRDC6). The 808 nm beam was first aligned through the dichroic mirror, followed by adjustment of the 1310 nm beam until both irradiation spots overlapped at the target plane.

For photochemical experiments, the NADH/**Ru-3** sample was placed at the calibrated beam output position and irradiated simultaneously with 808 and 1310 nm light (fig. S23). For in vivo experiments, Balb/c mice were positioned at the same calibrated irradiation plane, and the combined beam was directed to the designated treatment region (fig. S23). Before each experiment, beam overlap, irradiation position, laser power, power density, beam diameter, and exposure time were verified and kept consistent across experimental groups.

### EPR detection

All electron paramagnetic resonance (EPR) measurements were conducted using a Bruker Model A300 ESR spectrometer. The **Ru-3** DMSO stock solution was mixed with an equal volume of PBS buffer. This resulted in the formation of a **Ru-3** aggregate suspension at a concentration of 50 μM. For the detection of ROS, 2,2,6,6-tetramethylpiperidine (TEMP) at a concentration of 10 mM and 5,5-dimethyl-1-pyrroline *N*-oxide (DMPO) at a concentration of 50 mM were used. TEMP was employed to trap singlet oxygen, while DMPO was used to trap free radicals. Additionally, 2,2,6,6-tetramethylpiperidin-1-oxyl (TEMPO) at a concentration of 20 mM was used to measure the photo-generated electron-hole pairs in **Ru-3** aggregates. The prepared mixtures were irradiated. Irradiation was carried out using either an 808 nm laser with an intensity of 0.5 W cm^−2^ or a combination of an 808 nm laser (0.5 W cm^−2^) and a 1310 nm laser (1.0 W cm^−2^). To prevent thermal decomposition, the mixtures were placed in a water bath during the irradiation process. EPR spectra were recorded at 3 min and 6 min post-irradiation using the Bruker A300 spectrometer.

### ICP-MS

To assess the intracellular Ru content, 4 T1 cells were seeded in 100 mm tissue culture plates and incubated for 24 hours under standard conditions. Following incubation, the cells were treated with 10 μM **Ru-3 NPs** for 12 hours in complete RPMI-1640 medium. After treatment, the cells were washed three times with PBS to remove excess nanoparticles and subsequently detached using trypsin. The harvested cells were collected by centrifugation, and mitochondrial fractions were isolated utilizing a mitochondrial extraction kit (Beyotime, C3601) as per the manufacturer’s instructions. For ICP-MS analysis, both mitochondrial and non-mitochondrial cell fractions were subjected to acid digestion. Each sample was placed in a polytetrafluoroethylene crucible, and 10 ml of concentrated nitric acid was added. The crucibles were then covered and heated at 120°C for 120 min on a hot plate to ensure complete digestion. After cooling to room temperature, the digested samples were diluted to the desired final volume with ultrapure water. Quantification of ruthenium was subsequently conducted using an ICP-MS system (Agilent 7850) with the following operational settings: RF power at 1550 W, RF matching at 1.80 V, auxiliary gas flow rate at 1.0 liter/min, carrier gas flow rate at 1.0 liter/min, omega bias at −90 V, omega lens voltage at 10 V, cell entrance voltage at −30 V, and deflect voltage at 11 V.

### Dynamic light scattering

The hydrodynamic diameter and polydispersity index of **Ru-3 NPs** were measured by dynamic light scattering using a Zetasizer Nano ZS instrument (Malvern Panalytical, UK). Before measurement, **Ru-3 NP** suspensions were diluted in PBS, cell culture medium containing 10% FBS, or serum to a final concentration of 100 μg ml^−1^ **Ru-3** concentration and gently mixed to obtain a homogeneous dispersion. The dispersant was filtered through a 0.22 μm membrane filter before use to minimize dust contamination. Each sample was transferred into a disposable polystyrene cuvette and equilibrated at 25°C for 60 s. Measurements were performed with automatic attenuation and measurement positioning. The Z-average hydrodynamic diameter and PDI were calculated using the general-purpose analysis model. Each sample was measured in triplicate, and data are presented as mean ± SD.

### Photocatalytic oxidation of NADH

The photo-oxidation of NADH by **Ru-3 NPs** was analyzed using both ^1^H NMR spectroscopy and UV-Vis spectroscopy. For the ^1^H NMR analysis, **Ru-3 NPs** (0.1 mM) or **H7 NPs** (0.1 mM) were added to a D_2_O solution of NADH to achieve a final concentration of 3 mM. The ^1^H NMR spectra of the resulting solutions were recorded under four conditions: after 24 hours in darkness, after 30 min of 808 nm irradiation (0.5 W cm^−2^), after 20 min of 1310 nm irradiation (1.0 W cm^−2^), or after a combination of 808 nm and 1310 nm irradiation for 20 min. For the UV-Vis analysis, a stock solution of **Ru-3 NPs** (1 mM in PBS) was added to a quartz cuvette containing NADH in PBS, adjusting the final concentrations to 10 μM and 180 μM, respectively. The samples were exposed to darkness, 808 nm irradiation (0.5 W cm^−2^), 1310 nm irradiation (1.0 W cm^−2^), or a combination of both, each applied at 5-minute intervals. To minimize thermal effects, the irradiation process was conducted in a water bath at room temperature, with temperatures monitored using a photothermal camera (FORTRIC 225). The absorption spectrum was recorded after each irradiation step, up to a total irradiation time of 30 min. However, in the combined 808 nm and 1310 nm irradiation group, the absorbance at 260 nm exceeded the detection limit after 10 min. Therefore, another trial was conducted under the same conditions with 2-minute intervals. The turnover number (TON), representing the number of moles of NADH converted per mole of **Ru-3** within 60 min, was calculated. The NADH concentration was quantified using a standard curve (Y_260_ = 0.01708*X + 0.1274 in H_2_O). For the **Ru-3**_**50**_ photocatalytic capability test, a solvent condition of H_2_O: DMSO = 1:1 was selected, and the NADH concentration was determined based on the standard curve (Y_339_ = 0.002131*X - 0.002685 in H_2_O:DMSO).

### Electrochemical measurements

Cyclic voltammetry (CV) measurements of **Ru-3** were performed in deaerated DMSO containing 0.10 M tetrabutylammonium hexafluorophosphate (Bu_4_NPF_6_) as the supporting electrolyte, using a glassy carbon working electrode, a platinum counter electrode, and an Ag/AgCl reference electrode. Ferrocene was used as an internal reference and adjusting its value to zero. The first one-electron reduction potential of Ru-3 was determined as −0.14 V vs NHE. The excited-state reduction potential of **Ru-3** was estimated according to the equation

$$E_{\text{red}}^{*}\left(PS^{*}/PS^{\cdot -}\right)=E_{\text{red}}\left(\text{PS}/PS^{\cdot -}\right)+E_{0-0}$$

where $E_{\text{red}}\;\left(\text{PS}/PS^{\cdot -}\right)$ is the ground-state one-electron reduction potential of the photosensitizer and $E_{0-0}$ represents the excited-state energy estimated from the intersection of the normalized absorption and emission spectra. For **Ru-3**, the $E_{0-0}$ value was determined to be 1.35 eV, corresponding to the photoexcited state. Accordingly, the excited-state reduction potential of **Ru-3** was calculated to be +1.21 V vs NHE.

### Photocatalytic reduction of Fe^3+^-cyt c

The photocatalytic reduction of oxidized Fe^3+^-cyt c by **Ru-3 NPs** in the presence of NADH was investigated using UV-vis spectroscopy^15^. Fe^3+^-cyt c, **Ru-3 NPs**, and NADH were dissolved in the PBS solution to achieve a final concentration of 30 μM, 1 μM, and 15 μM. The resulting solution was then degassed by nitrogen purging for 30 min. The samples were exposed to darkness, 808 nm irradiation (0.5 W cm^−2^), 1310 nm irradiation (1.0 W cm^−2^), or a combination of both, each applied at 5-minute intervals. The absorption spectrum was recorded after each irradiation step, up to a total irradiation time of 30 min.

### Cell viability

The viability of 4 T1 cells was evaluated using the CCK-8 assay. Cells in the exponential growth phase were seeded at a density of 5000 cells per well in 96-well plates and incubated at 37°C in a normoxic environment (5% CO_2_, 95% air) for 24 hours. **Ru-3 NPs** were diluted in complete RPMI 1640 medium to final concentrations of 0.5, 1, 5, 10, 25, and 50 μM and then added to the wells. After 12 hours of incubation, the medium was replaced with drug-free medium. The light-treated groups were subjected to 808 nm irradiation (0.5 W cm^−2^), 1310 nm irradiation (1.0 W cm^−2^), or a combination of both for 10 minutes. Following irradiation, cells were allowed to recover for 36 hours before the CCK-8 assay was performed.

### ATP release detection

4 T1 cells were seeded at a density of 2 × 10^5^ cells per well in 24-well plates and cultured. After cell adhesion, the medium was replaced with a serum-free medium, and the designated treatment was applied. After 24 hours of stimulation, the supernatant was collected for subsequent analysis. ATP release in each group was quantified using an ATP detection kit (Beyotime).

### LDH release detection

4 T1 cells were seeded in a 96-well plate at a density of 5 × 10^3^ cells per well and incubated for 12 hours. A fresh medium without FBS was added after adhesion, and the relevant treatment was administered. The plate was then incubated at 37°C for 24 hours, with untreated cells serving as the control group. To measure LDH release, supernatants were collected from each well after centrifugation at 400 g for 5 min, and 120 μl of the supernatant was transferred to a new 96-well plate. Subsequently, 60 μl of LDH detection working solution (Beyotime) was added to the plate and incubated at 37°C for 30 min. The absorbance was finally measured at 490 nm using a microplate reader.

### Stable cell line generation

Single guide RNAs (sgRNAs) targeting GSDMD and MLKL were cloned into the Lenti-CRISPRv2 vector. Lentivirus was packaged in 293 T cells. 4 T1 cells were infected with lentivirus containing the respective sgRNAs. After 36 hours, 4 T1 cells were selected with 5 μg ml^−1^ puromycin for 2 days. Cells were subsequently maintained in culture medium containing puromycin (Sigma-Aldrich). The sgRNA sequences are as follows:

Gsdmd sgRNA 1: 5′-CACCGCAACAGCTTCGGAGTCGTG-3′,

Mlkl sgRNA 1: 5′-CACCGCCCTCCCAACGGCCCTCTG-3′.

### Western blot analysis

Western blot procedures were conducted as previously described. In summary, the protein concentration of the extracted sample was determined, and the samples were loaded onto 5% SDS-PAGE concentrated gels for stacking and 12% SDS-PAGE separating gels for separation. Following electrophoresis, proteins were transferred onto PVDF membranes and then incubated with 5% skimmed milk at room temperature for 1 hour, followed by overnight incubation with primary antibody solutions at 4°C. The next day, the membranes were incubated with the appropriate secondary antibodies for 1 hour, and the Odyssey system (LI-COR Biosciences) was used to visualize the image employing an ECL kit (Advansta). For protein quantification, ImageJ software (National Institutes of Health, Bethesda, Maryland, USA) was employed. The experiment was repeated at least three times. Primary antibodies against GSDMD (ab219800; Abcam), Phospho-STING (72971; Cell Signaling Technology), cGAS (79978; Cell Signaling Technology), and beta-Actin (ab8226; Abcam) were used.

### Immunohistochemistry staining

Immunohistochemistry staining was conducted following an established protocol. Briefly, 4 μm paraffin-embedded sections of tumor tissue underwent dewaxing and rehydration. Antigen retrieval was achieved by heating the sections in a microwave oven using either citrate acid solution or EDTA. The sections were then treated with 3% hydrogen peroxide and blocked with goat serum. Primary antibodies were applied to the sections and incubated overnight at 4°C. Primary antibody against CD8 (98941, Cell Signaling Technology) was utilized. Subsequently, the sections were incubated with a secondary biotinylated IgG antibody followed by anti-biotinyl-peroxidase reagent for 20 min. Signal detection was accomplished using a DAB reagent (Mxb Biotechnologies), and nuclei were counterstained with hematoxylin. Slide scanning was performed using a Pannoramic Midi scanner (3DHISTECH), and CaseViewer v. 2.4 (3DHISTECH) was used to create slide presentations. Histoscore analysis of the detection indicators was conducted utilizing QuantCenter software v. 12.3.2 (3DHISTECH).

### Immunofluorescence staining

For immunofluorescence staining, cells were fixed using 4% paraformaldehyde, permeabilized with 0.2% Triton X-100, and then blocked with 5% bovine serum albumin for 1 hour. Following the blocking step, cells were incubated overnight at 4°C with specific primary antibodies. After washing the cells three times with PBS, they were incubated with fluorophore-conjugated secondary antibodies (Abbkine) for 1 hour. Nuclei were counterstained with DAPI (Beyotime). The acquired images were captured using a Confocal Laser Scanning Microscope and analyzed using FV10-ASW Viewer software (OLYMPUS). The specific primary antibodies used were anti-ZBP1 (AG-20B-0010, AdipoGen), Anti-Z-DNA/Z-RNA (Ab00783–3.0, Absolute Antibody), and anti-HMGB1 (6893, Cell Signaling Technology).

### Quantitative real-time polymerase chain reaction (qRT-PCR)

RNA from cells was isolated employing TRIzol reagent (Life Technologies), with all steps carried out on ice to minimize RNA degradation. The RNA’s quality and purity were verified via spectrophotometry (ratios of A260/A280 and A260/A230), while its integrity was checked via gel electrophoresis. For reverse transcription, 1 μg of the extracted RNA was utilized, generating cDNA with the Hiscript II Q RT SuperMix kit (Vazyme, China). Quantitative real-time PCR (qRT-PCR) was conducted to measure relative mRNA levels, using SYBR Green master mix (Vazyme, China) on a QuantStudio 6 Real-Time PCR System (Life Technologies). Expression fold changes were calculated using the 2^-ΔΔCT method, normalizing each cDNA sample against GAPDH expression. Primer details are provided in table S5. To effectively illustrate the qRT-PCR results, we used the R Studio software heatmap function to plot the normalized gene expression data. The heatmap’s color palette was strategically chosen to represent the continuum of expression levels: shades of blue denoted downregulated gene expression compared to the control, while a progression towards red signified upregulated expression. This method produced a visually intuitive gradient, facilitating an immediate perception of gene expression alterations.

### In vivo antitumor study and dual-wavelength irradiation

For the subcutaneous breast tumor model, 4 T1 cells were harvested during the logarithmic growth phase, washed with sterile PBS, and resuspended in PBS. Each Balb/c mouse was inoculated with 1 × 10^6^ 4 T1 cells by subcutaneous injection into the right hind flank. For the orthotopic oral tumor model, 1 × 10^6^ 4MOSC1 cells were injected orthotopically into the tongue of female C57BL/6 mice. The cell suspension volume, buffer composition, and whether Matrigel was used were 50 μl PBS.

Eight days after tumor inoculation, the mice were randomly divided into eight groups (*n* = 5 per group). Mice were intravenously administered PBS or **Ru-3 NPs** through tail vein injection. The dose of **Ru-3 NPs** was 45 μM in a total injection volume of 200 μl per mouse. The treatment groups were as follows: PBS, PBS + 808 nm irradiation, PBS + 1310 nm irradiation, PBS + 808/1310 nm irradiation, **Ru-3 NPs**, **Ru-3 NPs** + 808 nm irradiation, **Ru-3 NPs** + 1310 nm irradiation, and **Ru-3 NPs** + 808/1310 nm irradiation.

For laser-treated groups, mice were irradiated for 10 min at the designated tumor region. The 808 nm laser was applied at a power density of 0.5 W cm^−2^, and the 1310 nm laser was applied at a power density of 1.0 W cm^−2^. For the dual-wavelength treatment group, 808 and 1310 nm irradiation were applied simultaneously using the homemade dual-wavelength optical irradiation platform. During irradiation, mice were anesthetized with isoflurane and positioned at the calibrated irradiation plane, and the laser spot was directed to cover the tumor region. Laser power was calibrated using a FieldBest-I laser power meter (5 mW^−2^ W range; Fieldbest).

Tumor size was monitored for 10 days after treatment. Tumor length and width were measured using vernier scale, and tumor volume was calculated using the following equation

$$\text{Tumor volume}=\left(\text{length}\times {\text{width}}^{2}\right)/2$$

Mice were euthanized by carbon dioxide inhalation when the tumor diameter exceeded 2 cm in any direction or when other humane endpoints were reached according to the approved animal protocol.

### Flow cytometry

tdLN were collected at the endpoint of therapy and converted into single-cell suspensions. Subsequently, these cells underwent staining with specific antibodies before being subjected to analysis. The analysis and visualization of the study’s findings were conducted using FlowJo 10 software (Version 10.0.6, Tree Star). For fluorescence labeling, the antibodies used included Fixable Viability Dye (eFluor 506, eBioscience), anti-CD45 (APC-CY7, eBiosciences), anti-CD3 (FITC, eBiosciences), anti-CD4 (eFluor450, eBiosciences), anti-CD8 (PerCP-Cyanine 5.5, BioLegend), anti-CD11c (FITC, eBiosciences), anti-CD80 (PE, eBiosciences), anti-CD86 (APC, eBiosciences), anti-MHC-II (PE-Cyanine7, eBioscience), anti-CD25 (APC, eBioscience), anti-Foxp3 (PE, eBioscience), anti-CD44 (PE, eBiosciences), and anti-CD62L (APC, eBiosciences) following the manufacturers’ instructions.

## Supplementary Materials

### This PDF file includes:

Supplementary Text NMR and mass spectra Figs. S1 to S47 Tables S1 to S5
