# Supplementary material for: Packing-accelerated consecutive NIR excitation (PACE) enables high energy photocatalysis and antitumor immunity in vivo
Source: Sci Adv. 2026 Oct 7;12(41):eaeg3842. doi: 10.1126/sciadv.aeg3842 (PMC13644746; doi:10.1126/sciadv.aeg3842)
Supplement: Supplementary file 1 [file sciadv.aeg3842_sm.pdf]

Science Advances
AAAS

Supplementary Materials for

Packing-accelerated consecutive NIR excitation (PACE) enables high energy photocatalysis and antitumor immunity in vivo

Yishen Liu *et al.*

Corresponding author: Xinyuan Fan, xyfan@bnu.edu.cn; Xuechuan Hong, hongxc@fudan.edu.cn; Zhijun Sun, sunzj@whu.edu.cn; Yuling Xiao, xiaoyl@whu.edu.cn

Sci. Adv. **12**, eaeg3842 (2026)
DOI: 10.1126/sciadv.aeg3842

This PDF file includes:

Supplementary Text
NMR and mass spectra
Figs. S1 to S47
Tables S1 to S5

Supplementary Text

General Information

All reagents and solvents were commercially procured from suppliers, including Innochem, Adamas Aldrich, and Sinopharm Group Co., Ltd., and were used without further purification. PEO_{114}-b-PS_{100} was obtained from Xian Ruixi Biological Technology. Nuclear magnetic resonance (NMR) spectra for ^{1}H and ^{13}C were recorded using a Bruker AVANCE400 spectrometer in $CDCl_3$, DMSO-d_6, or D_2O at room temperature. The ESI-MS spectrum was acquired using a Triple QuadTM 7500 system (Sciex), while MALDI-TOF-MS analysis was conducted on an AB Sciex 5800 MALDI-TOF/TOF instrument. Absorption spectra and steady-state photoluminescence spectra were measured using a SHIMADZU UV-2600 and an Edinburgh FLSP920 fluorescence spectrophotometer, respectively. Electron paramagnetic resonance (EPR) spectra were recorded on a Bruker A300 spectrometer. Cellular fluorescence images were captured with a Leica TCS SP8 STED CW confocal microscopy system.

The murine breast cancer cell line 4T1, obtained from the American Type Culture Collection (ATCC), was cultured in RPMI 1640 medium supplemented with 10% fetal bovine serum (FBS) and 1% penicillin. Experimental work with the 4MOSC1 and RAW264.7 cell lines was performed at the School of Stomatology, Wuhan University, under the supervision of Prof. Zhijun Sun. The 4MOSC1 cell line, provided by Prof. J. Silvio Gutkind from the University of California, San Diego, under a material transfer agreement (SD2017-202), was cultured in serum-free keratinocyte medium (K-SFM, Gibco-BRL). All cell lines were maintained under standard culture conditions at 37°C in a humidified incubator with 5% CO_2. For animal experiments, female BALB/c and C57BL/6 mice (6–8 weeks old) were obtained from the Hubei Provincial Academy of Preventive Medicine. Ethical approval for all in vivo experiments was granted by the Animal Ethics Committee of the Laboratory Animal Centre at Huazhong Agricultural University (Approval Number: HZAUMO-2023-0278) and the Institutional Animal Care and Use Committee (IACUC), Wuhan University Center for Animal Experiment, Wuhan, China (Approval Number: WP20220249). All animal procedures were conducted in strict compliance with the Regulations for the Administration of Affairs Concerning Experimental Animals, as approved by the State Council of the People's Republic of China.

1. Preparation of H7, Ru-1 and Ru-3

1a. Synthesis of intermediate 3

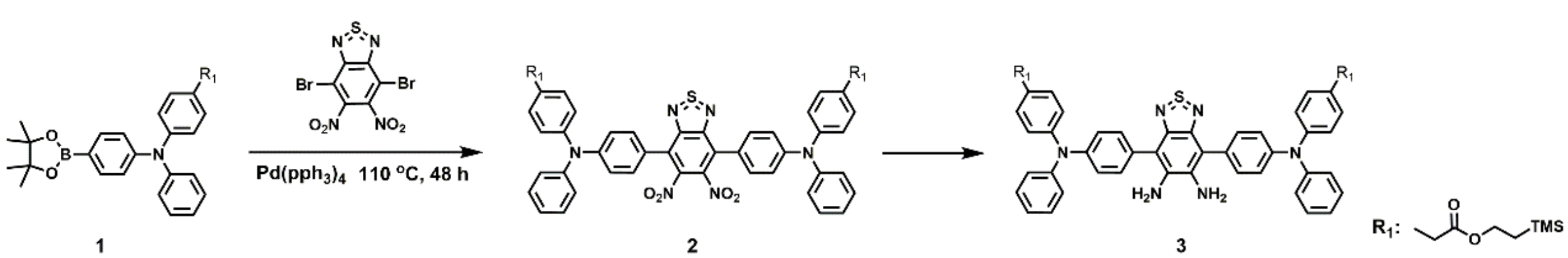

Compound 2: Compound **1** (504 mg, 0.93 mmol) was synthesized according to established procedures. A mixture of compound 1,4,7-dibromo-5,6-dinitrobenzothiadiazole (142 mg, 0.46 mmol), and K_2CO_3 (410.4 mg, 2.5 mmol) in 20 mL of toluene and 5 mL of water was prepared. $Pd(PPh_3)_4$ (107.16 mg, 0.09 mmol) was then added under a nitrogen atmosphere, and the reaction was stirred at 115 °C in an oil bath for 48 h. Upon completion, the reaction mixture was cooled to room temperature, diluted with 100 mL of water, and extracted three times with 50 mL portions of ethyl acetate. The combined organic layers were dried over anhydrous Na_2SO_4, and the drying

agent was removed by filtration. The filtrate was concentrated under reduced pressure using rotary evaporation to afford a crude product. The crude mixture was purified by silica gel chromatography (eluent: petroleum ether/ethyl acetate = 5/1, v/v), yielding compound **2** (112 mg, 23% yield).

^{1}H NMR (400 MHz, $CDCl_3$): 7.43 - 7.37 (m, 4H), 7.35 - 7.29 (m, 4H), 7.22 - 7.06 (m, 18H), 4.24 - 4.14 (m, 4H), 2.94 (t, *J* = 7.8 Hz, 4H), 2.63 (dd, *J* = 8.5, 7.1 Hz, 4H), 1.02 - 0.95 (m, 4H), 0.05 (s, 18H).

Compound 3: The resulting compound **2** (80 mg, 0.076 mmol) was dissolved in a mixture of dichloromethane (5 mL), methanol (5 mL), and water (0.5 mL). Ammonium chloride (NH_4Cl, 146 mg, 2.73 mmol) and zinc powder (594 mg, 9.08 mmol) were subsequently added to the solution. The reaction was carried out at 25 °C for 2 h, after which the reaction mixture was filtered. The filter cake was thoroughly washed with 20 mL of dichloromethane, and the combined filtrate was concentrated under reduced pressure using a rotary evaporator. This process yielded a yellow crude product (**3**), which was directly utilized in the subsequent synthetic steps.

1b. Synthesis of compound H7

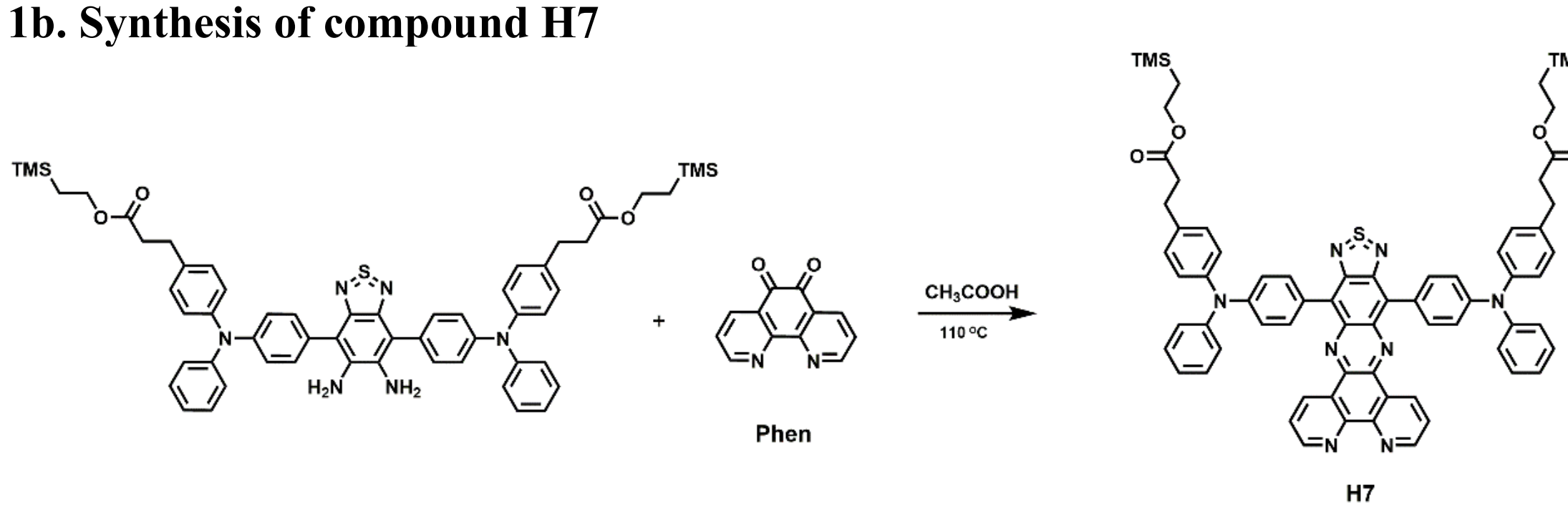

Compound H7: Compound **3** (95 mg, 0.08 mmol) and 1,10-phenanthroline-5,6-dione (30 mg, 0.14 mmol) were dissolved in 15 mL of acetic acid. The resulting mixture was refluxed in an oil bath at 120 °C for 2 h. Upon completion, the reaction mixture was allowed to cool to room temperature and was concentrated under reduced pressure using a rotary evaporator to afford a crude residue. The residue was purified by silica gel chromatography (eluent: petroleum ether/ethyl acetate = 5/1, v/v), yielding the desired compound H7 as a green solid (42 mg, 45% yield).

^{1}H NMR (400 MHz, $CDCl_3$): 9.23 (d, *J* = 7.0 Hz, 4H), 8.06 - 7.99 (m, 4H), 7.72 (t, *J* = 6.3 Hz, 2H), 7.41 - 7.33 (m, 12H), 7.28 (s, 4H), 7.23 (d, *J* = 8.6 Hz, 4H), 7.15 (tt, *J* = 6.9, 1.6 Hz, 2H), 4.28 - 4.18 (m, 4H), 3.00 (t, *J* = 7.8 Hz, 4H), 2.69 (dd, *J* = 8.5, 7.1 Hz, 4H), 1.07 - 0.98 (m, 4H), 0.07 (s, 18H).

1c. Synthesis of $Ru(Phen)_3(PF_6)$

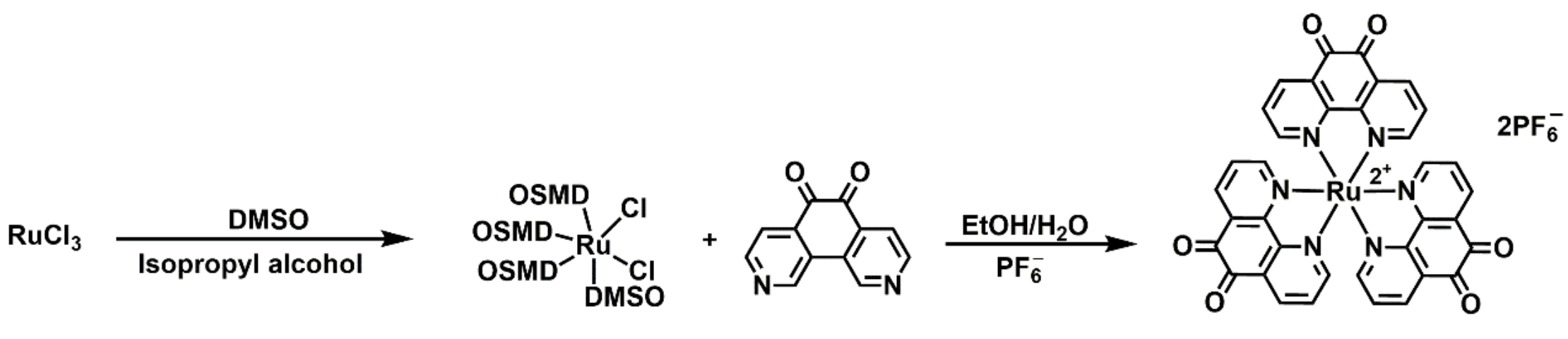

Phen Ru(Phen)$_3$(PF$_6$)$_2$

Ru(DMSO)$_4$Cl$_2$: In a 100 mL Schlenk flask, RuCl$_3$·xH$_2$O (0.8 g, 3.85 mmol) and DMSO (2.5 mL) were combined and degassed for 20 min. Subsequently, isopropanol (17.5 mL) was added under a nitrogen atmosphere. The reaction mixture was heated at 90 °C for 48 h and then allowed to cool to room temperature. The resulting yellow solid was collected by filtration and thoroughly washed successively with acetone, toluene, and diethyl ether. After vacuum drying, Ru(DMSO)$_4$Cl$_2$ was obtained as a crude product (~1.63 g) without further purification.

Ru(Phen)$_3$(PF$_6$)$_2$: Ru(DMSO)$_4$Cl$_2$ (420 mg, 0.87 mmol) and 1,10-phenanthroline-5,6-dione (624 mg, 2.97 mmol) were dissolved in 15 mL of H$_2$O/EtOH (1:1, v/v). The reaction mixture was refluxed for 10 h, and the product was isolated through centrifugation. To the residual solvent, 500 mg of KPF$_6$ was added, and the product was precipitated as a PF$_6^-$ alt in water.

^{1}H NMR (400 MHz, CD$_3$CN): 8.62 (d, J = 8.0 Hz, 6H), 8.14 (m, 6H), 7.72 (dd, J = 8.0, 5.6 Hz, 6H). Calcd for: C$_{36}$H$_{18}$N$_6$O$_6$Ru ([M-2PF$_6$]$^{2+}$): 366.01, found: 366.01.

1d: Synthetic route of Ru-3

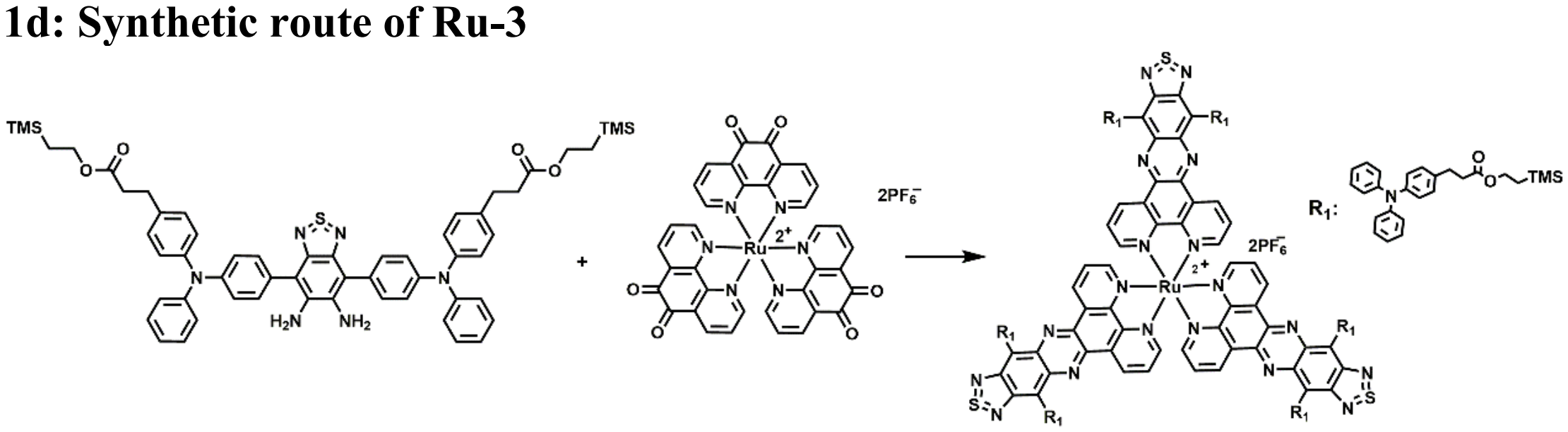

Ru-3: Compound **3** (94 mg, 0.08 mmol) and Ru(Phen)$_3$(PF$_6$)$_2$ (19 mg, 0.026 mmol) were dissolved in 8 mL of acetic acid. The reaction mixture was refluxed in an oil bath at 120 °C for 4 h and then allowed to cool to room temperature. Subsequently, the mixture was diluted with 100 mL of water and extracted with ethyl acetate (3 × 50 mL). The combined organic layers were dried over anhydrous Na$_2$SO$_4$, and the drying agent was removed by filtration. The filtrate was concentrated under reduced pressure using rotary evaporation to afford a crude product. The crude mixture was purified by silica gel column chromatography using dichloromethane/methanol (10:1, v/v) as the eluent, yielding **Ru-3** as a dark green solid (19 mg, 21% yield).

^{1}H NMR (400 MHz, CDCl$_3$): 9.27 (dd, J = 8.2, 1.3 Hz, 6H), 8.48 (d, J = 5.5 Hz, 6H), 8.00 - 7.92 (m, 12H), 7.89 (dd, J = 8.2, 5.5 Hz, 6H), 7.31 - 7.26 (m, 22H), 7.25 - 7.10 (m, 34H), 7.05 (tt, J = 6.8, 1.6 Hz, 10H), 4.15 - 4.09 (m, 12H), 2.89 (t, J = 7.8 Hz, 12H), 2.57 (dd, J = 8.6, 7.1 Hz, 12H), 0.96 - 0.90 (m, 12H), -0.02 (s, 54H). ^{13}C NMR (101 MHz, CDCl$_3$) δ 174.6, 157.7, 154.9, 152.4, 150.3, 148.6, 146.7, 135.5, 130.9, 129.3, 127.3, 127.0, 122.0, 115.4, 64.2, 54.9, 37.5, 33.4, 31.9, 31.2, 30.8, 28.7, 24.2, 18.8, 15.6, 2.5, 0.0. MALDI-TOF MS Calcd for: C$_{210}$H$_{198}$F$_6$N$_{24}$O$_{12}$PRuS$_3$Si$_6^+$ ([M-PF$_6$]$^+$): 3759.21, found: 3759.74.

1e. Preparation of H7 and Ru-3 dots

The **H7** dots and **Ru-3** dots were synthesized according to the previously reported methods. The stock solutions of **H7** and **Ru-3** were prepared by dissolving them in anhydrous THF. A concentration of 1 mg/mL was used for both compounds in this process. Subsequently, 1 mL of each stock solution was rapidly injected into 10 mL of deionized water using a syringe. The resulting mixture was continuously stirred for approximately 10 seconds. To eliminate THF from the suspension, the resulting mixture was placed in a 90 °C oil bath. The suspension was left in the oil bath for 9 hours, during which the removal of THF occurred.

1f. Preparation of Ru-3 NPs

Stock solutions were formulated by dissolving **Ru-3** and PEO_{114}-b-PS_{100} in THF. **Ru-3** was dissolved at a concentration of 0.6 mg/mL, while PEO_{114}-b-PS_{100} was dissolved at a concentration of 6 mg/mL. The THF-phase solution was prepared in accordance with a standardized protocol. Specifically, 10 μL each of the **Ru-3** and the PS-polymer stock solutions were combined. The volume of THF in the resulting THF-phase solution was adjusted to achieve the prescribed water/THF ratio. A 20 mL sample vial was used to carry out the subsequent steps. The THF-phase solution was added to this vial. Then, 500 μL of the water phase was added directly into the polymer solution in the vial in a single portion. The mixture in the vial was stirred at room temperature. During this process, THF gradually evaporated from the mixture. After the residual THF had dissipated, additional water was introduced into the vial. This was done until the final volume of the solution in the vial reached 500 μL.

2. NMR and Mass Spectra

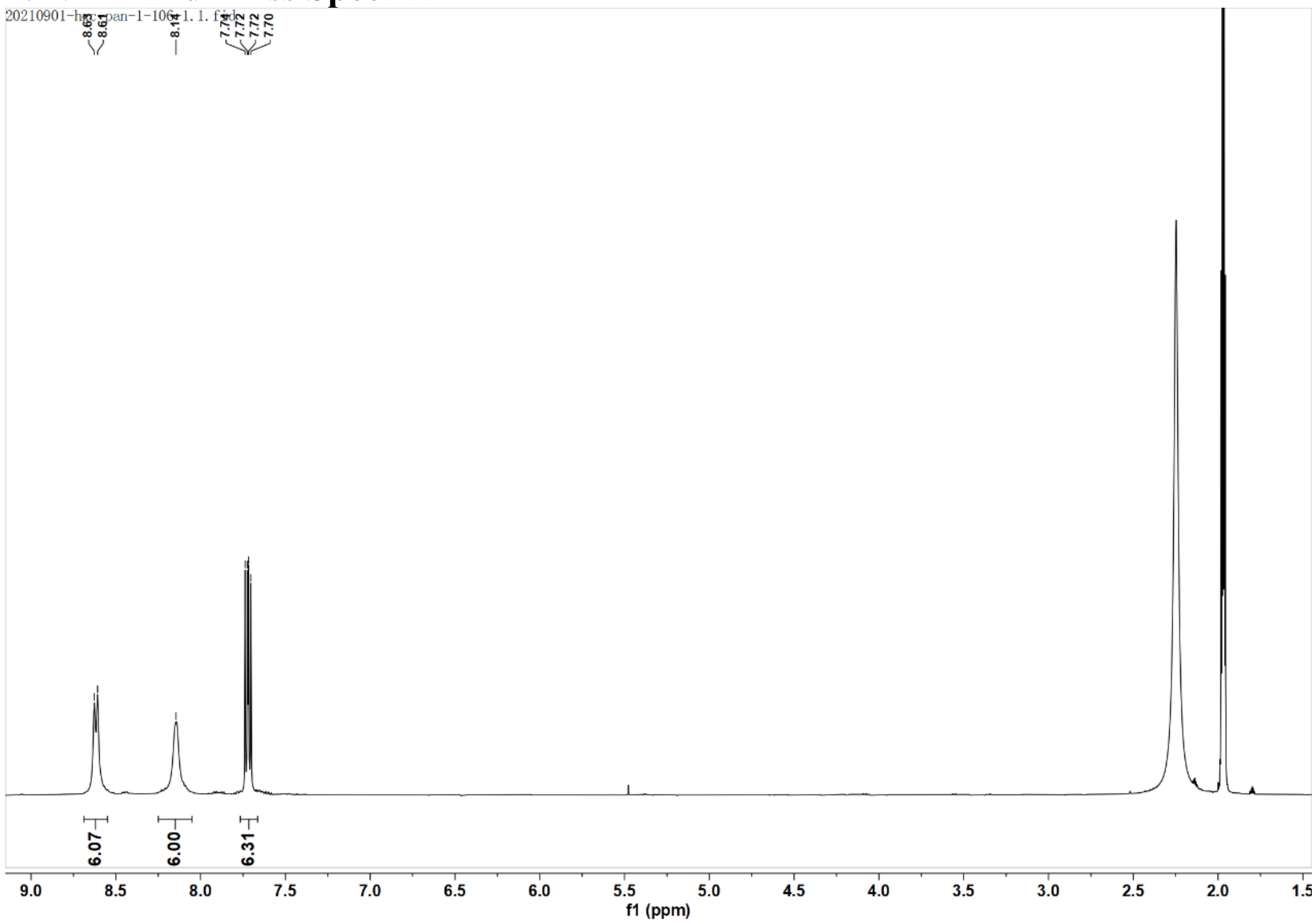

Fig. S1. ^{1}H NMR spectra of the $Ru(Phen)_3(PF_6)_2$ in CD_3CN.

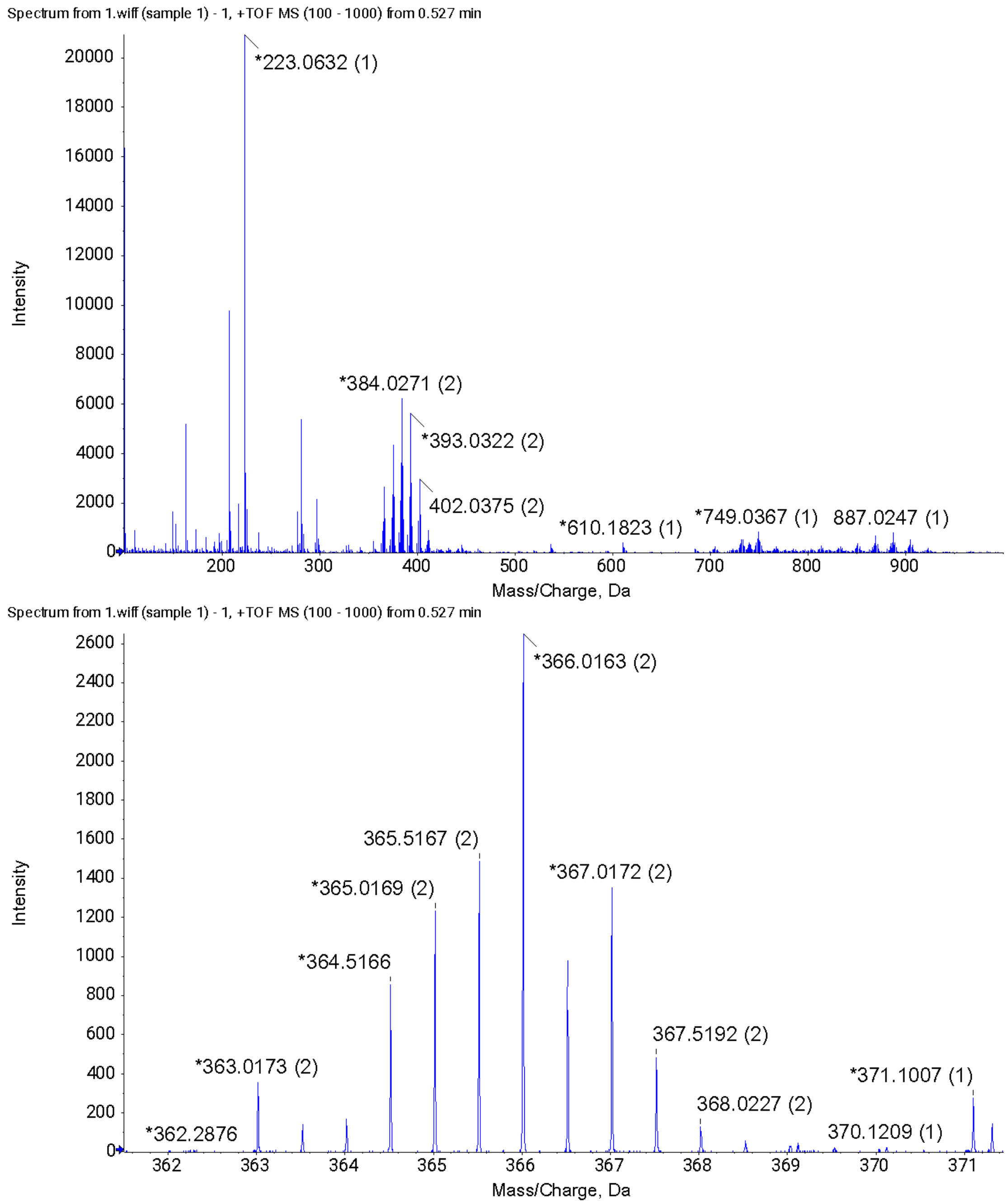

Fig. S2. Electrospray ionization mass spectrometry of the Ru(Phen)$_3$(PF$_6$)$_2$. Calcd for: $C_{36}H_{18}N_6O_6Ru$ ($[M-2PF_6+H_2O]^{2+}$): 384.02, found: 384.02. $C_{36}H_{18}N_6O_6Ru$ ($[M-2PF_6]^{2+}$): 366.01, found: 366.01.

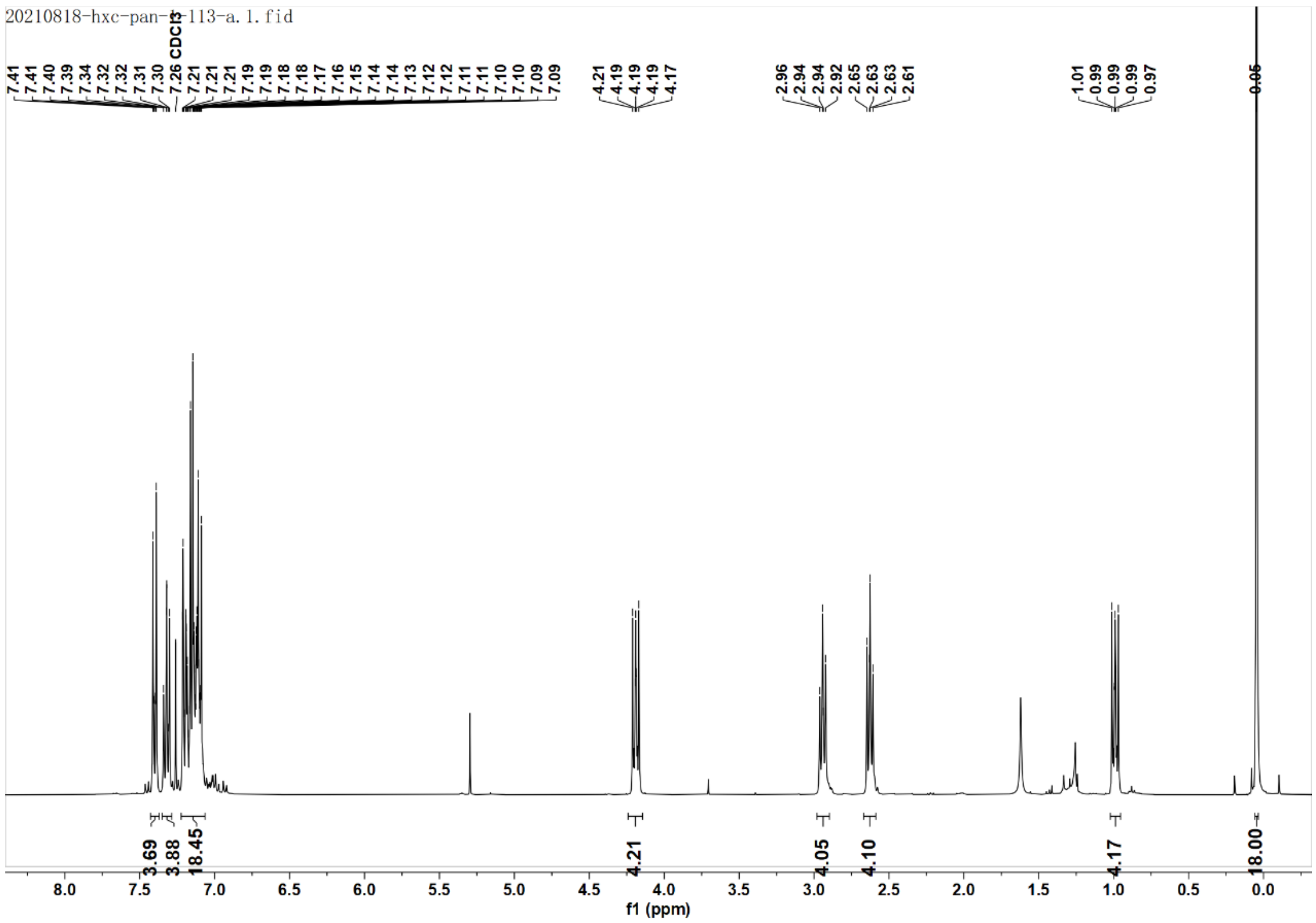

Fig. S3. ^{1}H NMR spectra of compound 2 in $CDCl_3$.

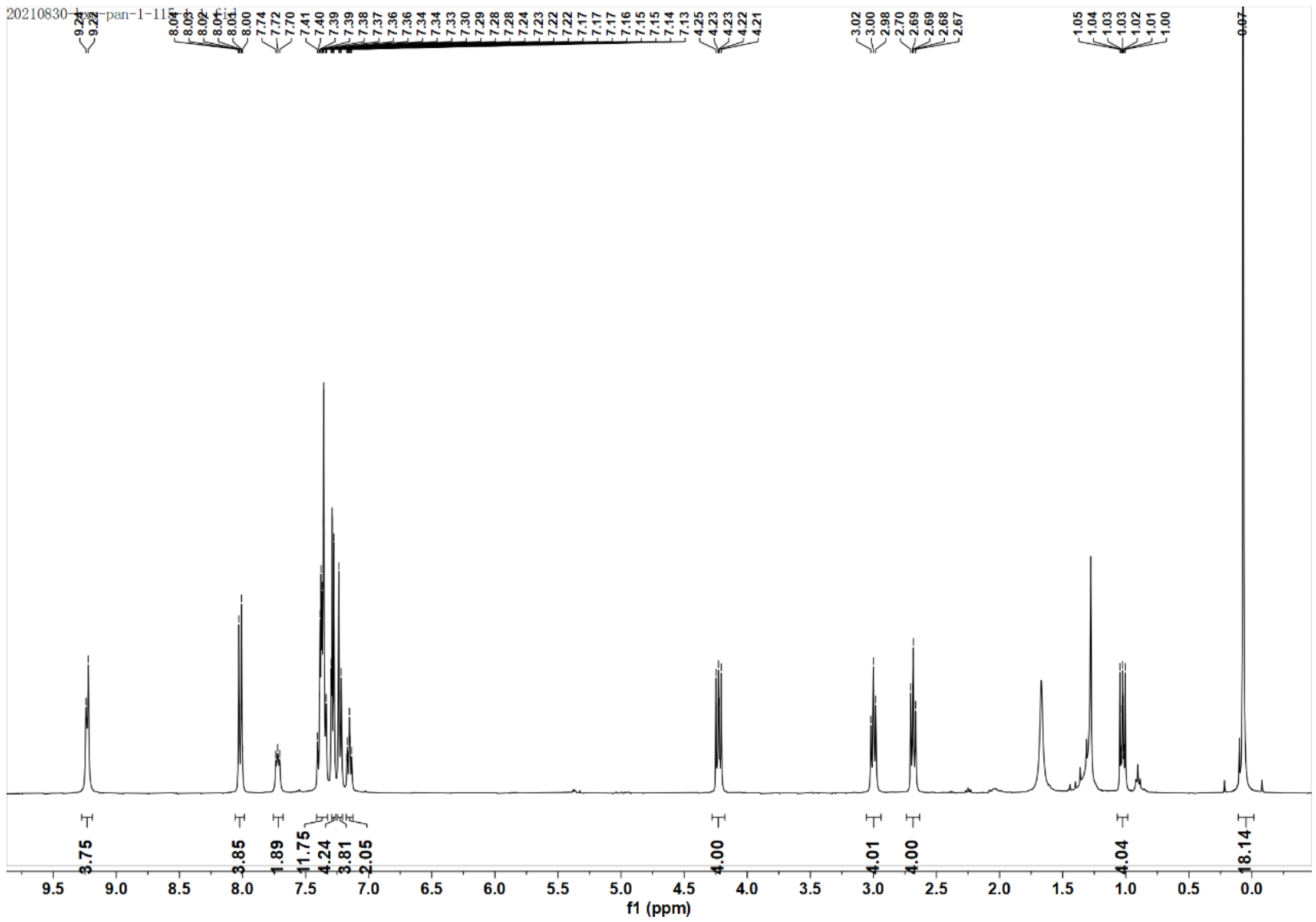

Fig. S4. ^{1}H NMR spectra of the H7 in CDCl$_3$.

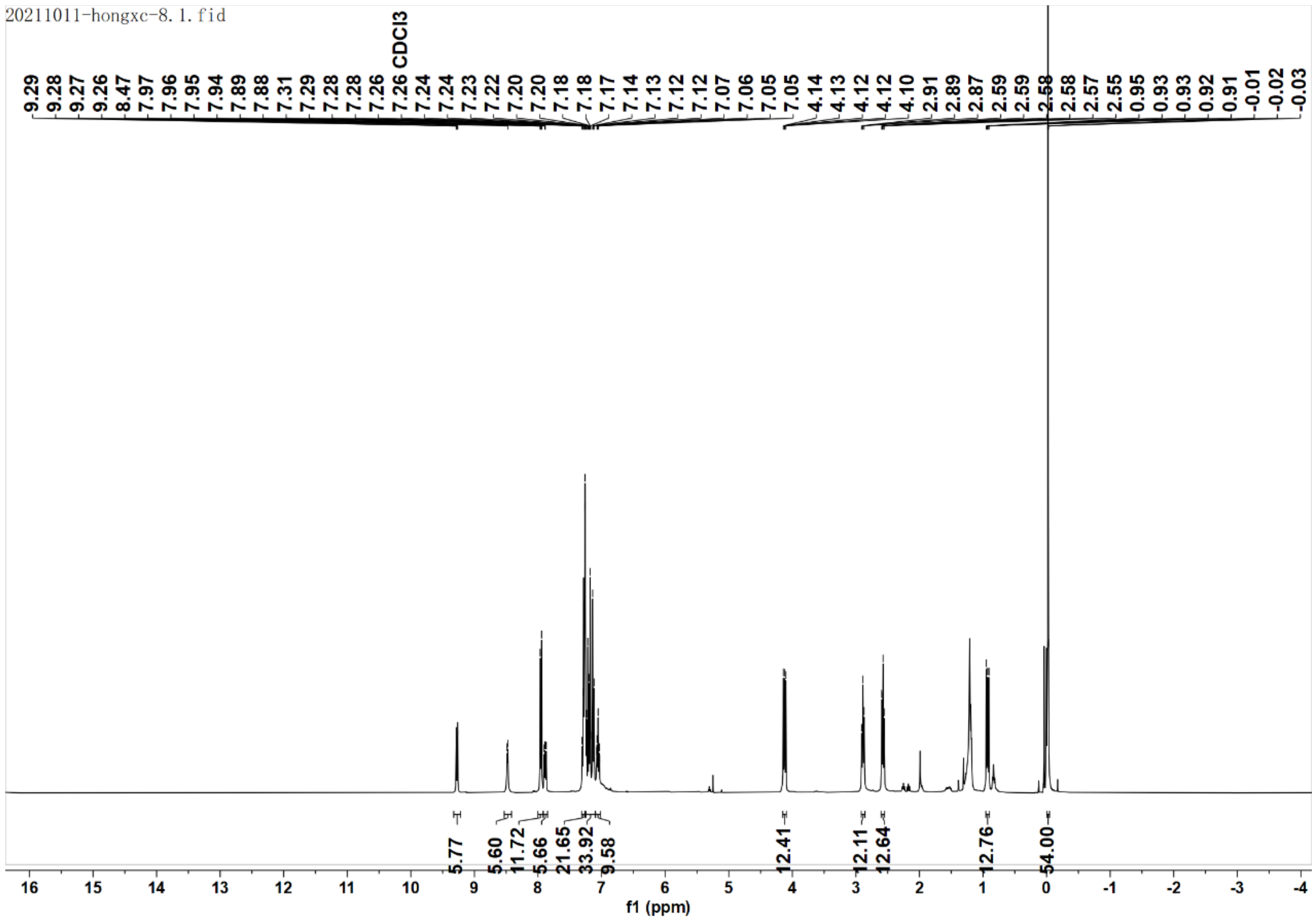

Fig. S5. ^{1}H NMR spectra of the Ru-3 in $CDCl_3$.

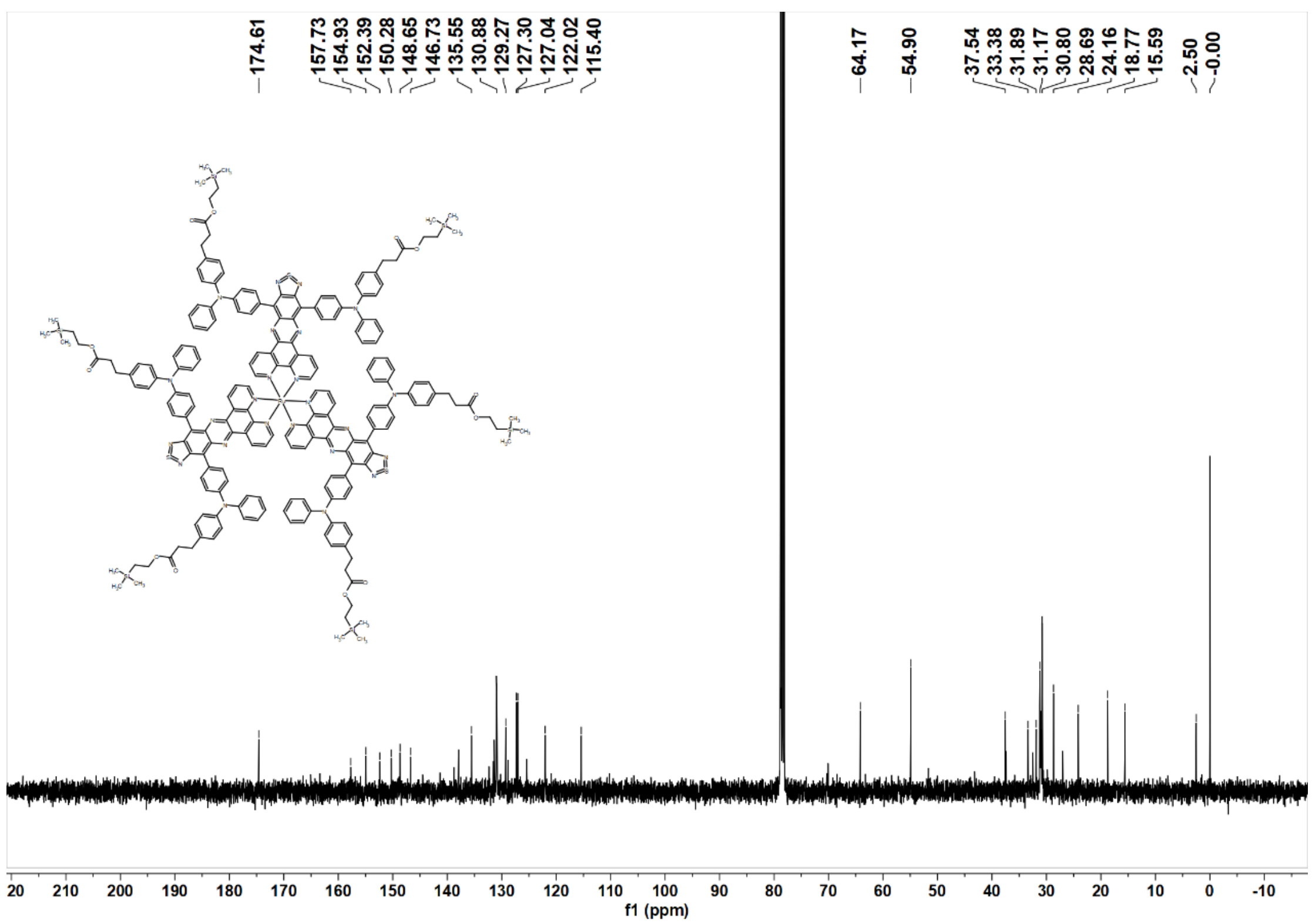

Fig. S6. ^{13}C NMR spectra of Ru-3 in $CDCl_3$.

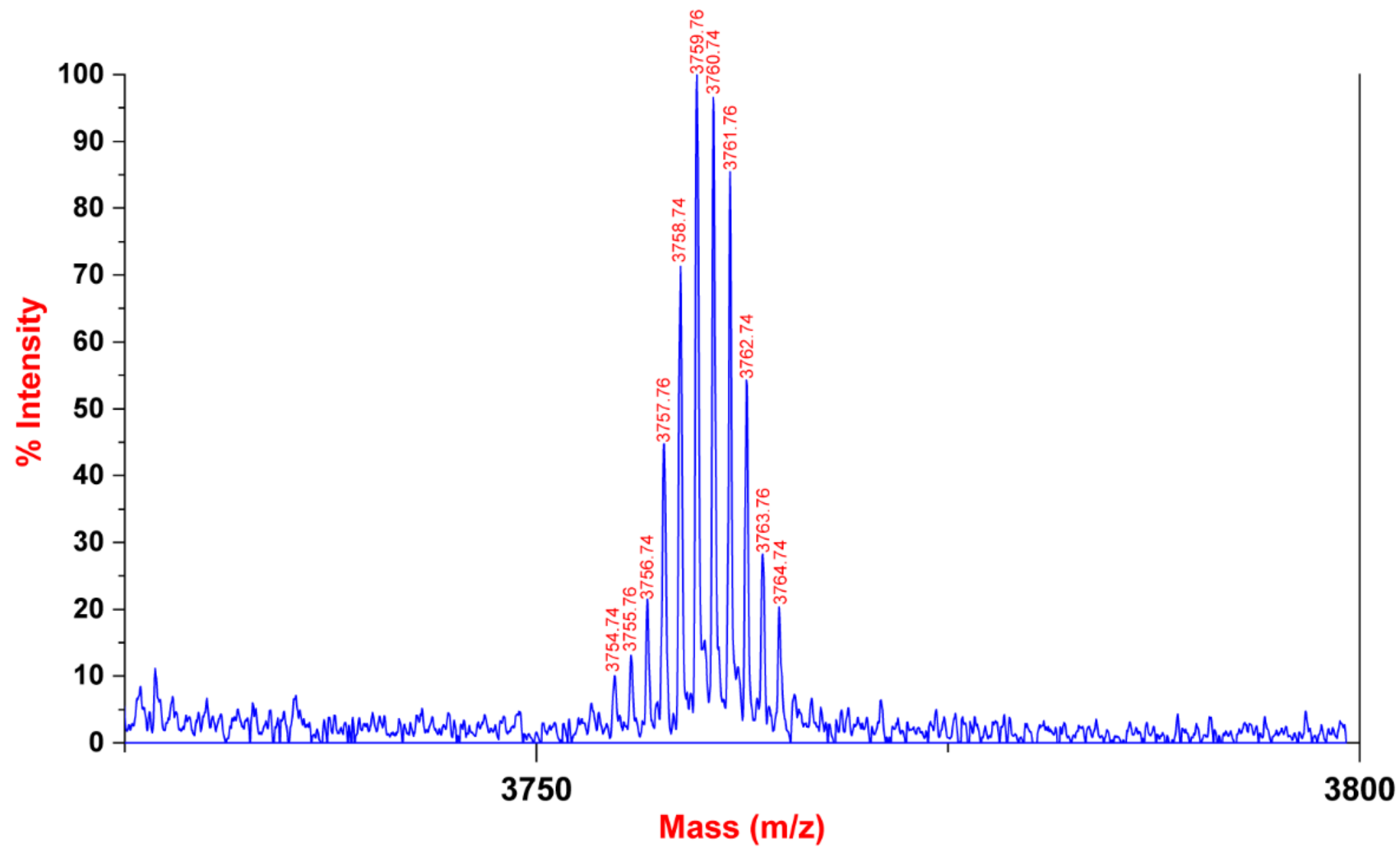

Fig. S7. MALDI-TOF-MS of Ru-3. Calcd for: $C_{210}H_{198}F_6N_{24}O_{12}PRuS_3Si_6^+$ ($[M-PF_6]^+$): 3759.21, found: 3759.76.

3. Supplementary Figures

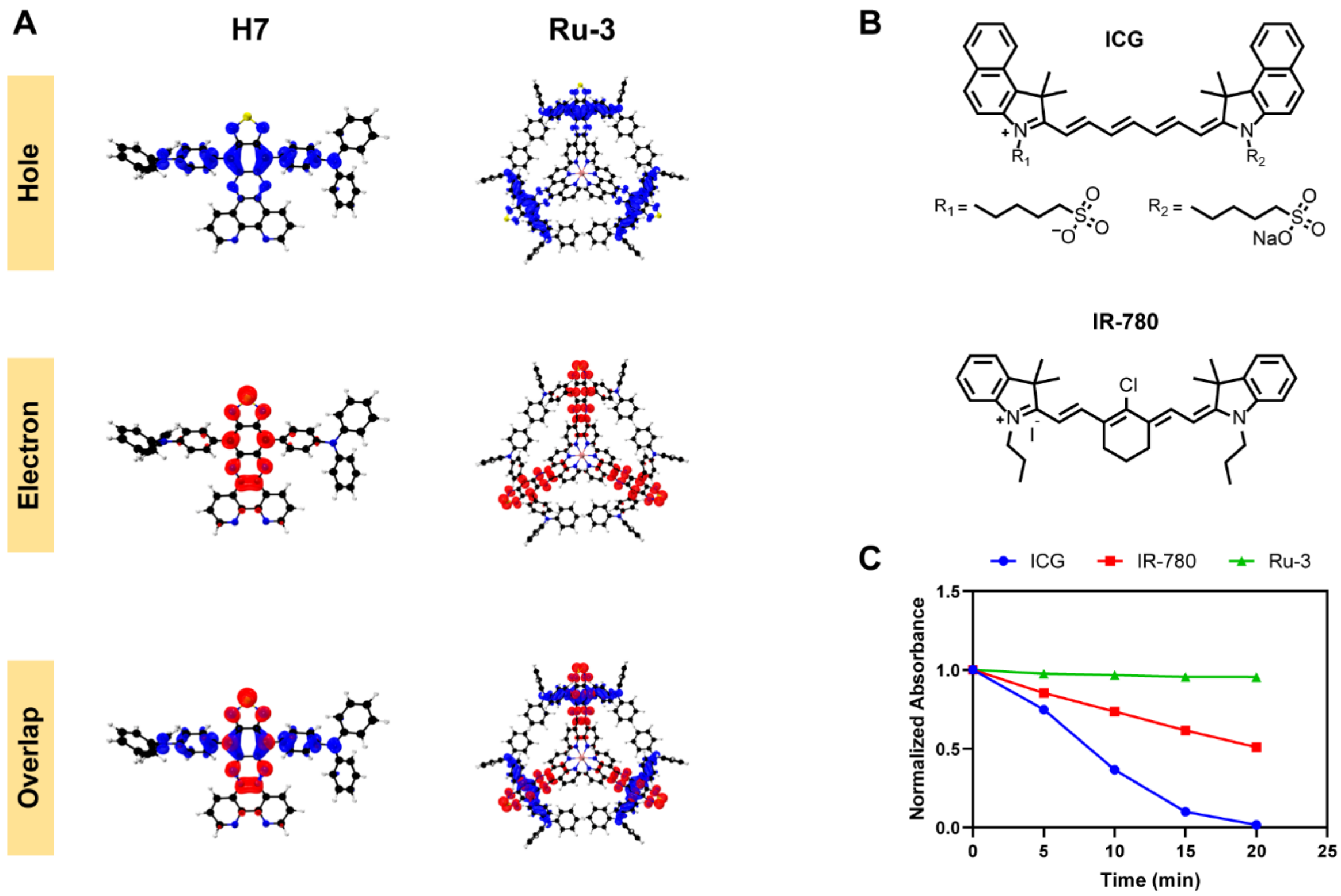

Fig. S8. Excited-state charge separation and photostability of Ru-3. (A) Electron-hole analysis of the vertical excitations of **H7** and **Ru-3**. **(B)** The chemical structures of IR-780 and ICG. **(C)** The photostability of **Ru-3**, IR-780, and ICG under continuous 808 nm irradiation (1.0 W·cm^{-2}), with fluorescence intensities at predetermined time points (0, 5, 10, and 20 min).

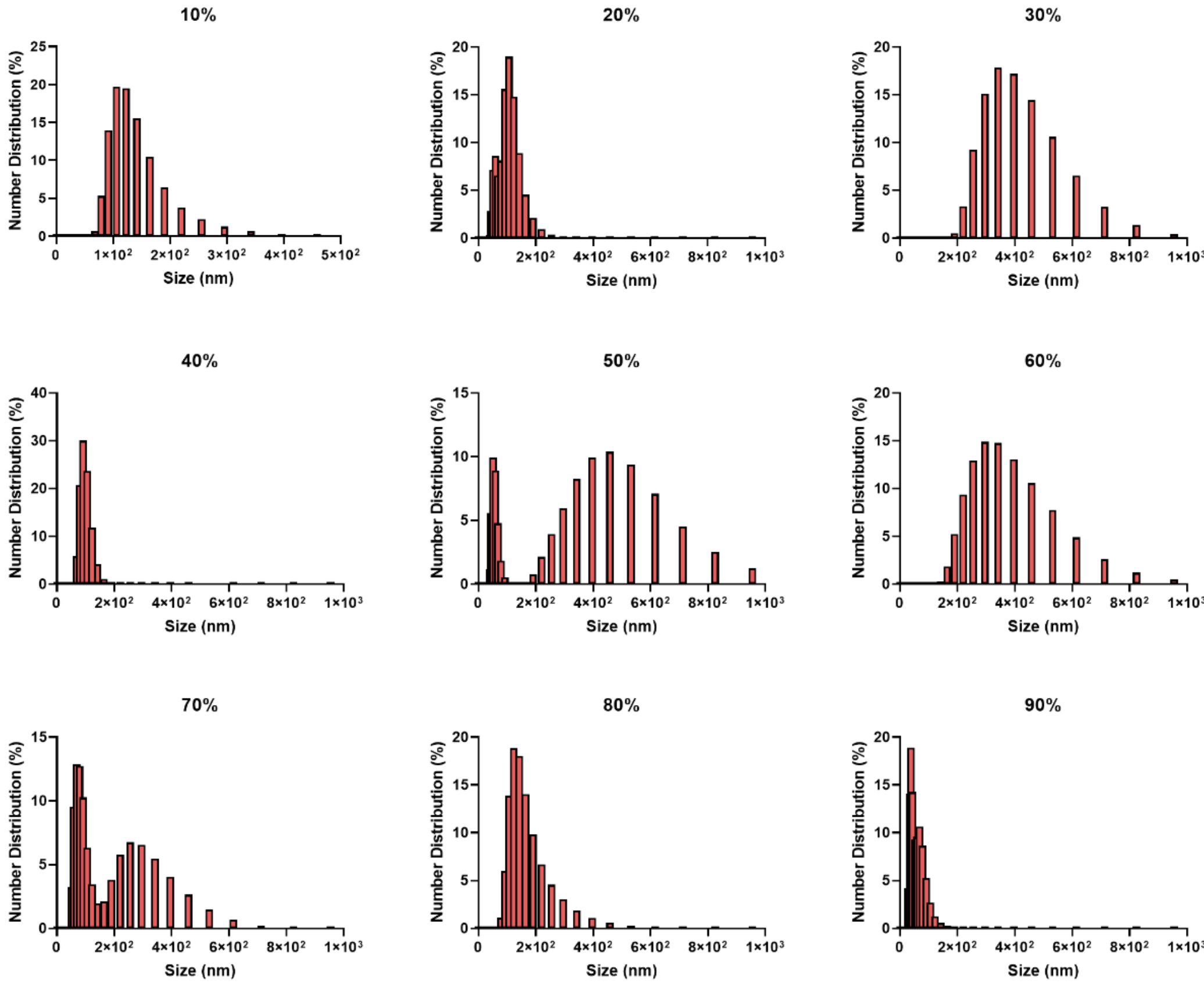

Fig. S9. The DLS of Ru-3 aggregates obtained from varied H_2O/DMSO ratios. No signal was observed when **Ru-3** was dissolved in pure DMSO, indicating the well dispersion of **Ru-3** molecules.

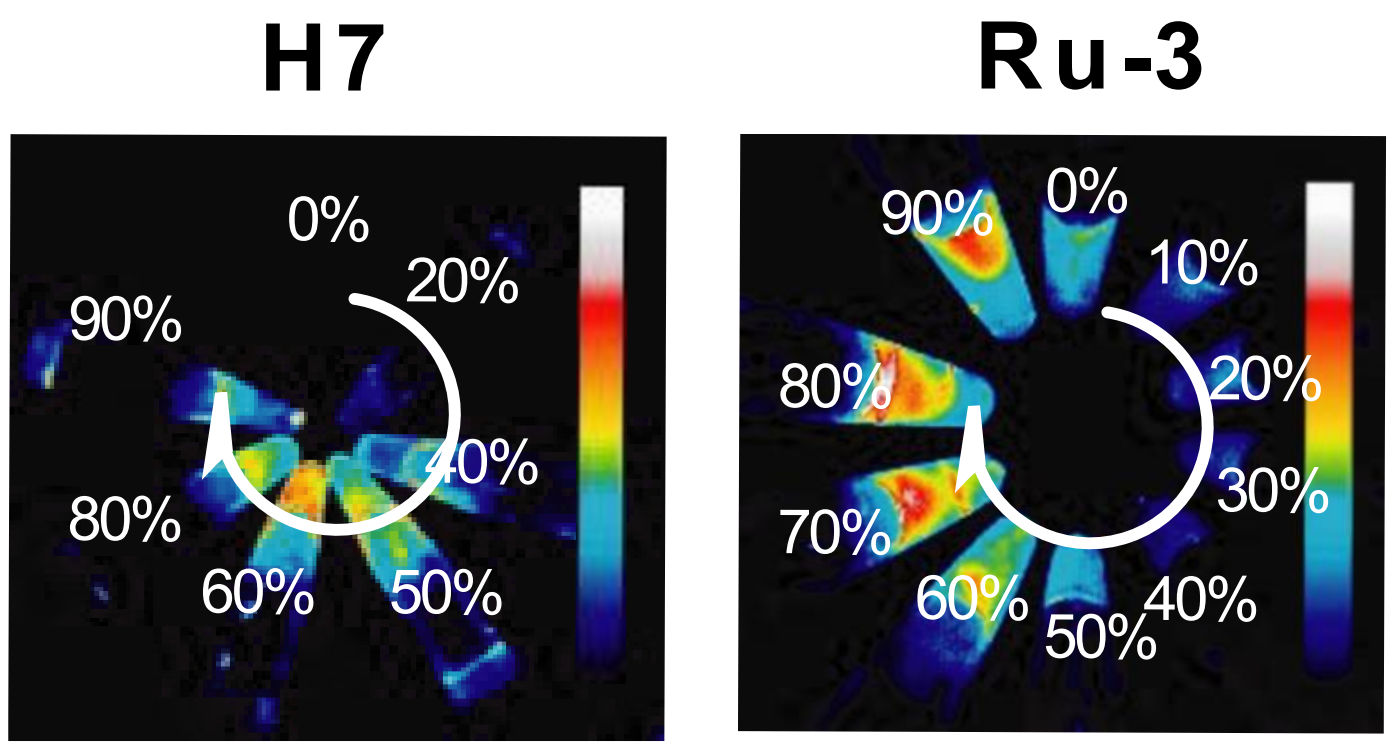

Fig. S10. NIR-II fluorescence images of H7 and Ru-3 in H_2O/DMSO solutions. Images were captured using a 1000 nm long-pass filter on an NIR-II imaging system (exposure time: 100 ms) with varying water fractions.

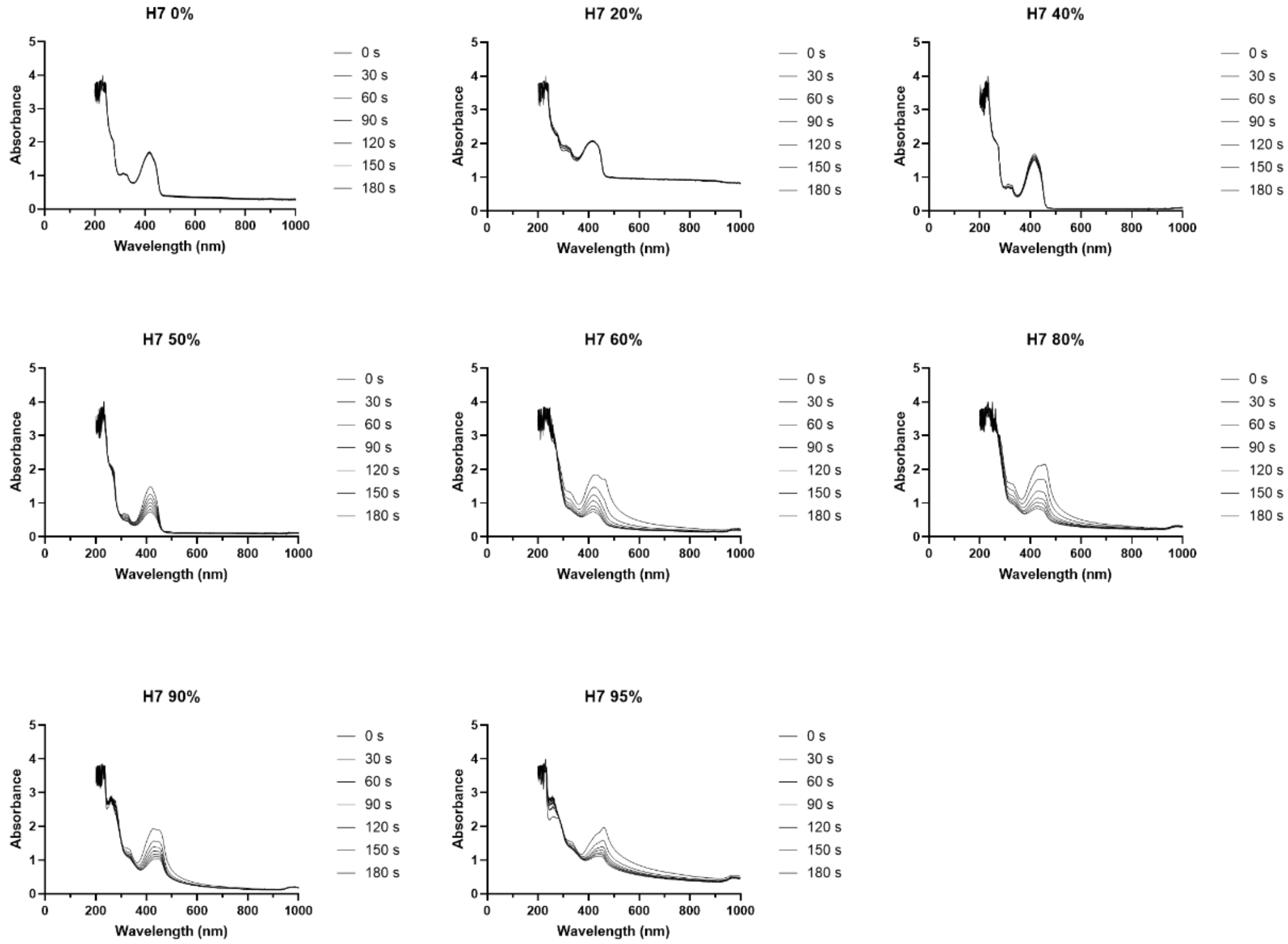

Fig. S11. Absorption spectrum of the DPBF and H7 mixture in H_2O/DMSO solution. This mixture underwent 808 nm irradiation at a power intensity of 0.5 W·cm^{-2} intermittently for 30 s, with the absorption spectrum recorded between each irradiation step until a cumulative irradiation time of 180 s was achieved.

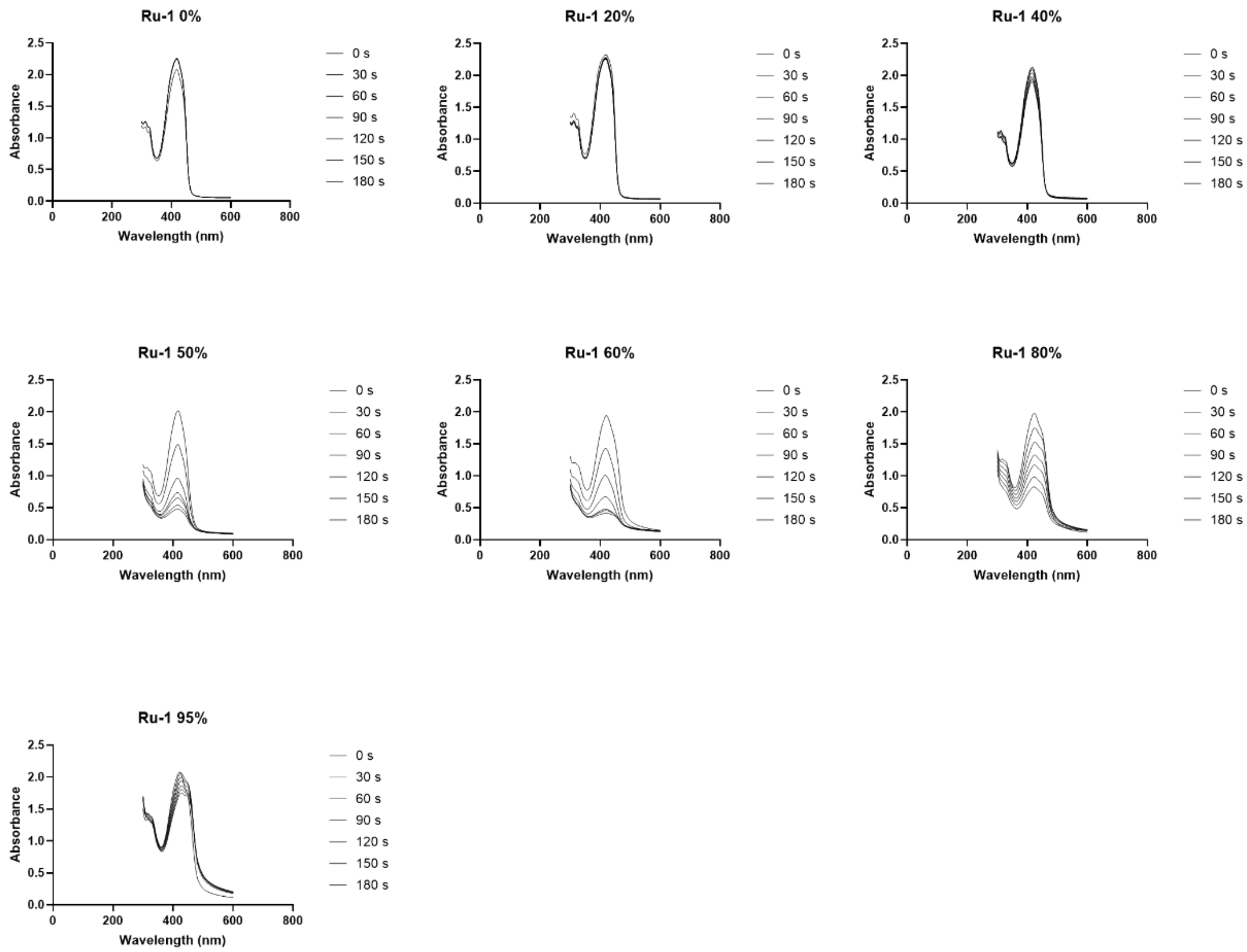

Fig. S12. Absorption spectrum of the DPBF and Ru-1 mixture in H_2O/DMSO solution. This mixture underwent 808 nm irradiation at a power intensity of 0.5 W·cm^{-2} intermittently for 30 s, with the absorption spectrum recorded between each irradiation step until a cumulative irradiation time of 180 s was achieved.

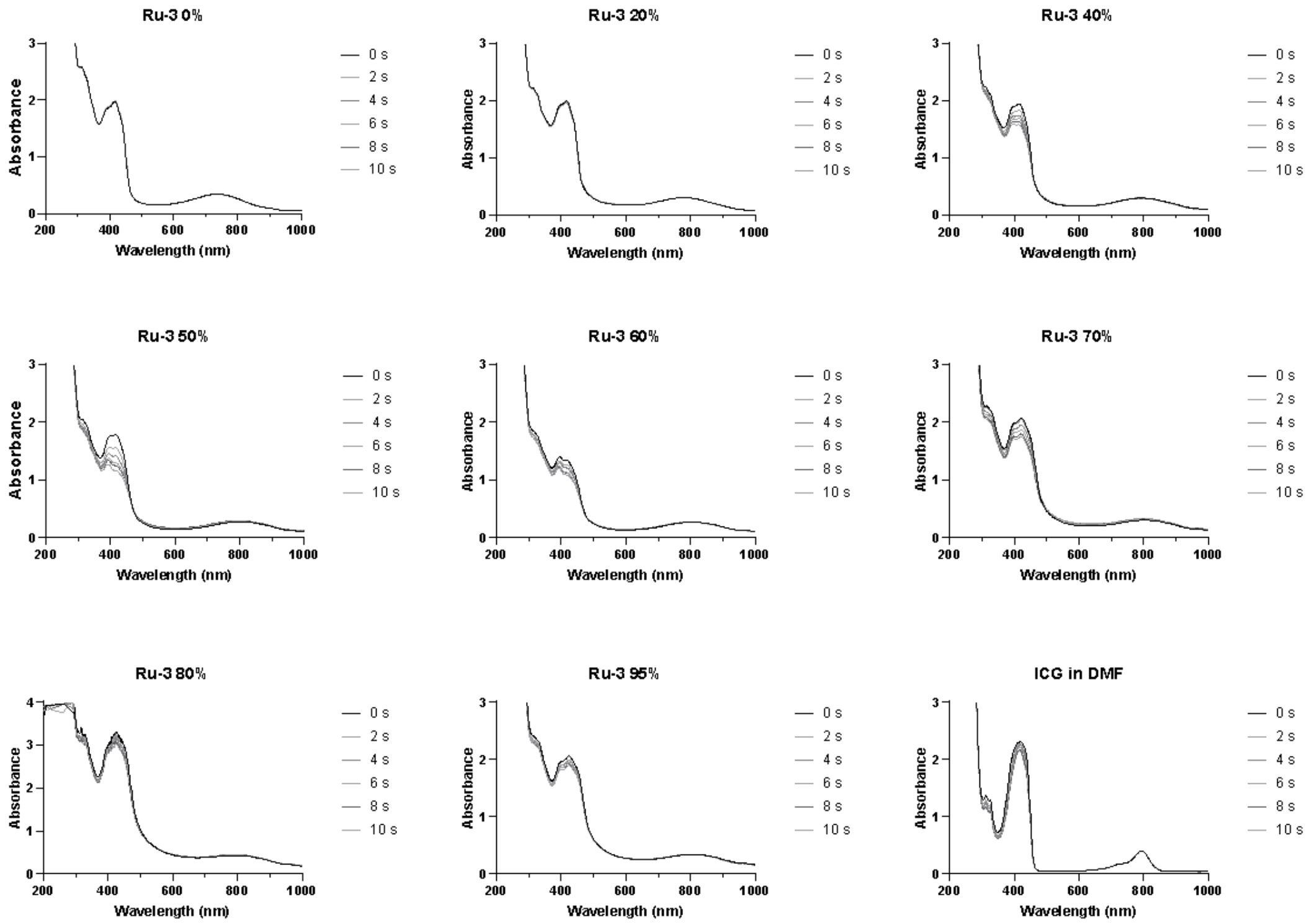

Fig. S13. Absorption spectra of the DPBF and Ru-3 mixture in H_2O/DMSO solution. This mixture underwent 808 nm irradiation at a power intensity of 0.1 W·cm^{-2} intermittently for 2 s, with the absorption spectrum recorded between each irradiation step until a cumulative irradiation time of 10 s was achieved.

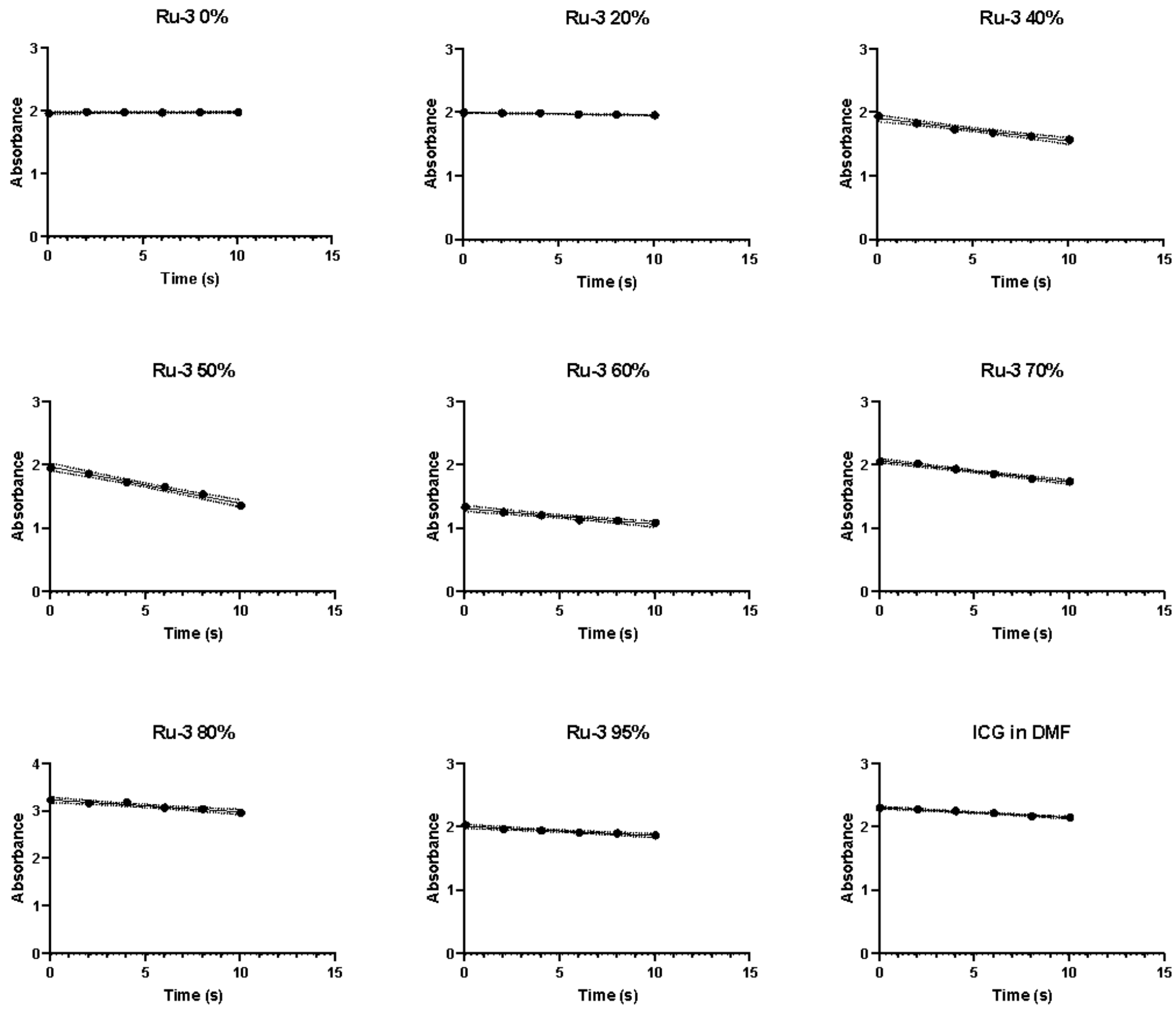

Fig. S14. Absorbance at 415 nm obtained from the absorption spectrum in Figure S13. Liner fitting was conducted by GraphPad 8.0.1. The slope indicates the degradation rate of the DPBF upon 808 nm irradiation (0.1 W cm^{-2}).

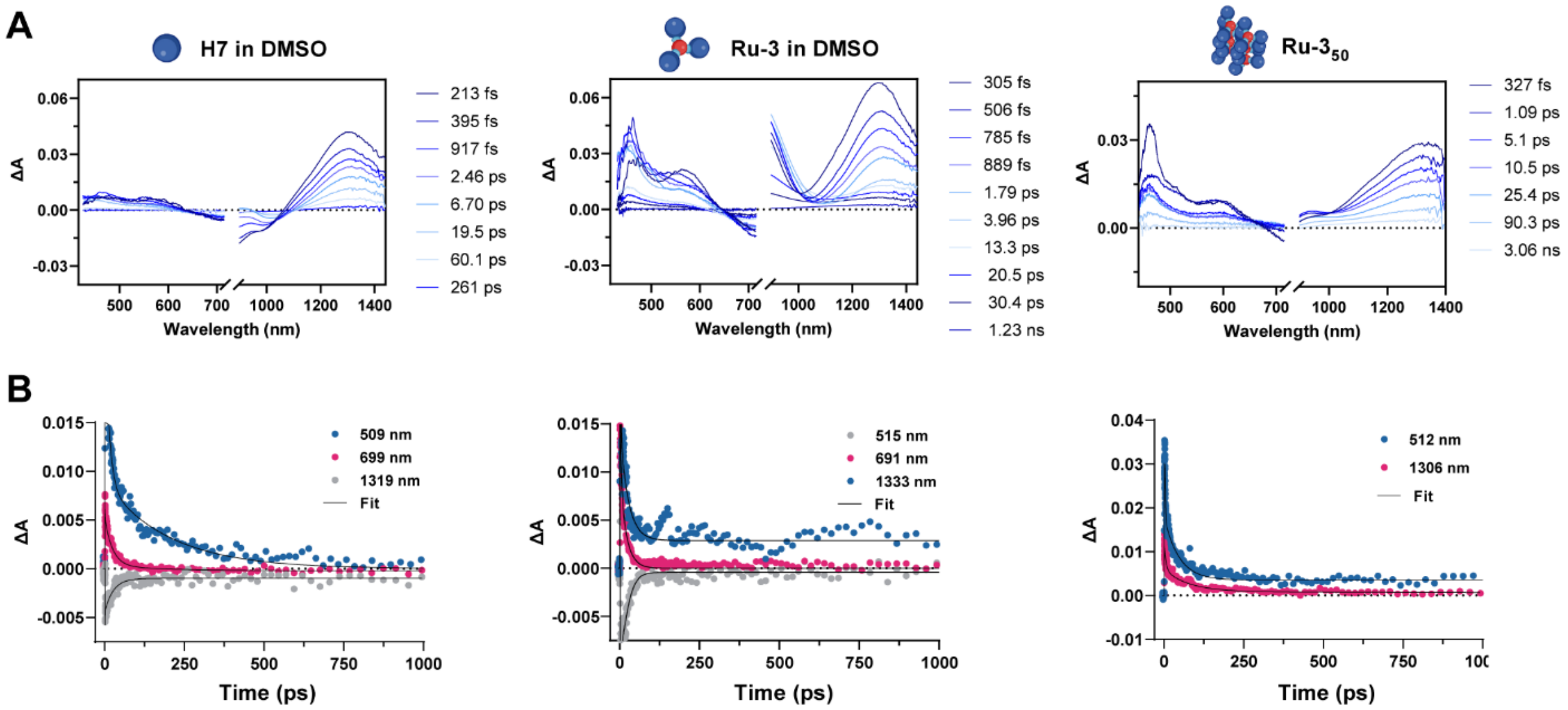

Fig. S15. fs-TA reveals aggregation-dependent excited-state evolution and long-lived triplet kinetics. (A) Transient absorption spectra were recorded for **H7** in DMSO, **Ru-3** in DMSO, and **Ru-3$_{50}$** at various pump-probe time delays. fs-TA spectra of **H7** aggregates could not be obtained, likely due to significant laser scattering caused by tight aggregation. (B) Decay kinetic traces of these compounds at selected wavelengths were derived from global analysis of the fs-TA spectra.

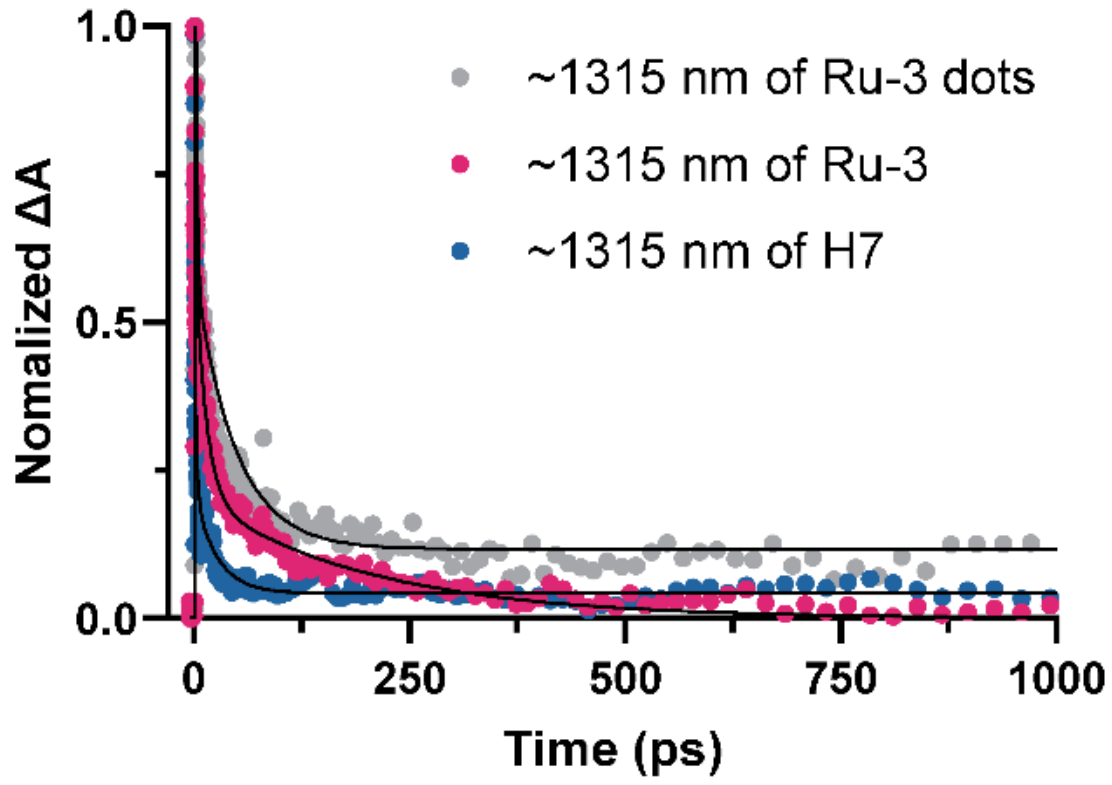

Fig. S16. The normalized decay kinetic traces of H7 in DMSO, Ru-3 in DMSO, and Ru-3 aggregates at ~1315 nm.

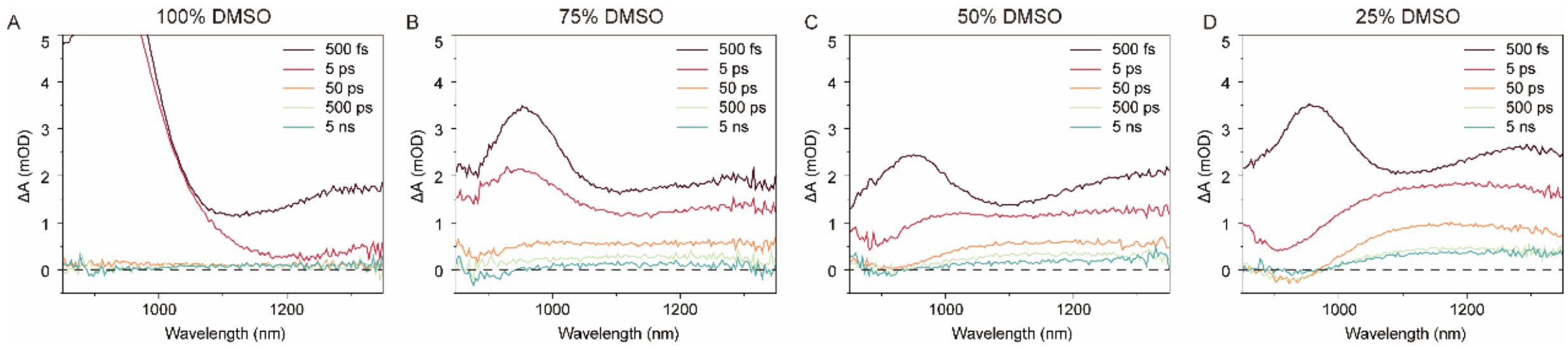

Fig. S17. **Extracted fs-TA plots of Ru-3 in H_2O/DMSO solution.** Extracted fs-TA plots of **Ru-3** in different DMSO fraction under different delay time pump at 800 nm. As the DMSO fraction decreases, the ΔA intensities at 1315 nm are 1.67, 1.72, 2.13, and 2.61 mOD, respectively.

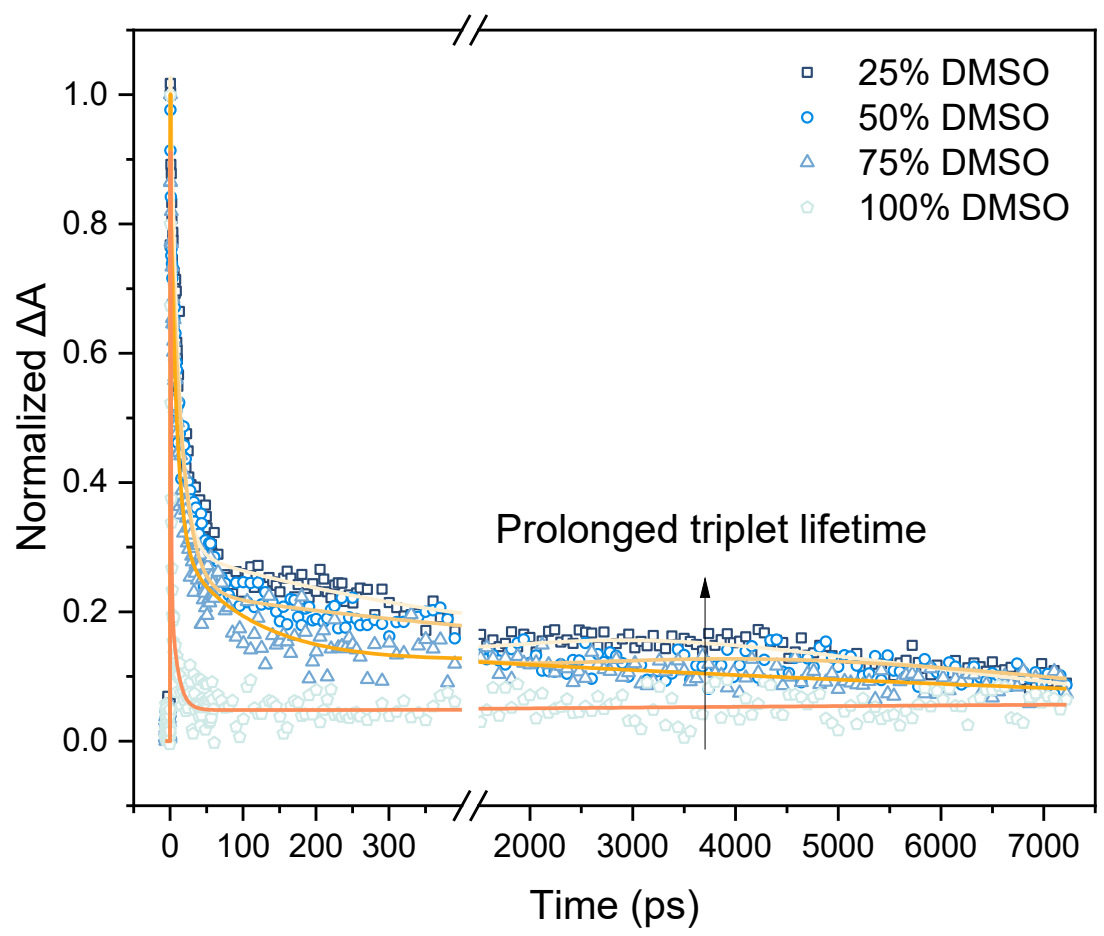

Fig. S18. **Kinetics traces for Ru-3 at the representative wavelength within triplet (1315 nm) ESA region.**

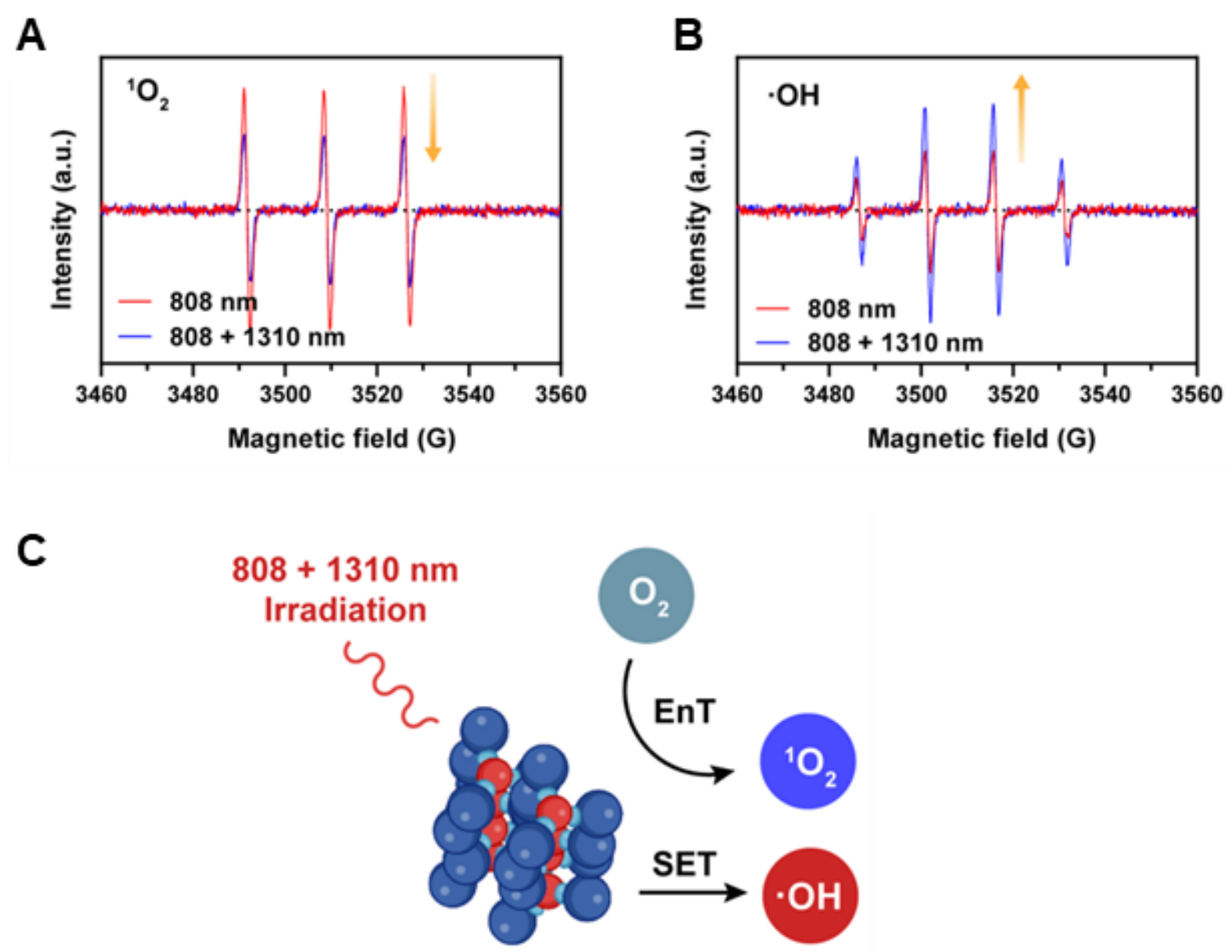

Fig. S19. ESR evidence of PACE-enhanced radical generation under dual NIR excitation. (**A** and **B**) ESR spectra of **Ru-3** dots in aqueous solution were obtained after 3 minutes of irradiation with either an 808 nm laser or consecutive 808 nm and 1310 nm laser irradiation. DMPO and TEMP were used as spin-trapping agents to detect singlet oxygen (A) and radicals (B), respectively. (**C**) Schematic illustrating the generation of radicals by **Ru-3** dots under consecutive NIR I/IIa photon excitation.

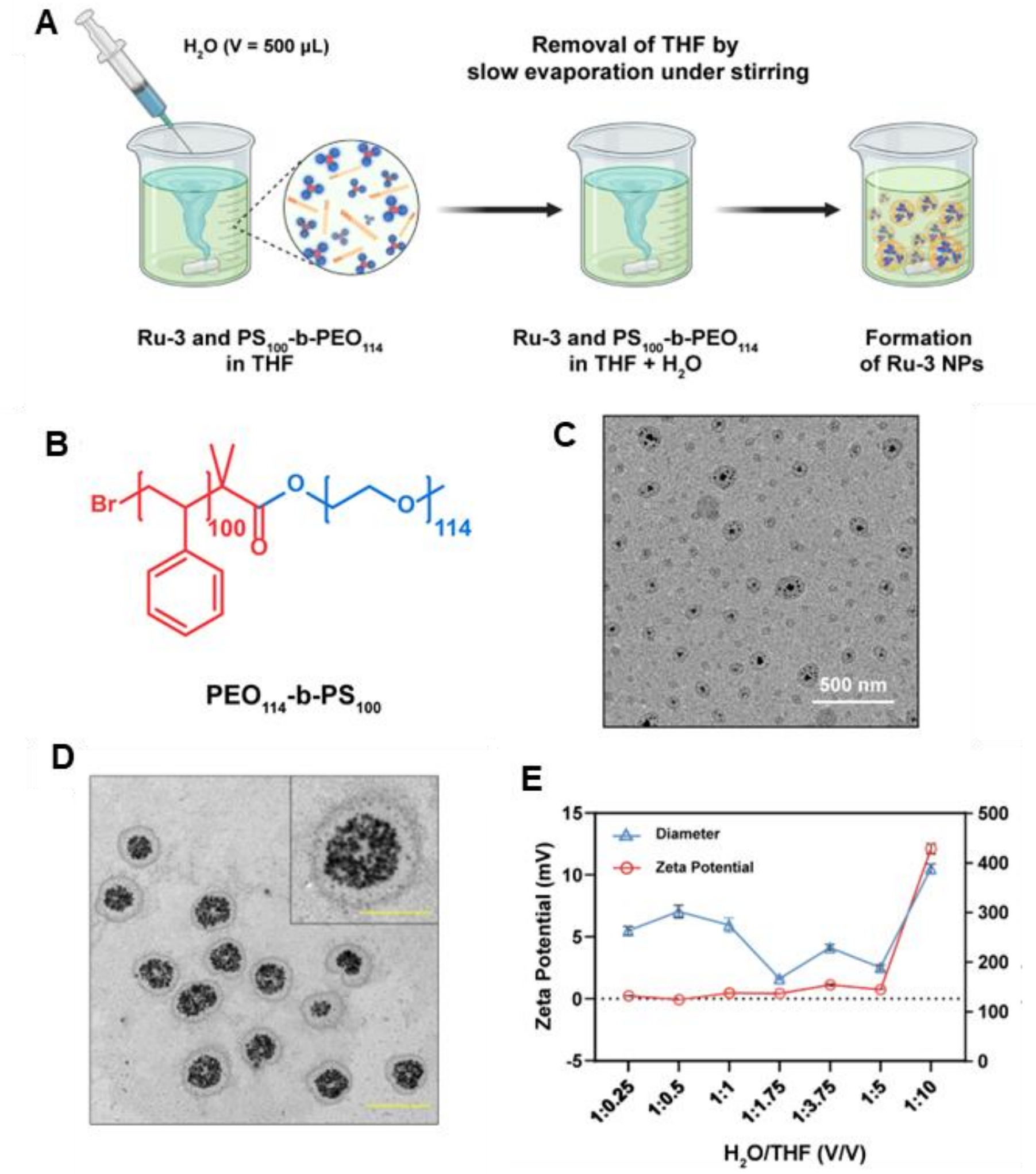

Fig. S20. Preparation and characterization of Ru-3 nanoparticles. (A) Schematic illustration of the formation and *in-situ* encapsulation of **Ru-3 NPs** within PEO_{114}-b-PS_{100} using solvent-switch method. Created in BioRender. lou, Z. (2026) https://BioRender.com/1kngn91 **(B)** The chemical structure of PEO_{114}-b-PS_{100}. **(C)** A representative TEM image of **Ru-3 NPs** in a H_2O/THF mixture at an initial volume ratio of 1:1.75 (scale bar: 500 nm). (**D**) Representative TEM image of **Ru-3 NPs** obtained from a 1:10 initial H_2O/THF ratio (scale bar: 2 μm). (**E**) The DLS and Zeta potential of **Ru-3 NPs** obtained from varying initial H_2O/THF ratios.

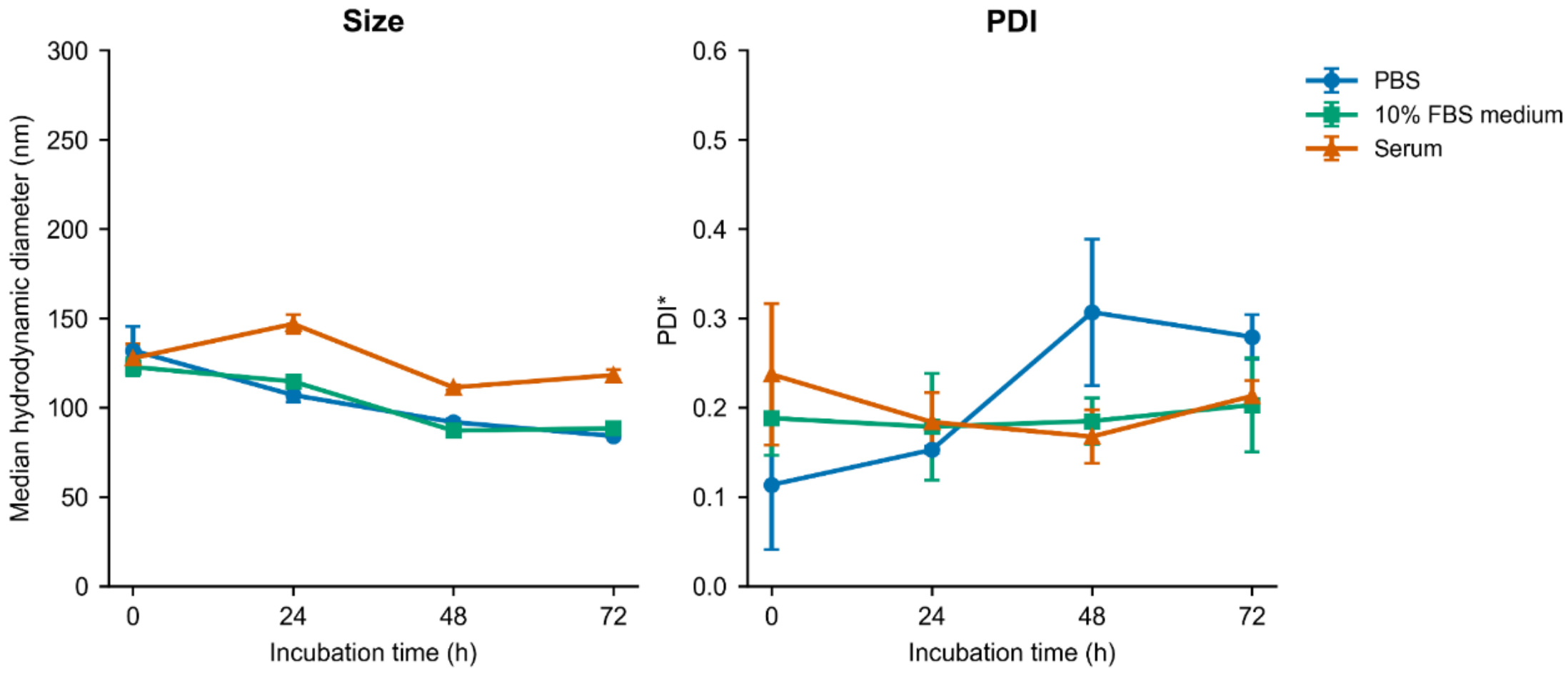

Fig. S21. Colloidal stability of Ru-3 NPs in different physiological media. Ru-3 NPs were dispersed in PBS, cell culture medium containing 10% FBS, or serum and incubated for the indicated time points under 37°C conditions. Data are shown as mean +/- SD from three technical readings. **Ru-3** nanoparticles retained nanoscale dimensions in PBS, 10% FBS-containing medium, and serum over the tested incubation period, without evidence of large aggregate formation.

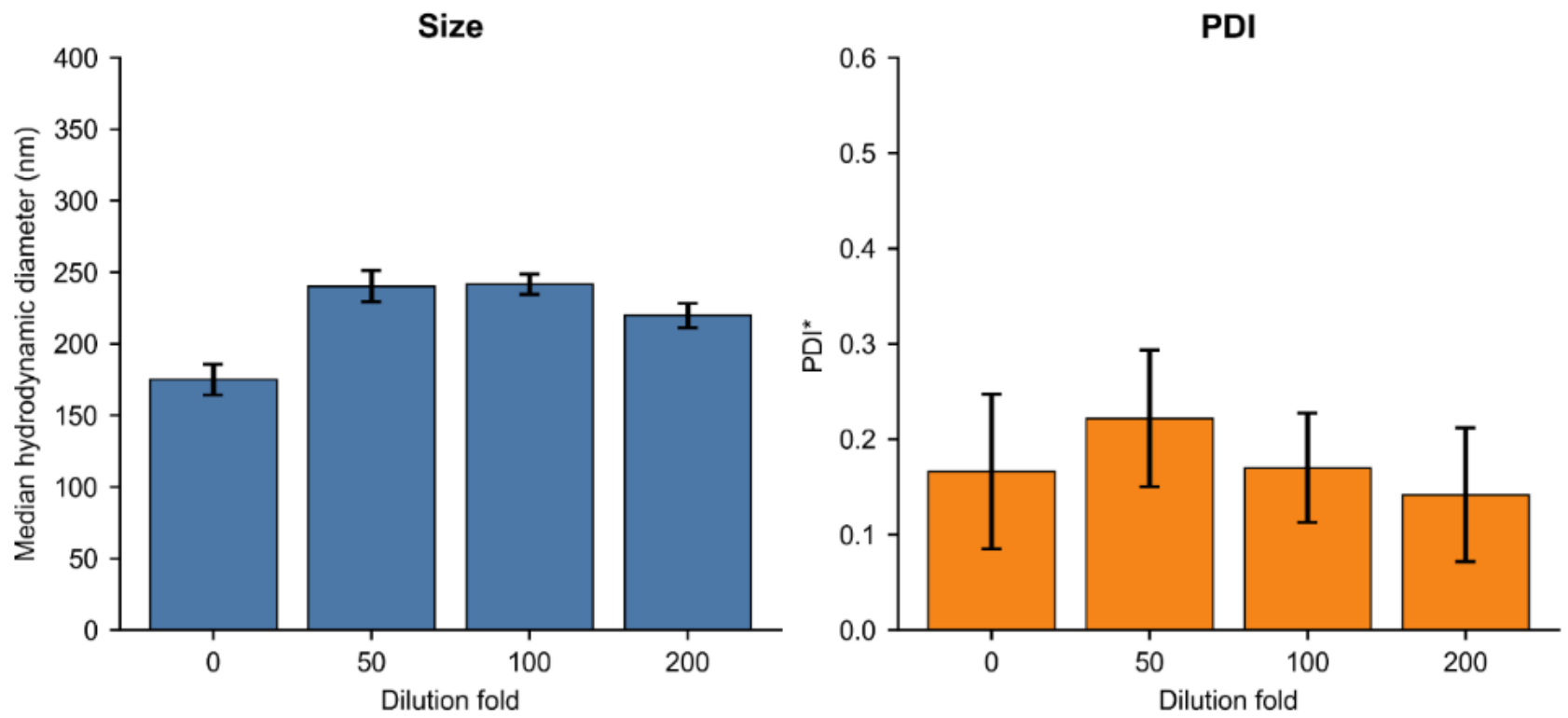

Fig. S22. Dilution stability of Ru-3 nanoparticles. Ru-3 NPs stock dispersion was diluted by the indicated fold changes in PBS. Data are presented as mean +/- SD from three technical readings. The median hydrodynamic diameters remained within the nanoscale range after dilution, supporting that the assembled **Ru-3** nanoparticles remain colloidally stable under low-concentration conditions relevant to systemic delivery.

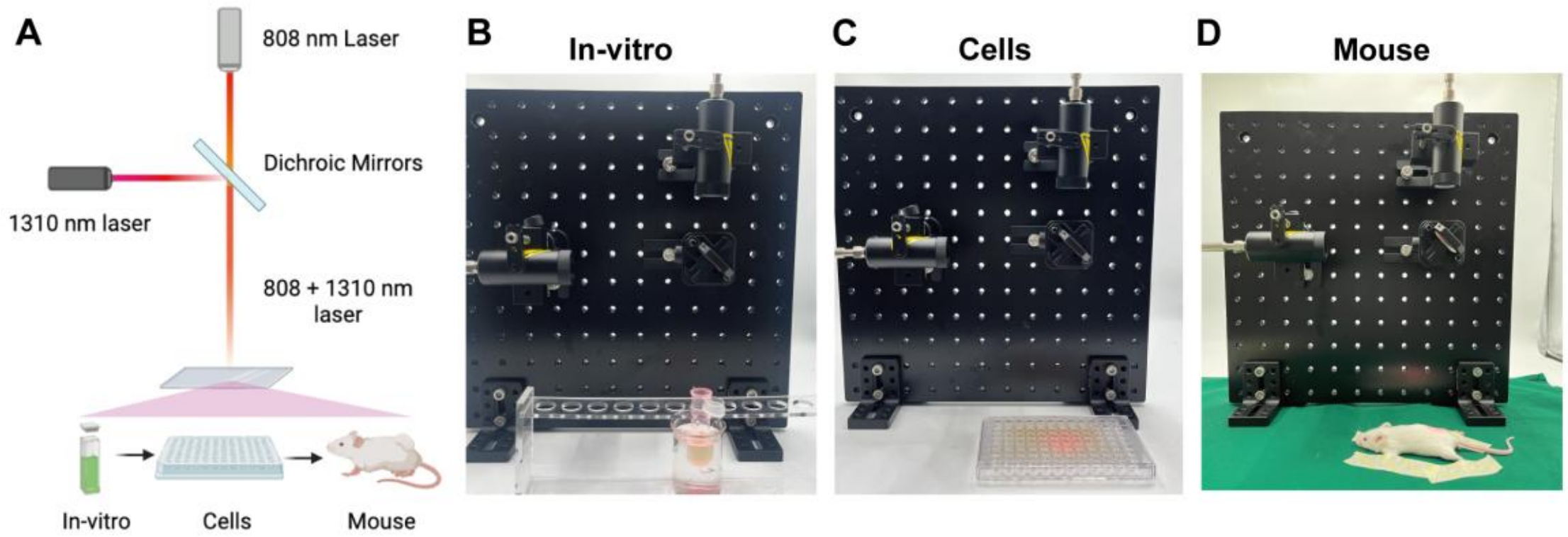

Fig. S23. Dual-wavelength optical irradiation platform. (A) Schematic illustration of the optical platform configured with dichroic mirrors for combined 808 and 1310 nm laser irradiation. Created in BioRender. R, YU. (2026) https://BioRender.com/9f63dex. (B) Representative optical image of an NADH/Ru-3 sample under simultaneous 808 and 1310 nm irradiation. (C) Representative optical image of a 4T1 cells in 96-well plate exposed to simultaneous 808 and 1310 nm irradiation. (D) Representative optical image of a BALB/c mouse exposed to simultaneous 808 and 1310 nm irradiation.

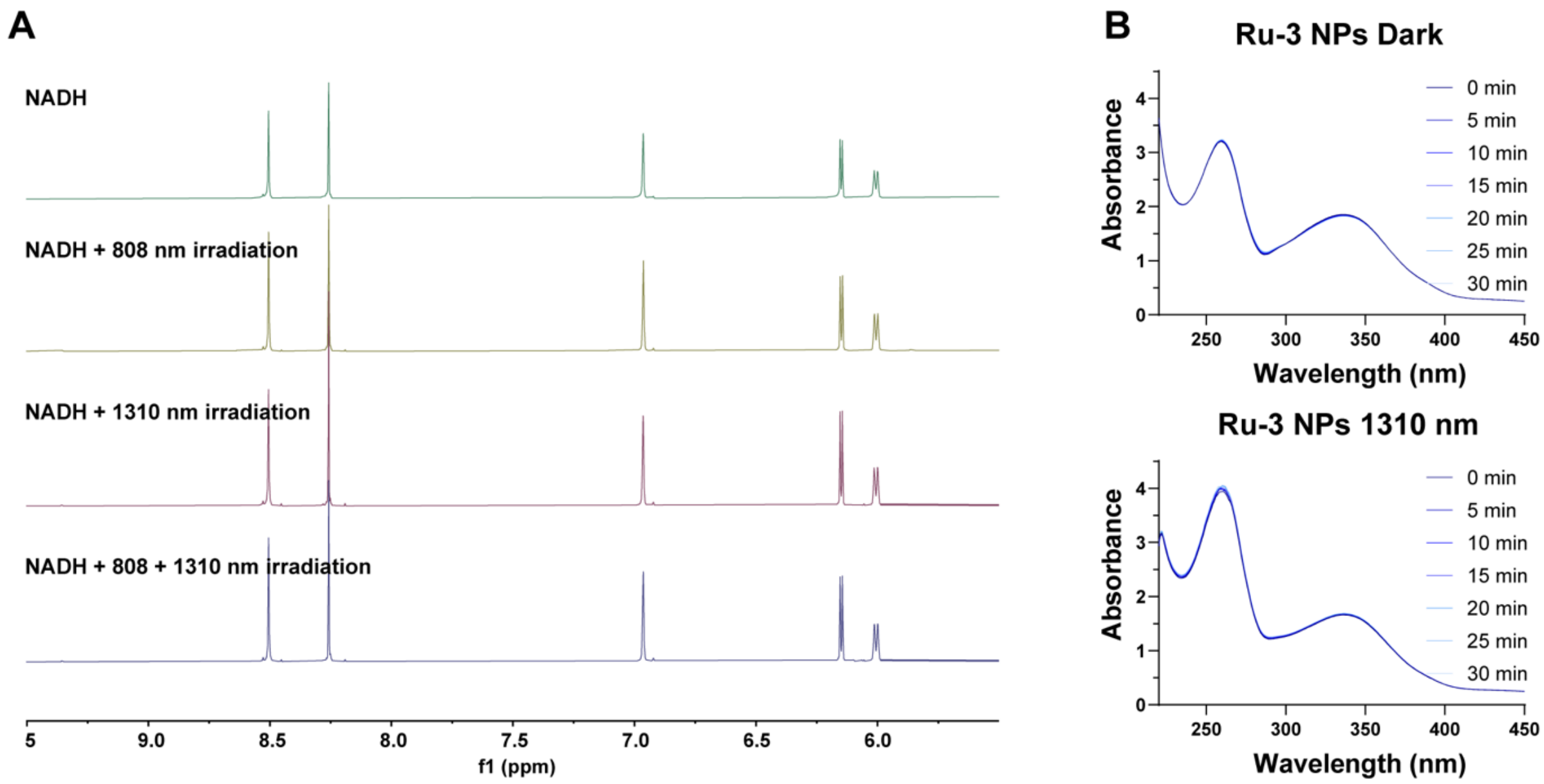

Fig. S24. NMR and UV–vis analysis of NADH under different irradiation conditions. (A) ^{1}H NMR spectra of NADH (3 mM) in the dark, 808 nm, 1310 nm, 808 + 1310 nm irradiation for 20 min. Water bath was carried out to exclude the influence of thermal effect of laser irradiation. **(B)** UV-vis spectra indicate the photooxidation of NADH (120 μM) under dark conditions or upon 1310 nm irradiation in the presence of **Ru-3 NPs** (10 μM). The laser power densities were 0.5 $W\cdot cm^{-2}$ for 808 nm and 1.0 $W\cdot cm^{-2}$ for 1310 nm.

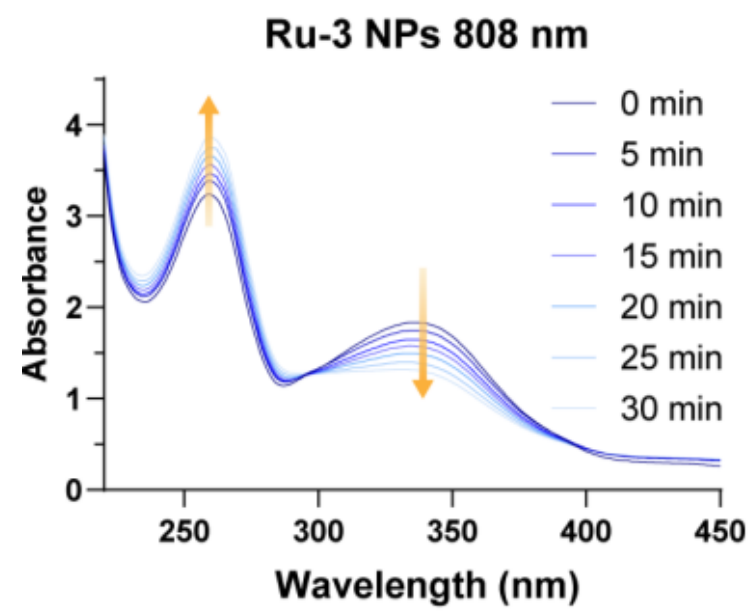

Fig. S25. Photooxidation of NADH in the presence of Ru-3 NPs. UV-vis spectra showing the photooxidation of NADH (180 µM) under 808 nm irradiation (0.5 W cm^{-2}) in the presence of **Ru-3 NPs** (10 µM) during 30 minutes.

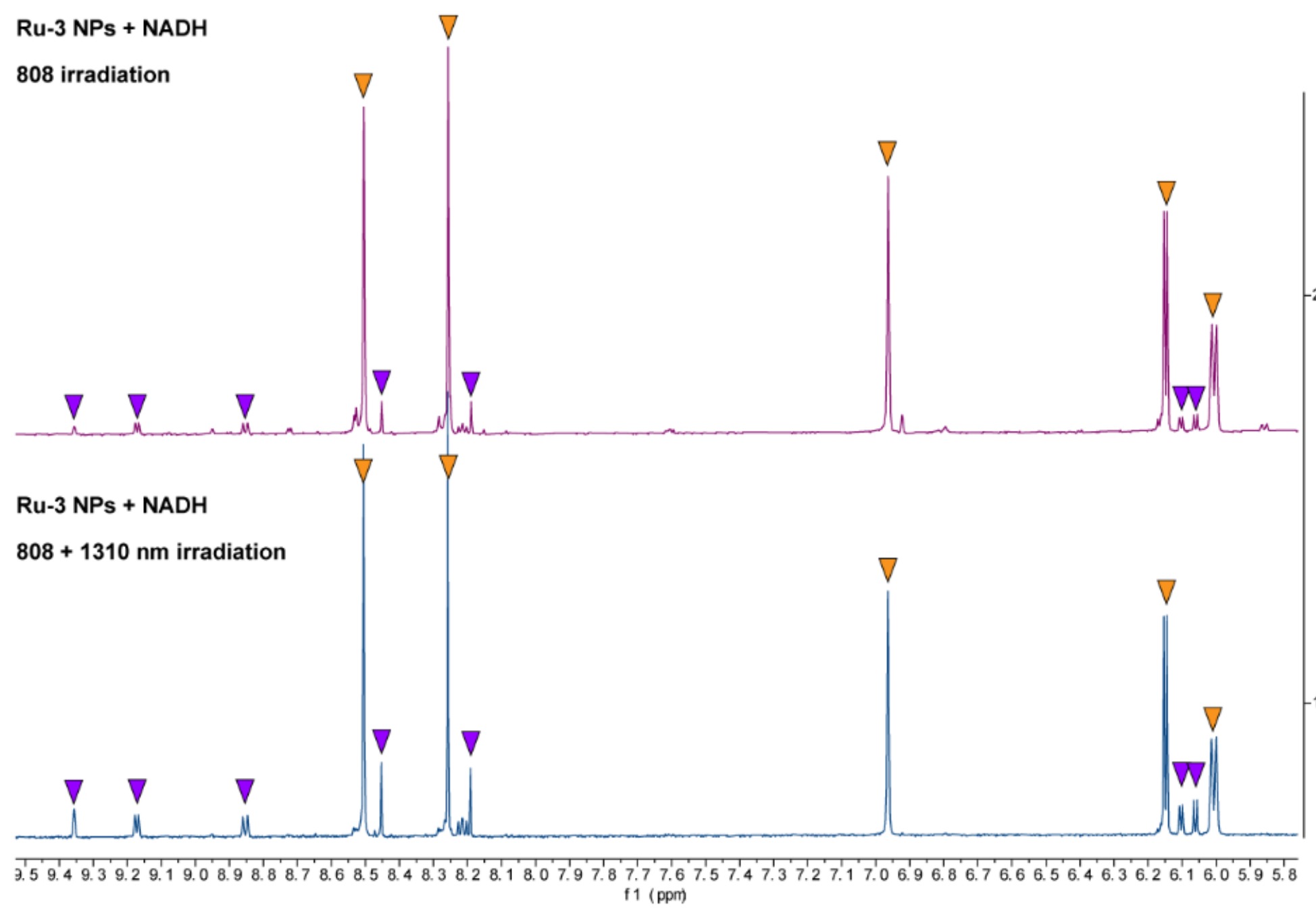

Fig. S26. NMR spectrum of NADH in the present of Ru-3 NPs. ^{1}H NMR spectrum of NADH (3 mM) in the present of **Ru-3 NPs** (0.1 mM) upon 808 nm or 808 + 1310 nm irradiation for 20 min. Water bath was carried out to exclude the influence of thermal effect of laser irradiation. The power intensity of 808 and 1310 nm laser was 0.5 W·cm^{-2} and 1.0 W·cm^{-2}, respectively.

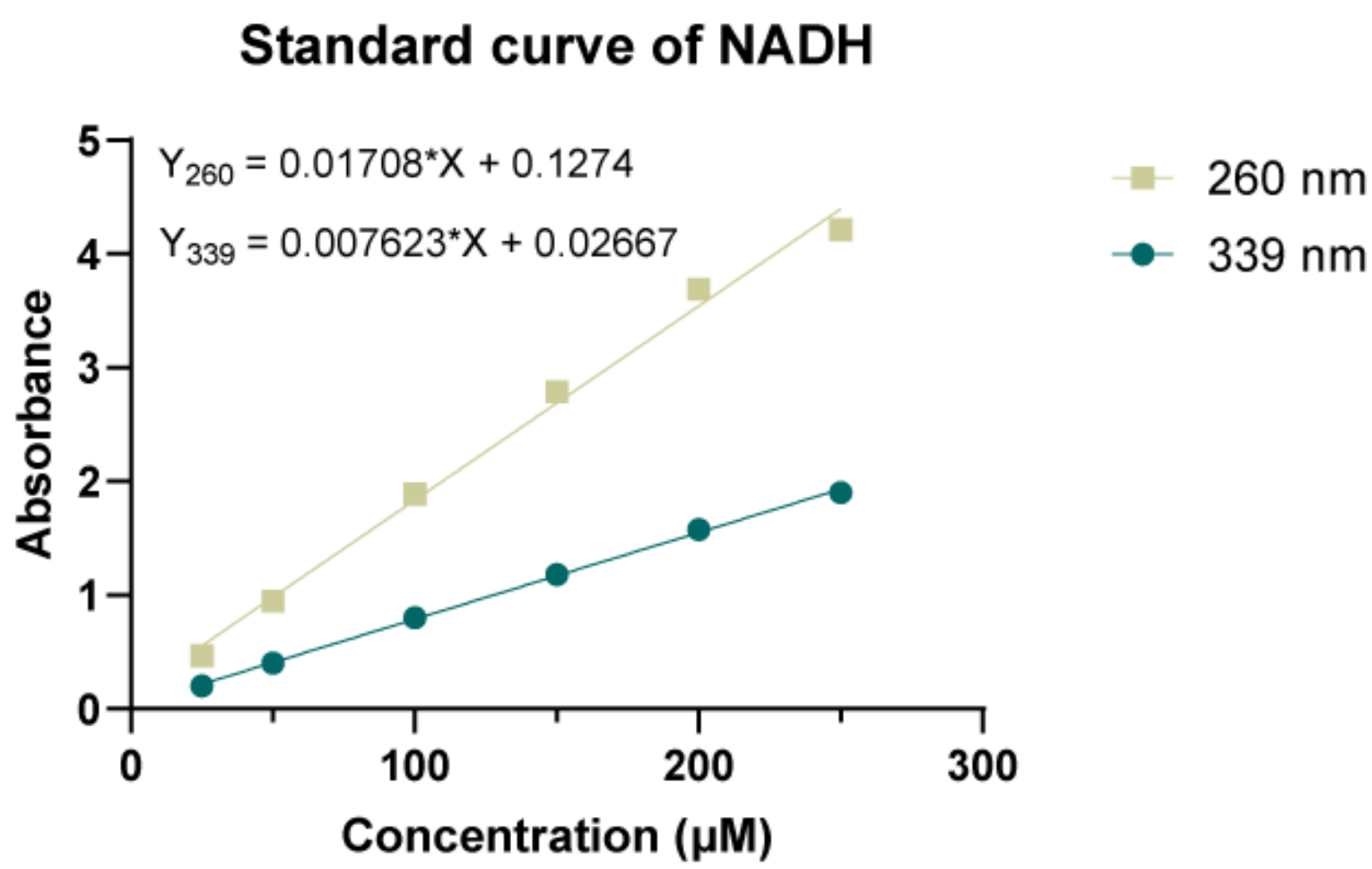

Fig. S27. Standard curve of NADH. UV-Vis spectrum of NADH in water with a series of concentrations from 25 μM to 250 μM. The corresponding standard linear fitting plot was presented on the right side.

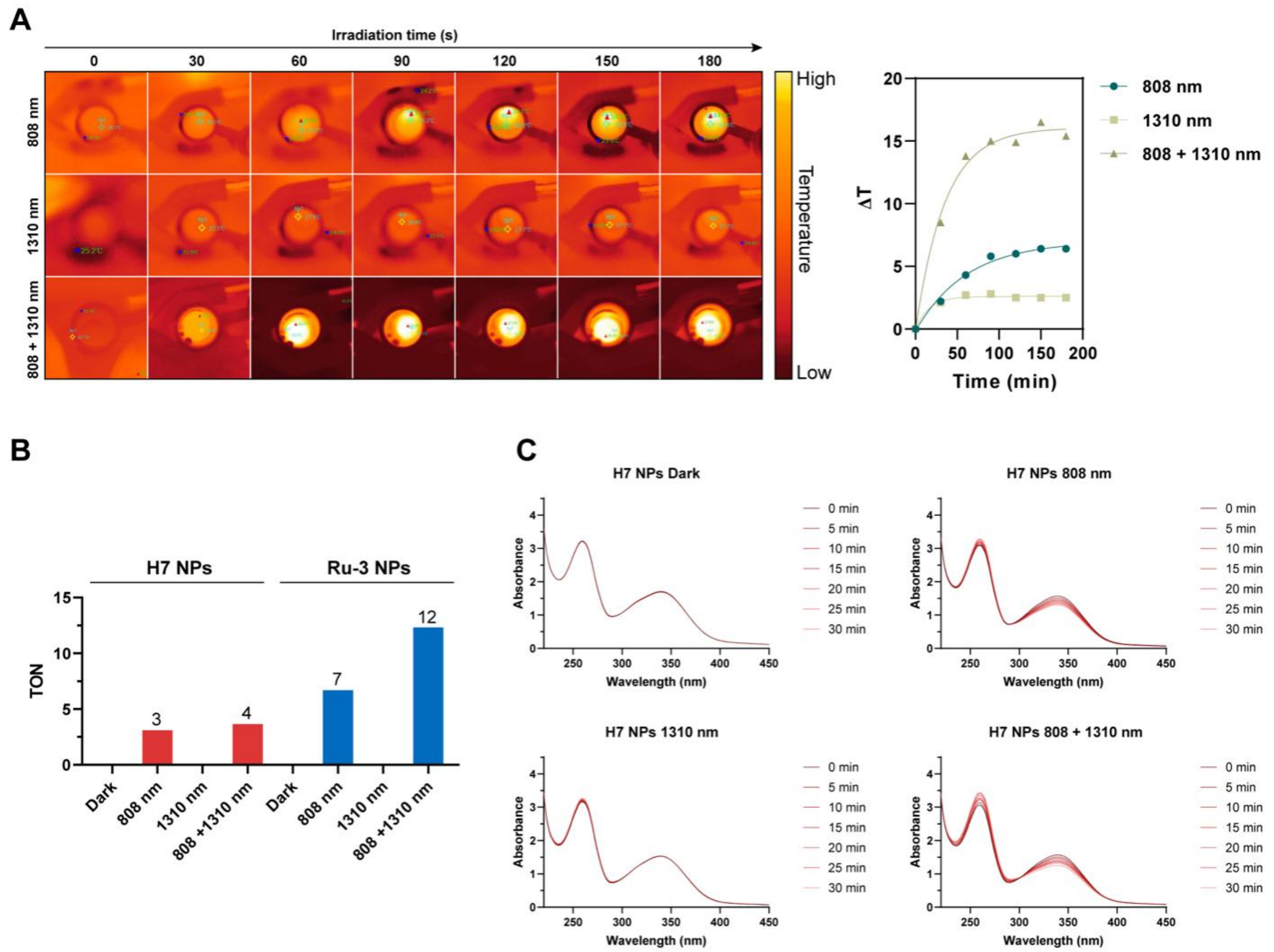

Fig. S28. Photothermal control and enhanced photocatalytic performance of Ru-3 under PACE conditions. (A) Infrared thermal images and corresponding heating curves were obtained for a PBS solution containing **Ru-3 NPs** (10 μM) and NADH (180 μM) under 808 nm, 1310 nm, or combined 808 + 1310 nm irradiation. A water bath was employed to mitigate the thermal effects of laser irradiation. The laser power densities were 0.5 W·cm⁻² for 808 nm and 1.0 W·cm⁻² for 1310 nm. **(B)** Turn over number of NADH for **H7 NPs** and **Ru-3 NPs** under varying irradiation conditions. **(C)** UV-vis spectra for the photocatalytic oxidation of NADH (180 μM) by **H7 NPs** (10 μM).

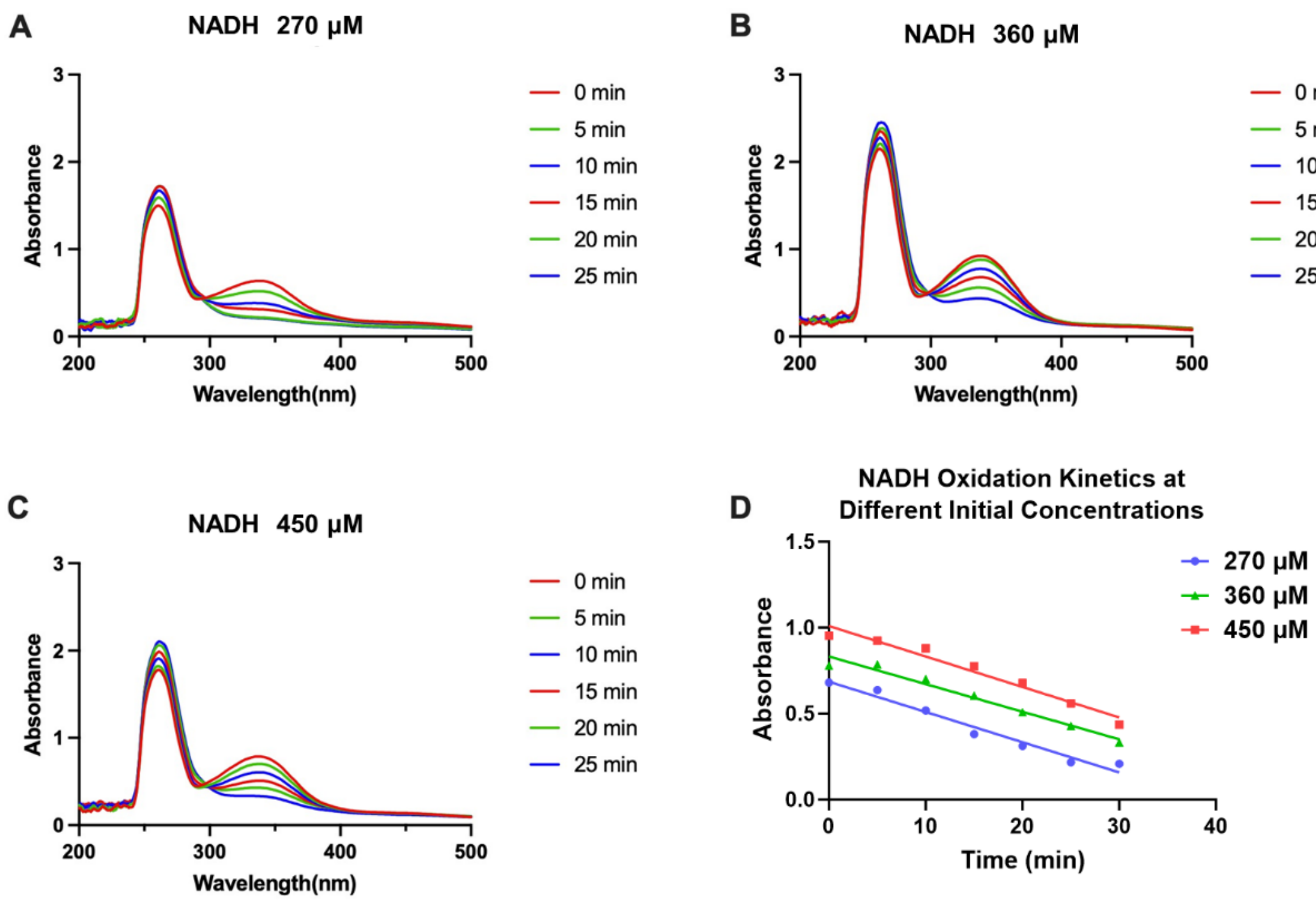

Fig. S29. Substrate concentration-dependent NADH oxidation kinetics under dual-wavelength irradiation. Time-dependent UV-vis absorption spectra of NADH oxidation at initial NADH concentrations of (A) 270 μM, (B) 360 μM, and (C) 450 μM under 808 + 1310 nm irradiation. Spectra were recorded every 5 min from 0 to 25 min. The gradual decrease of the NADH absorption band around 339 nm indicates progressive NADH oxidation during irradiation. (D) Linear regression analysis of the NADH absorbance at 339 nm as a function of irradiation time for the three initial NADH concentrations. The apparent NADH consumption rates obtained from the absolute slopes were 0.0177, 0.0161, and 0.0176 Abs min^{-1} for the 270, 360, and 450 μM groups, respectively. The corresponding turnover numbers (TONs) were calculated to be 22.1, 20.9, and 24.2 under 30 min irradiation

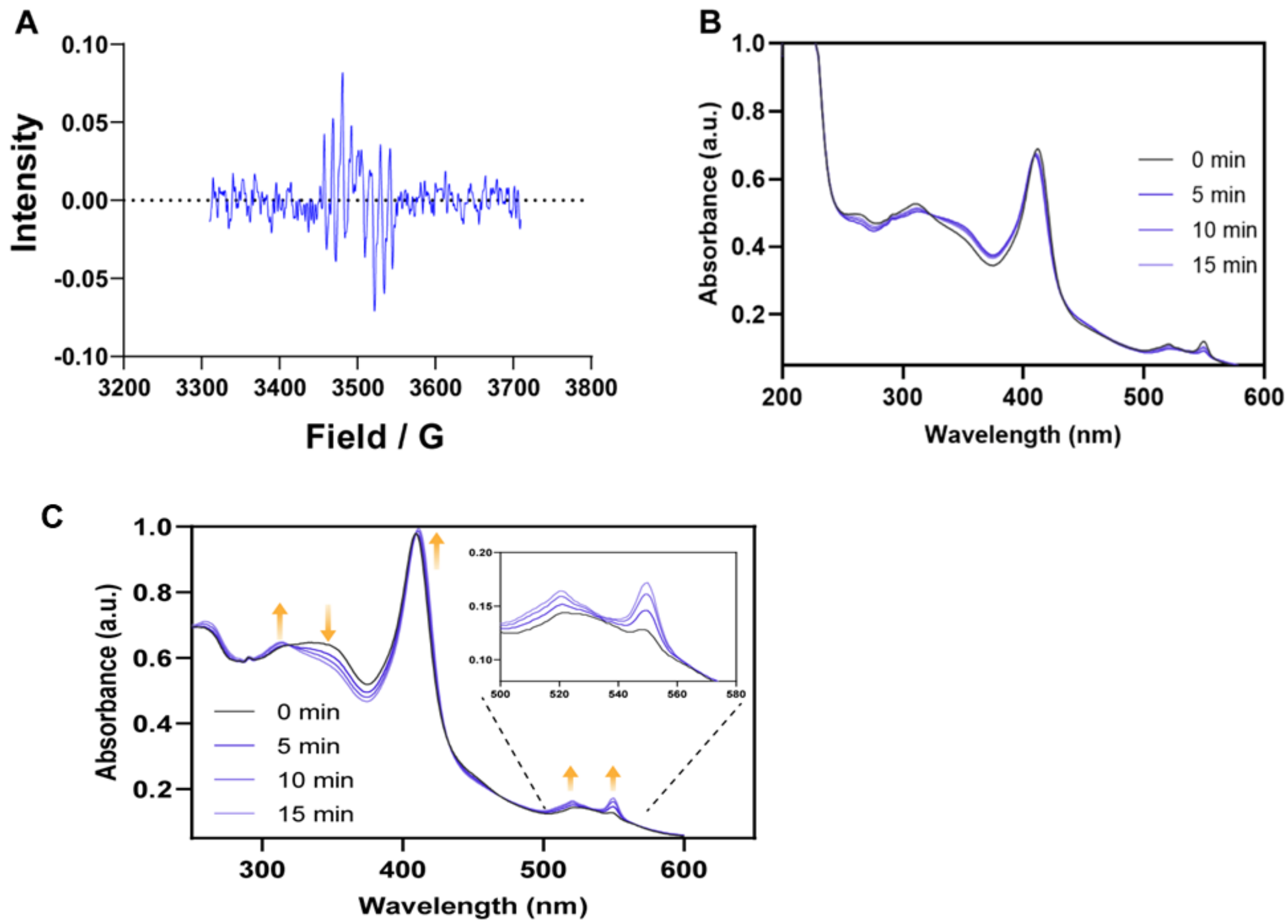

Fig. S30. EPR detection of NAD• radicals and photocatalytic cyt c reduction under NIR irradiation. (A) EPR spectra of NAD• radicals, trapped by CYPMPO after 10 minutes of irradiation with an 808 nm laser (1.0 W·cm⁻²). **(B)** Photocatalytic reduction of oxidized cyt c (8 μM) by NADH (30 μM) in the presence of **Ru-3 NPs** (0.5 μM) under 808 (0.5 W cm^{-2}) irradiation. (**C**) Photocatalytic reduction of oxidized cyt c (8 μM) by NADH (30 μM) in the presence of **Ru-3 NPs** (0.5 μM) under 808 (0.5 W cm^{-2}) + 1310 nm irradiation (1.0 W cm^{-2}) irradiation. Inset: magnified absorption spectra in the 500-580 nm range.

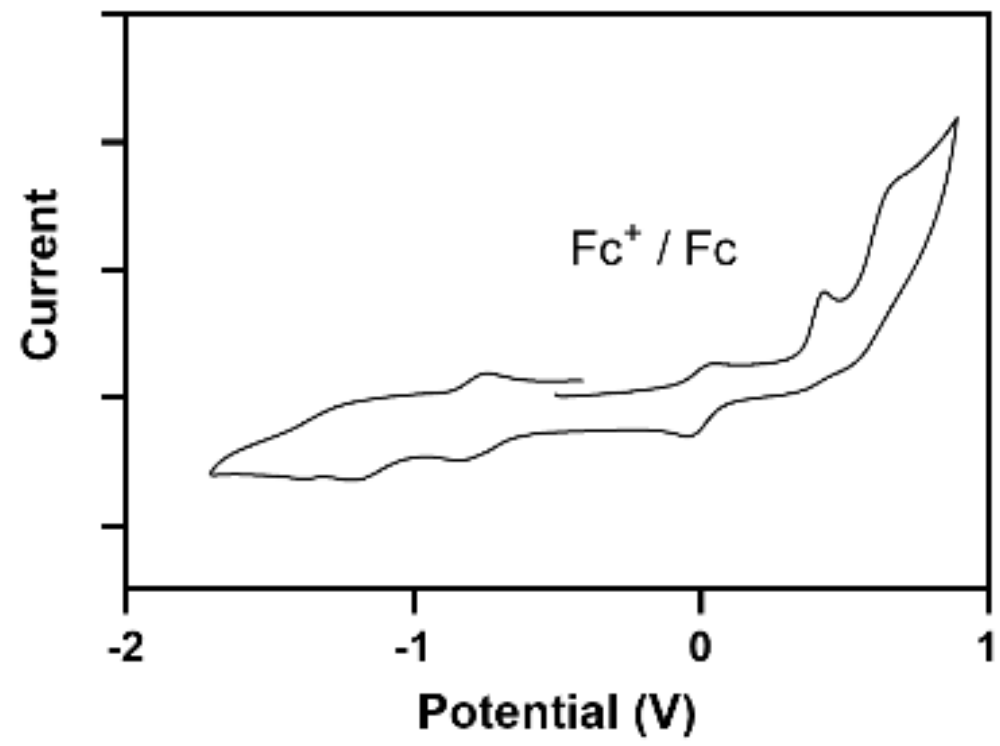

Fig. S31. Cyclic voltammogram of Ru-3 in deaerated DMSO, containing 0.10 M Bu_4NPF_6 as supporting electrolyte and Ag/AgCl as a reference electrode. The ferrocene was used as internal reference and adjusting its value to zero, the exact potential was determined. Scan rates: 50 mV/s. $c = 5 \times 10^{-4}$ M, 20 °C.

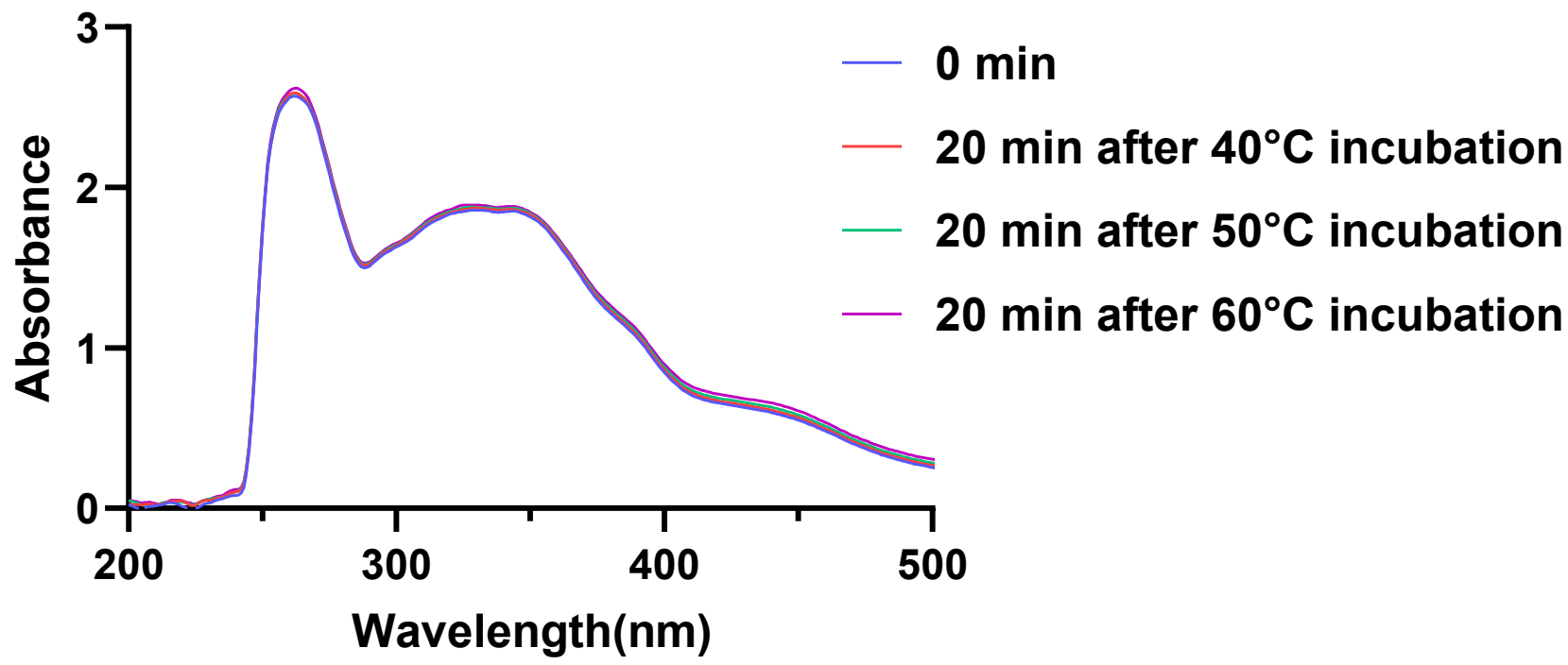

Fig. S32. UV-vis spectra of NADH under external heating conditions.

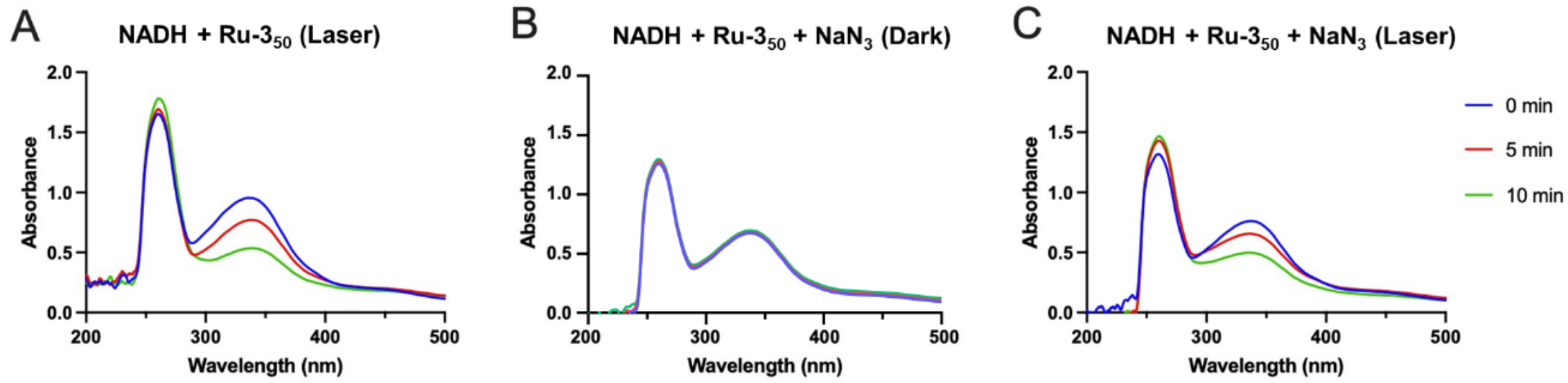

Fig. S33. Control studies with NaN_3 confirm SET-dominated NADH photooxidation under PACE conditions. (A) UV-vis spectra for **Ru-3** mediated NADH photooxidation in a H_2O/THF = 1: 1 mixture solution. (B) UV-vis spectra for **Ru-3** mediated NADH photooxidation in the presence of NaN_3 under a dark environment. (C) UV-vis spectra for **Ru-3$_{50}$** mediated NADH photooxidation in the presence of NaN_3 upon 808 + 1310 irradiation. NADH: 180 μM, **Ru-3$_{50}$**: 10 μM, the laser power densities were 0.5 W·cm^{-2} for 808 nm and 1.0 W·cm^{-2} for 1310 nm.

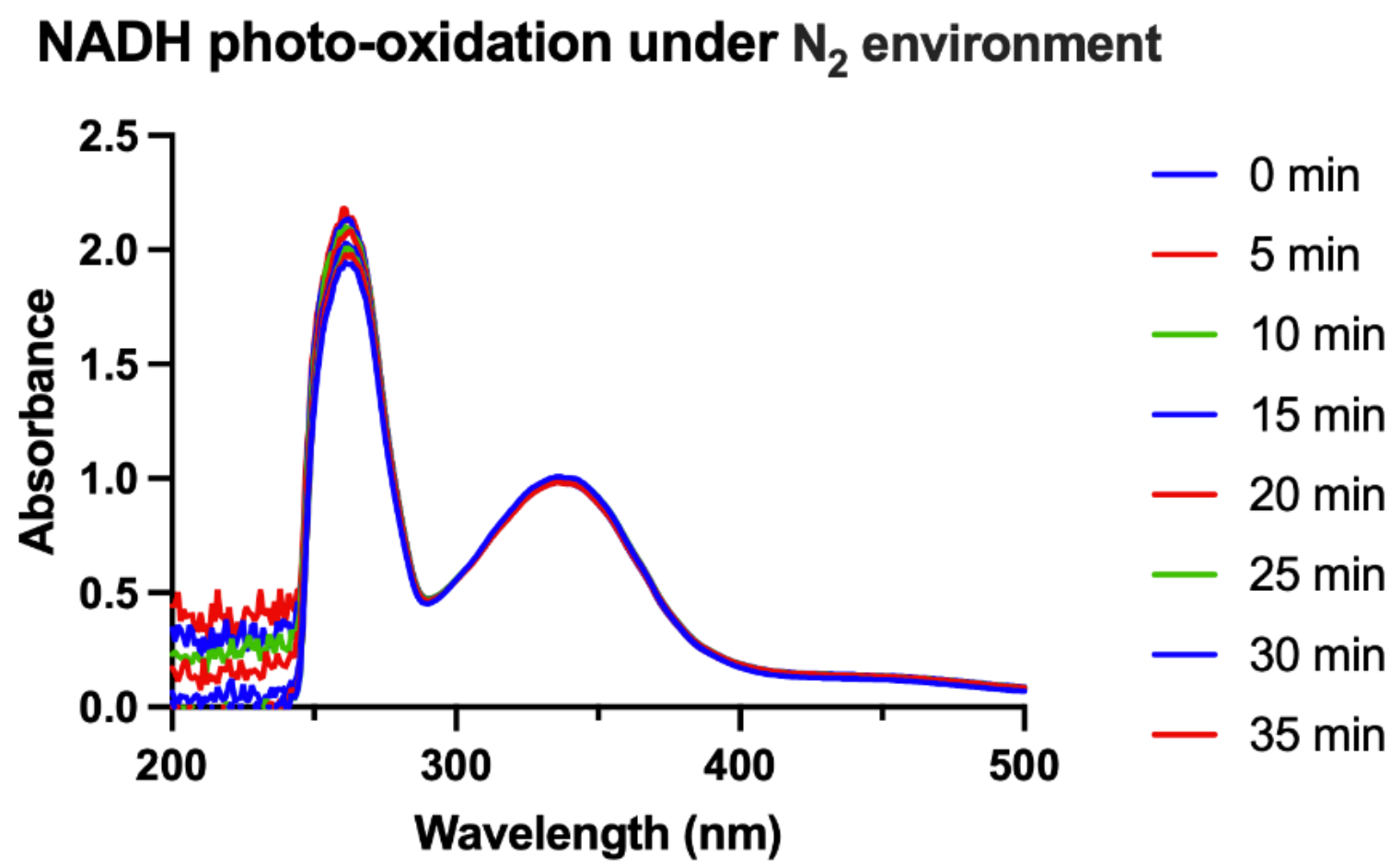

Fig. S34. Time-dependent UV-vis absorption spectra of NADH oxidation under N_2 atmosphere. The solution containing NADH (270 μM) and **Ru-3** (10 μM) in DMSO/water (1:1, v/v) was purged with N_2 for 5 min, repeated for three cycles, before irradiation with 808 nm and 1310 nm lasers. The power densities were 0.5 W cm^{-2} for 808 nm and 1.0 W cm^{-2} for 1310 nm. Spectra were recorded every 5 min from 0 to 35 min.

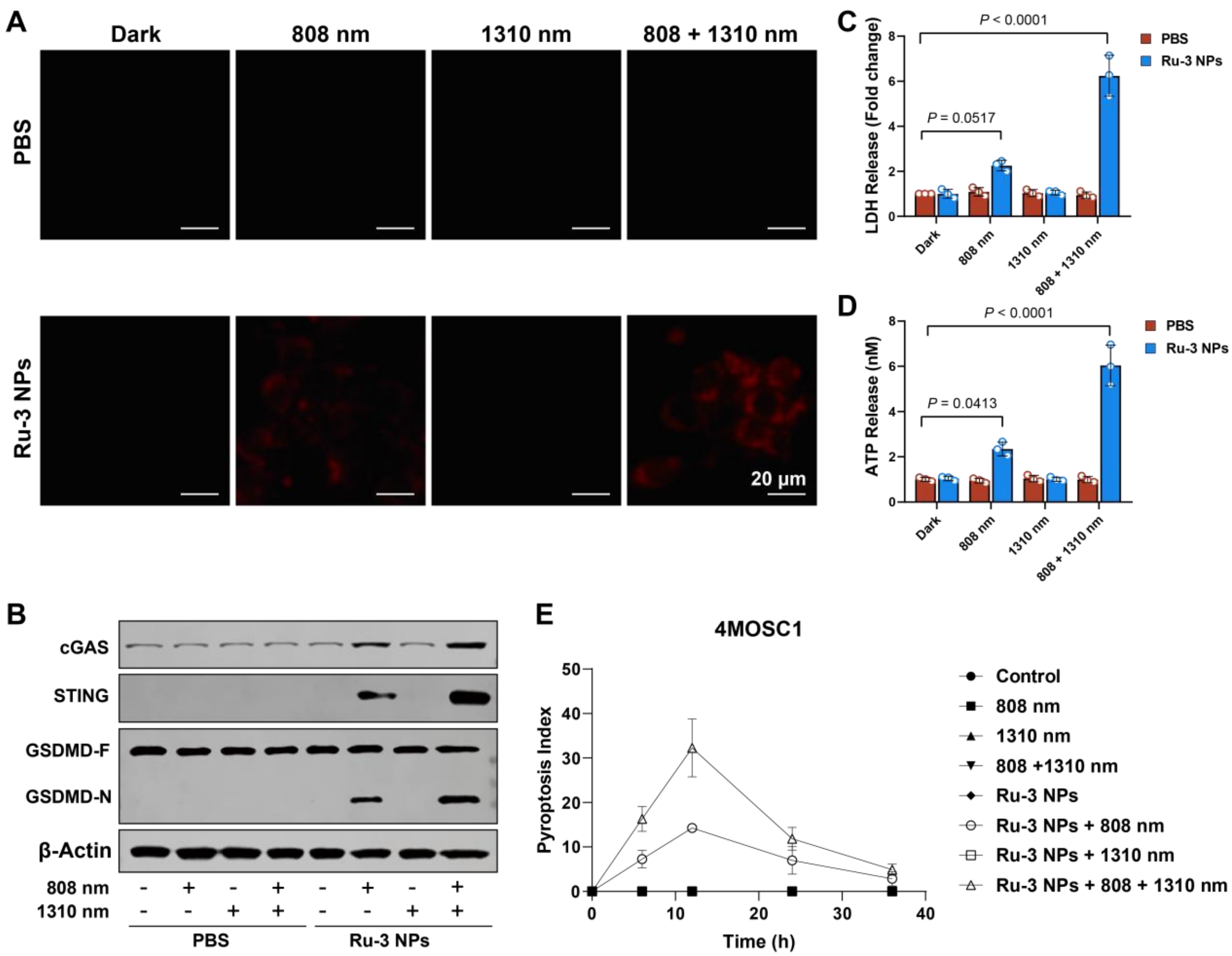

Fig. S35. PACE-induced mitochondrial stress activates cGAS–STING signaling and drives pyroptosis. (A) Representative confocal microscopy images of 4T1 cells reveal cellular ROS levels following various treatments. **(B)** Immunoblot analysis of cGAS, p-STING, GSDMD-N, and GSDMD-F in 4MOSC1 cells treated with **Ru-3 NPs** (10 μM) under diverse irradiation conditions. **(C)** LDH levels released into the culture medium of 4T1 cells after different treatments. **(D)** ATP levels released into the culture medium of 4T1 cells following various treatments. **(E)** The pyroptosis index of 4MOSC1 cells was assessed at predetermined time points (6, 12, 24, and 36 h) after different treatments.

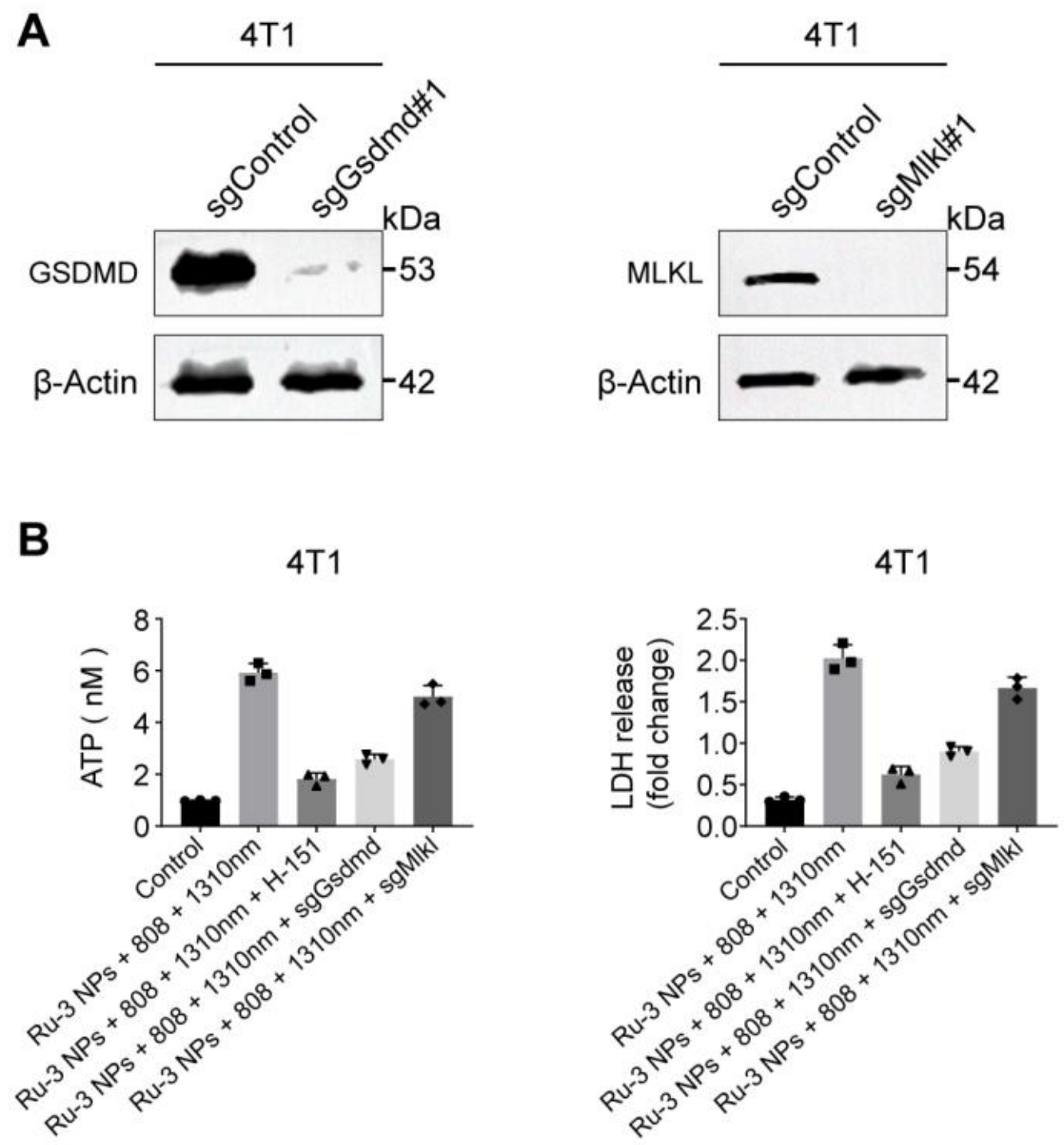

Fig. S36. The PACE system induces cGAS-STING-dependent, GSDMD-mediated pyroptosis that releases ATP and LDH. (A) The situation of 4T1 cells transfected with sgRNA (Gsdmd, Mlkl) was detected by western blotting. Data were repeated thrice independently with similar results. n = 3 independent biological replicates. (B) Detection of ATP and LDH release in 4T1 cells from different treatment groups. All data are shown as the mean ± s.e.m. n = 3 independent biological replicates. Statistical significance was calculated via one-way ANOVA with Tukey's multiple comparisons test. n.s. not significant. Data are representative of three independent experiments.

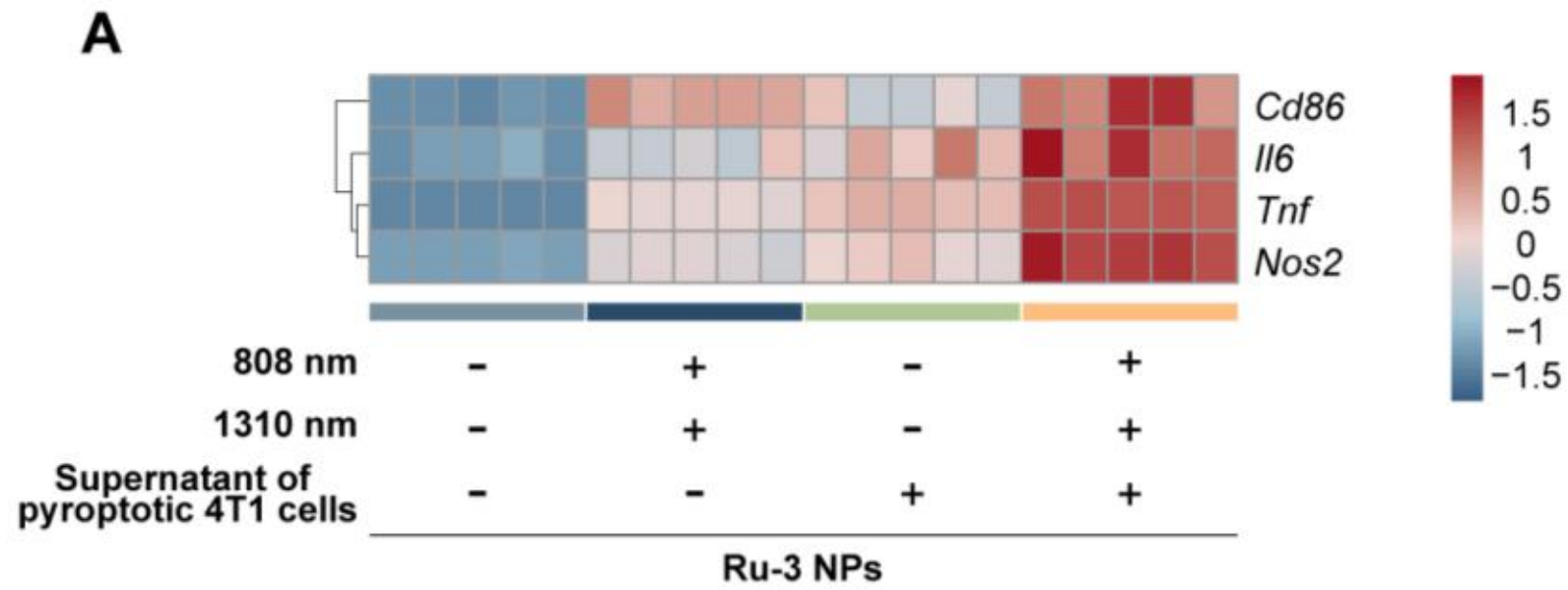

Fig. S37. Induction of tumor cell pyroptosis and macrophage polarization by PACE. (A) Heatmap showing mRNA levels of M1 macrophage markers (n = 5). Two treatment methods were used: 1) direct treatment of macrophages with 10 μM **Ru-3 NPs** combined with laser irradiation (808 nm, 0.5 W/cm^2; 1310 nm, 1.0 W/cm^2); 2) collection of the supernatant from 4T1 tumor cells that underwent pyroptosis induced by the same concentration of **Ru-3 NPs** and the same laser conditions, which was then used to treat macrophages.Normalized mRNA level is reported as mean ± s.d. For ANOVA tests with Dunnett's multiple comparisons test, *, $P < 0.05$; **, $P < 0.01$; ***, $P < 0.001$; ****, $P < 0.0001$; ns indicates non-significant.

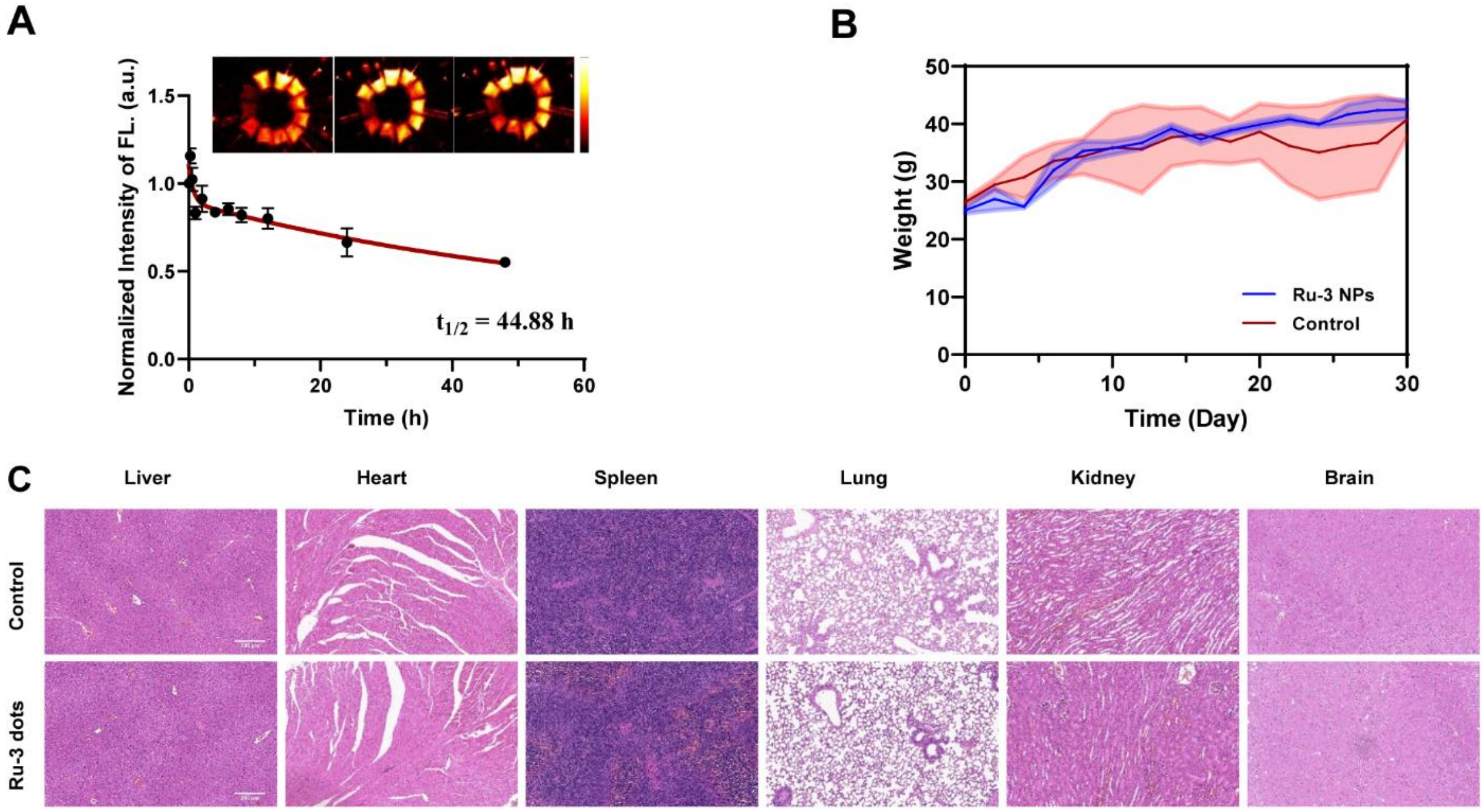

Fig. S38. Pharmacokinetics and biocompatibility of Ru-3 nanoparticles in vivo. (A) NIR-II images and corresponding fluorescence intensity of blood samples collected from mice at predetermined time points. The biological half-life of **Ru-3 NPs** was obtained from the fitting curve. **(B)** Body weight changes in mice after intravenous administration of PBS or **Ru-3 NPs**. The experiments were conducted with biologically independent replicates (n = 3). **(C)** Long-term toxicity study showcasing major organs (heart, liver, spleen, lung, kidney, and brain) with H&E staining 30 days post-intravenous injection of PBS (upper) and **Ru-3 NPs** (lower), scale bar: 200 μm. The presented results are representative of three independent experiments.

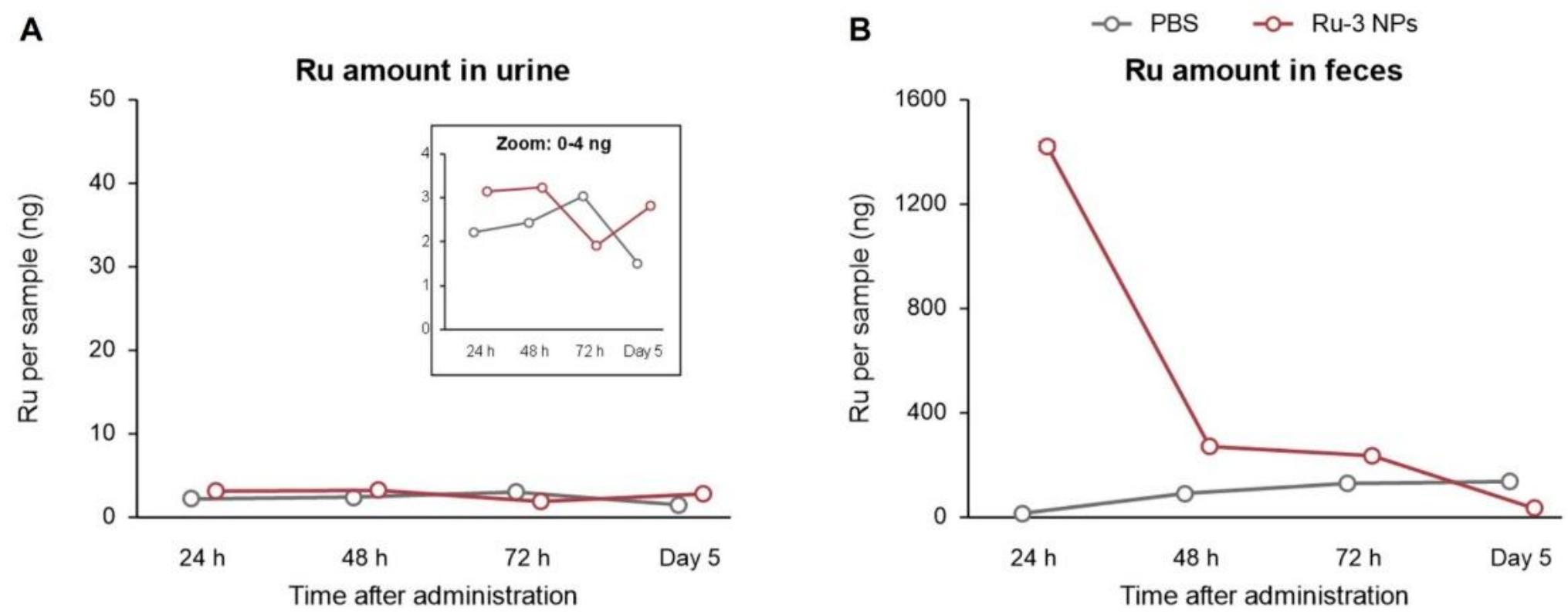

Fig. S39. Excretion profile of Ru-3-derived ruthenium after Ru-3 NP administration. Calculated Ru amount per collected urine (c) and feces (d) sample determined by ICP-MS. Urine and feces were collected at 24 h, 48 h, 72 h and day 5 after administration of **Ru-3 NPs** (45 µM, 200 µL) or PBS to BALB/c mice. To avoid Ru levels falling below the detection limit, six mice from each treatment group were housed in one metabolic cage for pooled excreta collection. ICP-MS was performed using a PE NexION 1000G system with RF power 1.6 kW, nebulizer gas flow 1.01 L min^{-1}, auxiliary gas flow 1.20 L min^{-1}, plasma gas flow 15 L min^{-1}, KED gas flow 5.9 mL min^{-1}, uptake time 45 s, stabilization time 30 s and integration time per mass 0.90 s. Data in c and d are mean +/- s.d. of three technical ICP-MS readings.

c

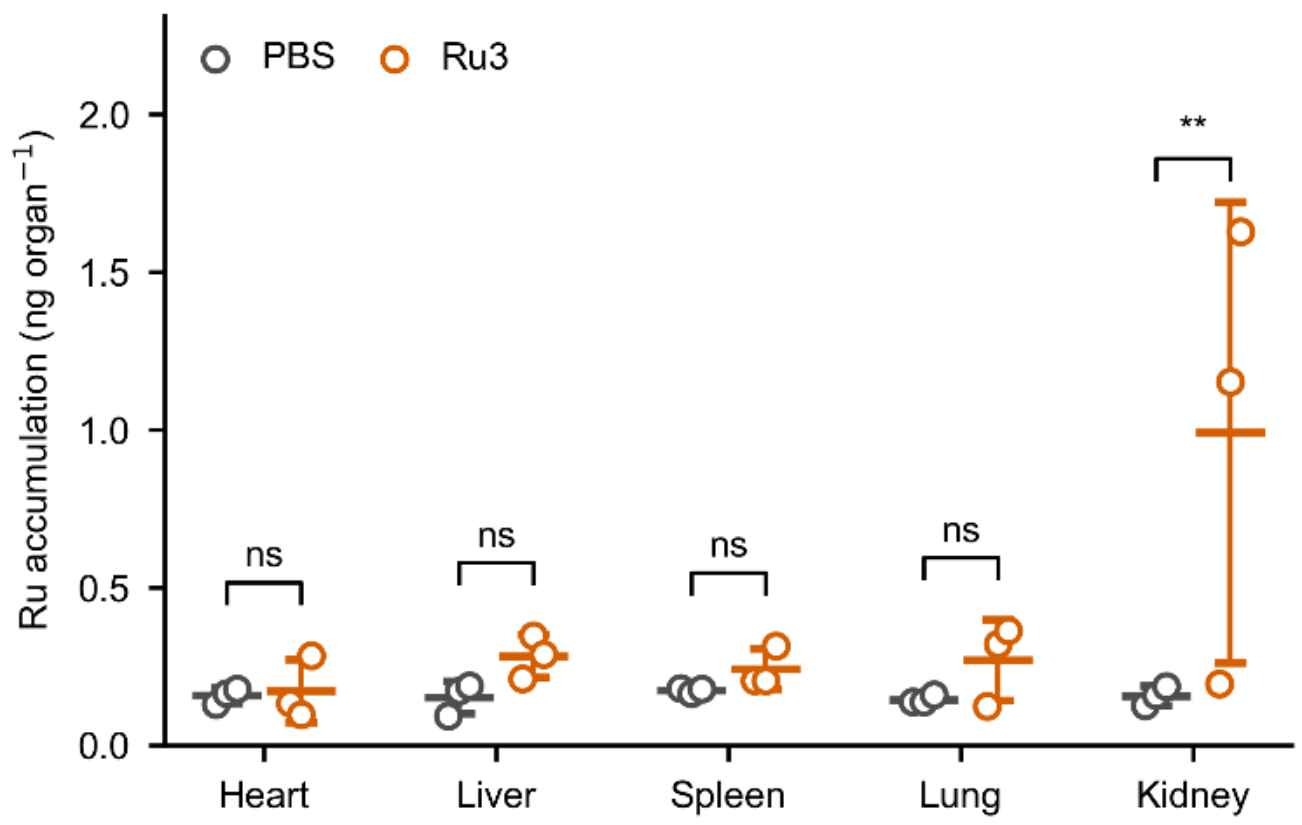

Fig. S40. Long-term organ accumulation of ruthenium 50 days after Ru-3 NPs administration. 50 days after PBS or **Ru-3** (45 μM, 200 μL) administration, mice were sacrifices, and heart, liver, spleen, lung and kidney were excised and weighed. Organs were microwave digested, and Ru was quantified by ICP-MS using a PE NexION 1000G system with the following operating conditions: RF power, 1.6 kW; nebulizer gas flow, 1.01 L min^{-1}; auxiliary gas flow, 1.20 L min^{-1}; plasma gas flow, 15 L min^{-1}; KED gas flow, 5.9 mL min^{-1}; uptake time, 45 s; stabilization time, 30 s; and integration time per mass, 0.90 s. Organ Ru accumulation was calculated as the Ru concentration in wet tissue multiplied by the corresponding whole-organ wet weight. Each point represents one biologically independent mouse after averaging three analytical replicate readings for each organ. Statistical analysis was performed using ordinary two-way ANOVA followed by Dunnett's multiple-comparisons test comparing Ru-3 NPs with PBS within each organ. *P < 0.05; **P < 0.01; ***P < 0.001; ****P < 0.0001; ns, not significant.

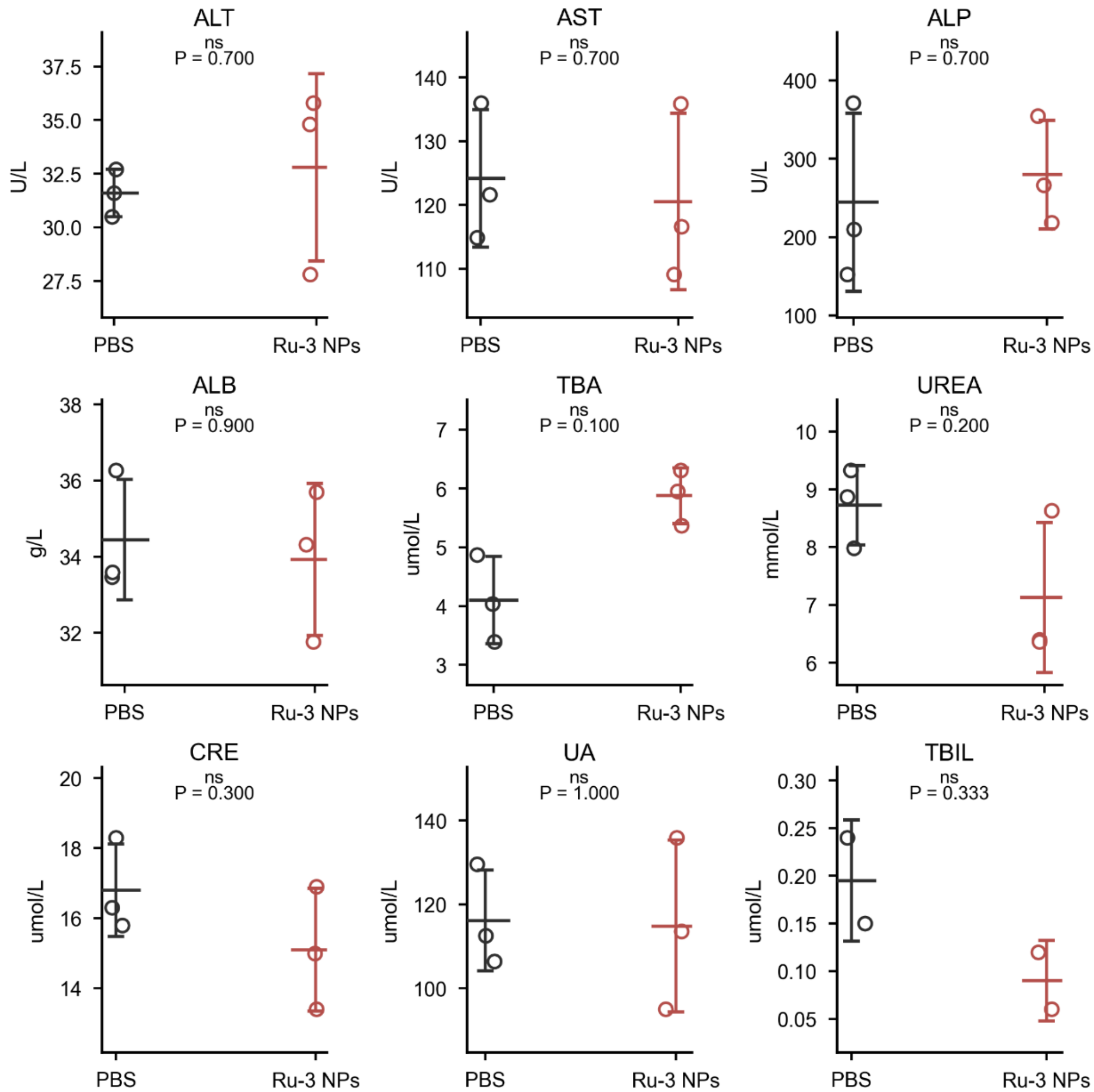

Fig. S41. Serum chemistry profile of mice treated with Ru-3 NPs. BLAB/c mice were treated with PBS or **Ru-3 NPs** (45 µM, 200 µL) for single i.v. injection. Serum was collected 50 days after treatment and analyzed for hepatic, renal and metabolic chemistry markers. D-BIL levels were below the detection limit and are therefore not shown here. permutation P values and significance labels are shown; ns, $P \geqslant 0.05$. n = 3 per group after exclusion except TBIL, where missing values reduced the available n. No statistically significant treatment-associated alteration was detected for ALT, AST, ALP, ALB, TBA, UREA, CRE or UA under the analyzed conditions.

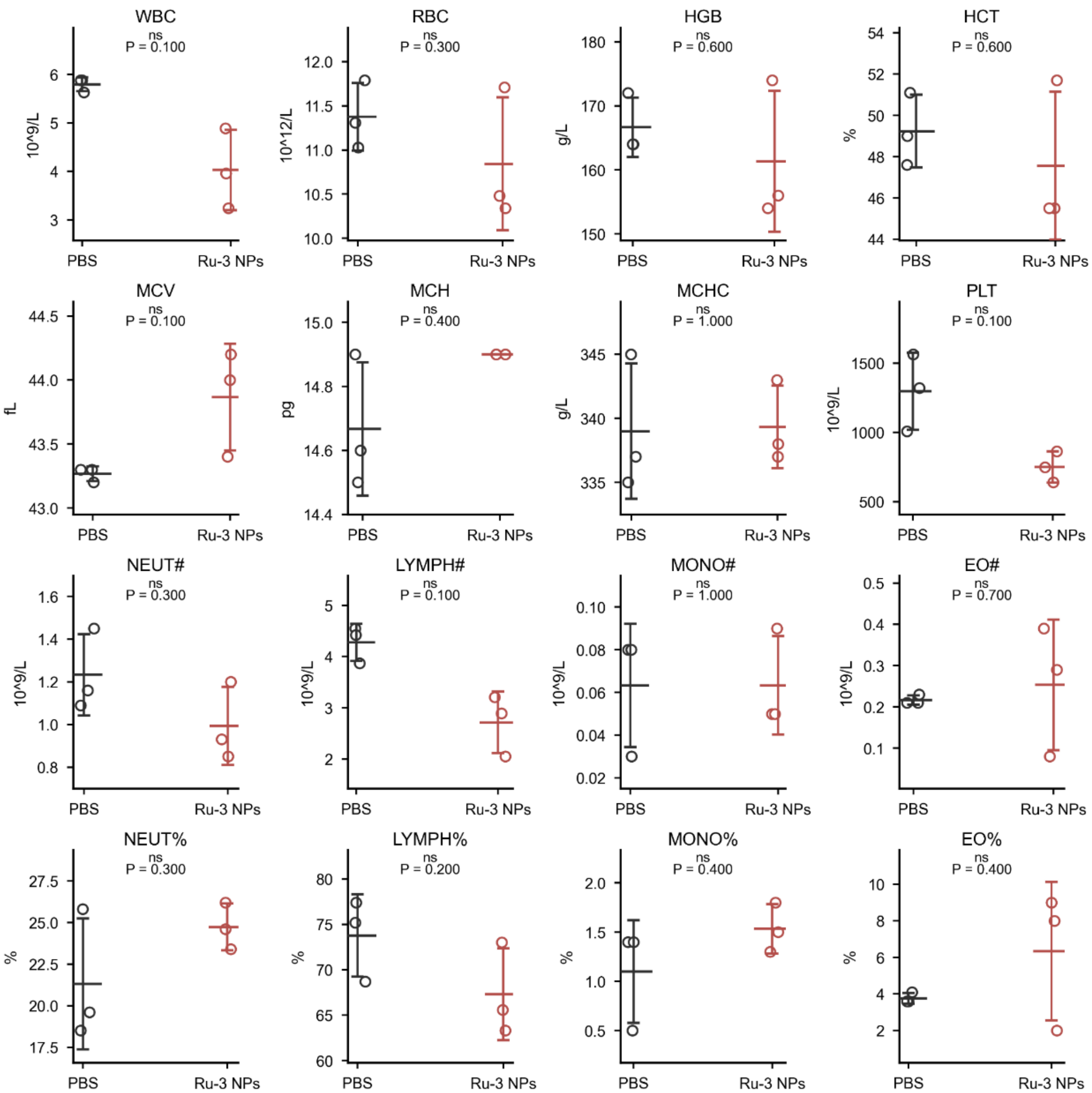

Fig. S42. Complete blood count and leukocyte differential after Ru-3 NPs treatment. Peripheral whole blood from PBS- and **Ru-3 NPs**-treated mice was analyzed 50 days after treatment. Exact two-sided permutation P values and significance labels are shown; ns, P >= 0.05. Across erythrocyte, platelet and leukocyte indices, **Ru-3 NP** treatment did not produce a statistically significant deviation from PBS controls after exclusion.

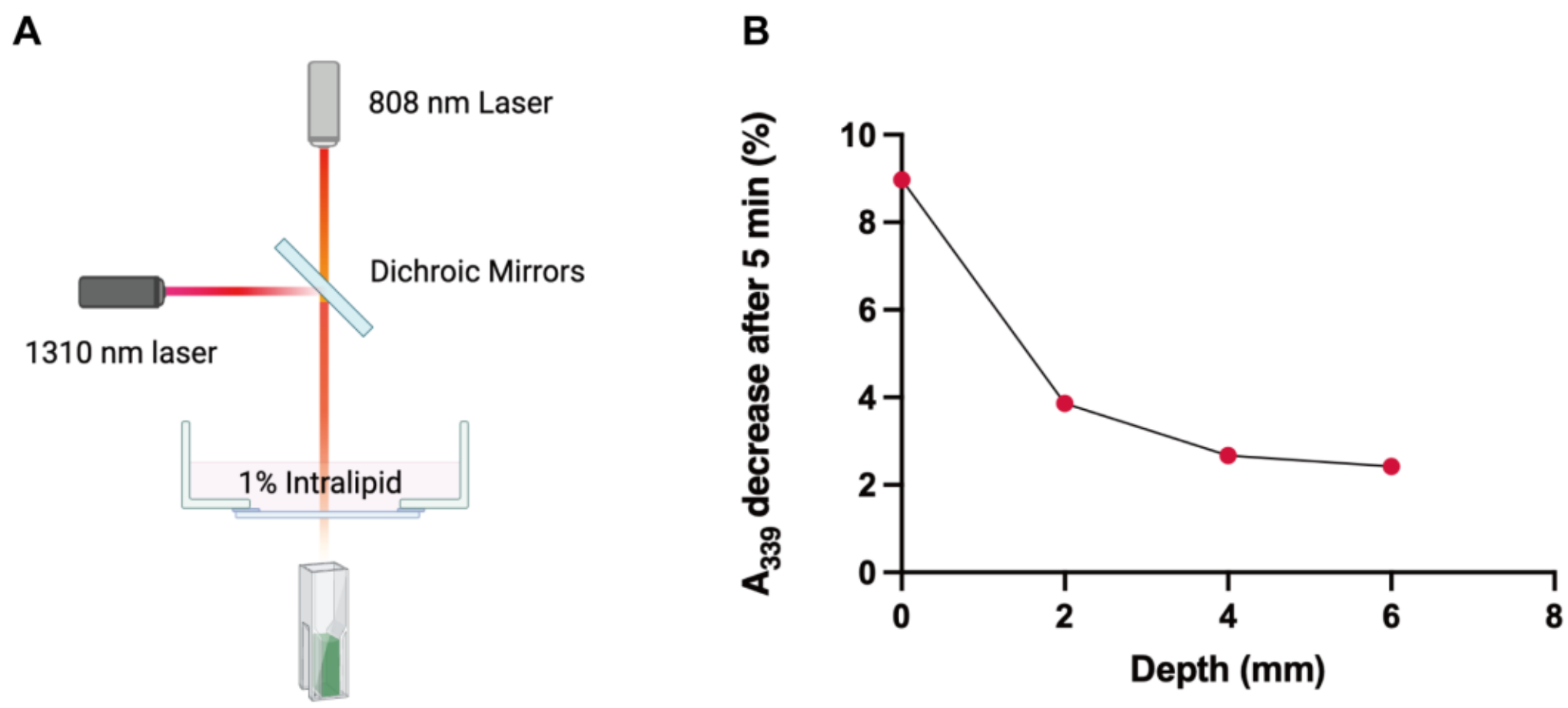

Fig. S43. Depth-dependent NADH photooxidation after shielding with 1% intralipid. (A) Schematic illustration of the experimental setup. NADH solution (270 μM) was irradiated simultaneously with 808 nm and 1310 nm lasers at power densities of 0.5 W cm^{-2} and 1.0 W cm^{-2}, respectively. 1% intralipid layer with nominal thicknesses of 0, 2, 4, or 6 mm was placed between the light source and the sample to model tissue-like optical scattering. Created in BioRender. R, YU. (2026) https://BioRender.com/9f63dex. (B) Relative absorbance at 339 nm measured over 5 min under combined 808 and 1310 nm irradiation in the presence of Ru-3 in a DMSO/H_2O (1:1, v/v) mixture.

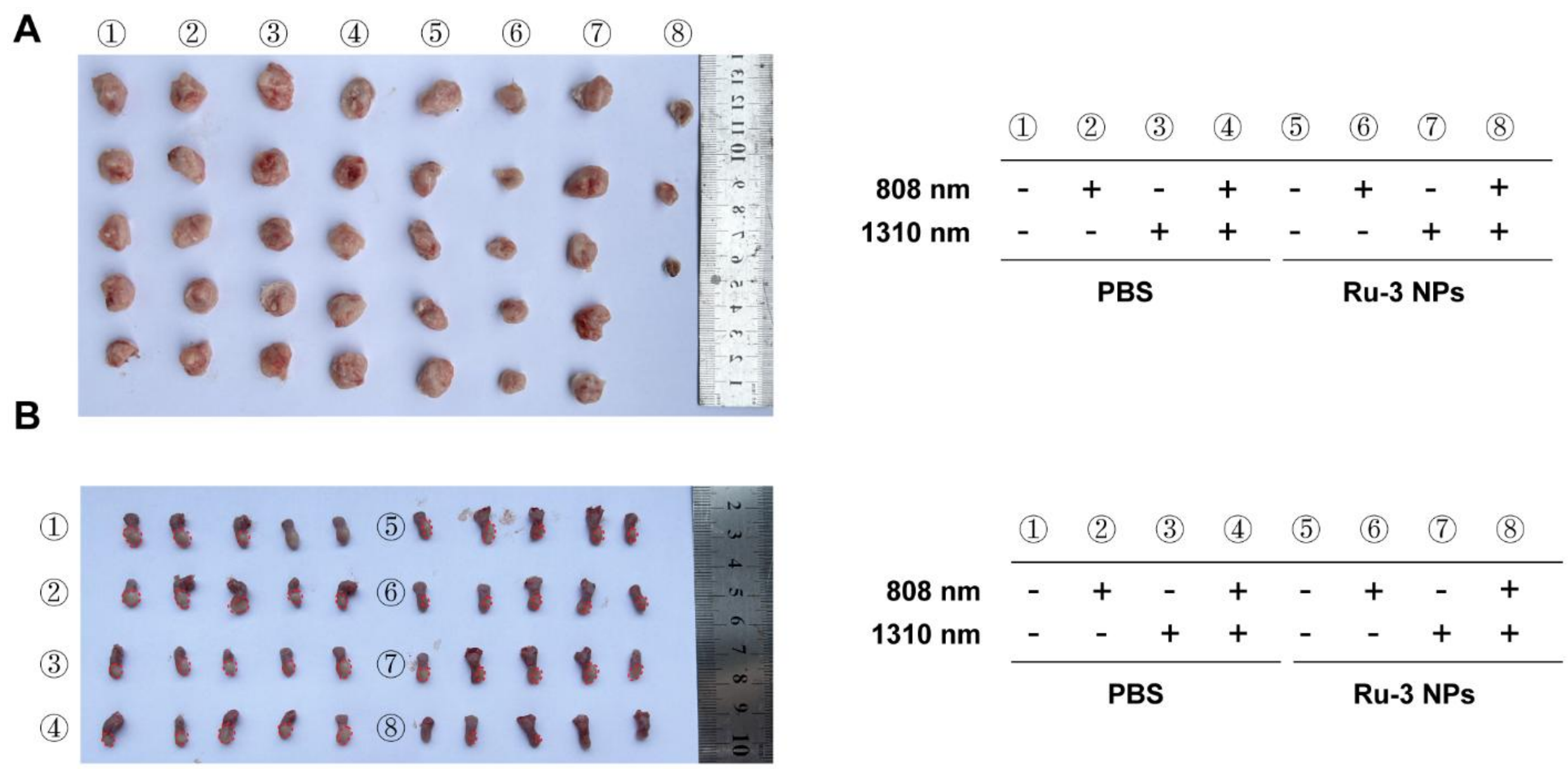

Fig. S44. PACE-enhanced antitumor efficacy of Ru-3 nanoparticles in vivo. **(A)** Optical images of 4T1 tumors and **(B)** 4MOSC1 tumors harvested at the end point of the treatment. Group 1 to 4 represent for control, 808 nm, 1310 nm, 808 + 1310 nm treatment with PBS administration. Group 5 to 8 represent for control, 808 nm, 1310 nm, 808 + 1310 nm treatment with **Ru-3 NPs** administration. The power intensity of 808 and 1310 nm laser was 0.5 W·cm^{-2} and 1.0 W·cm^{-2}, respectively.

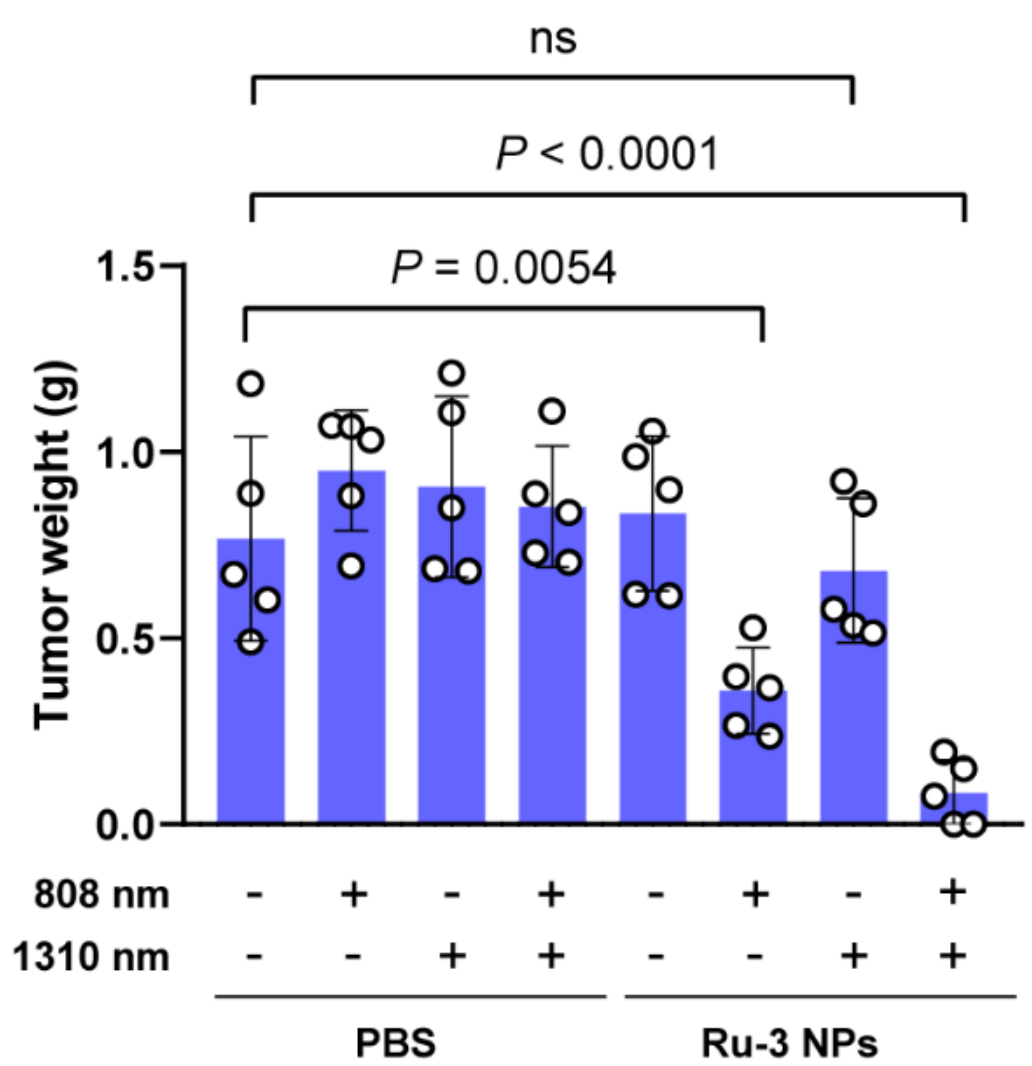

Fig. S45. Weight of the 4T1 tumors collected at the end point of the treatments.

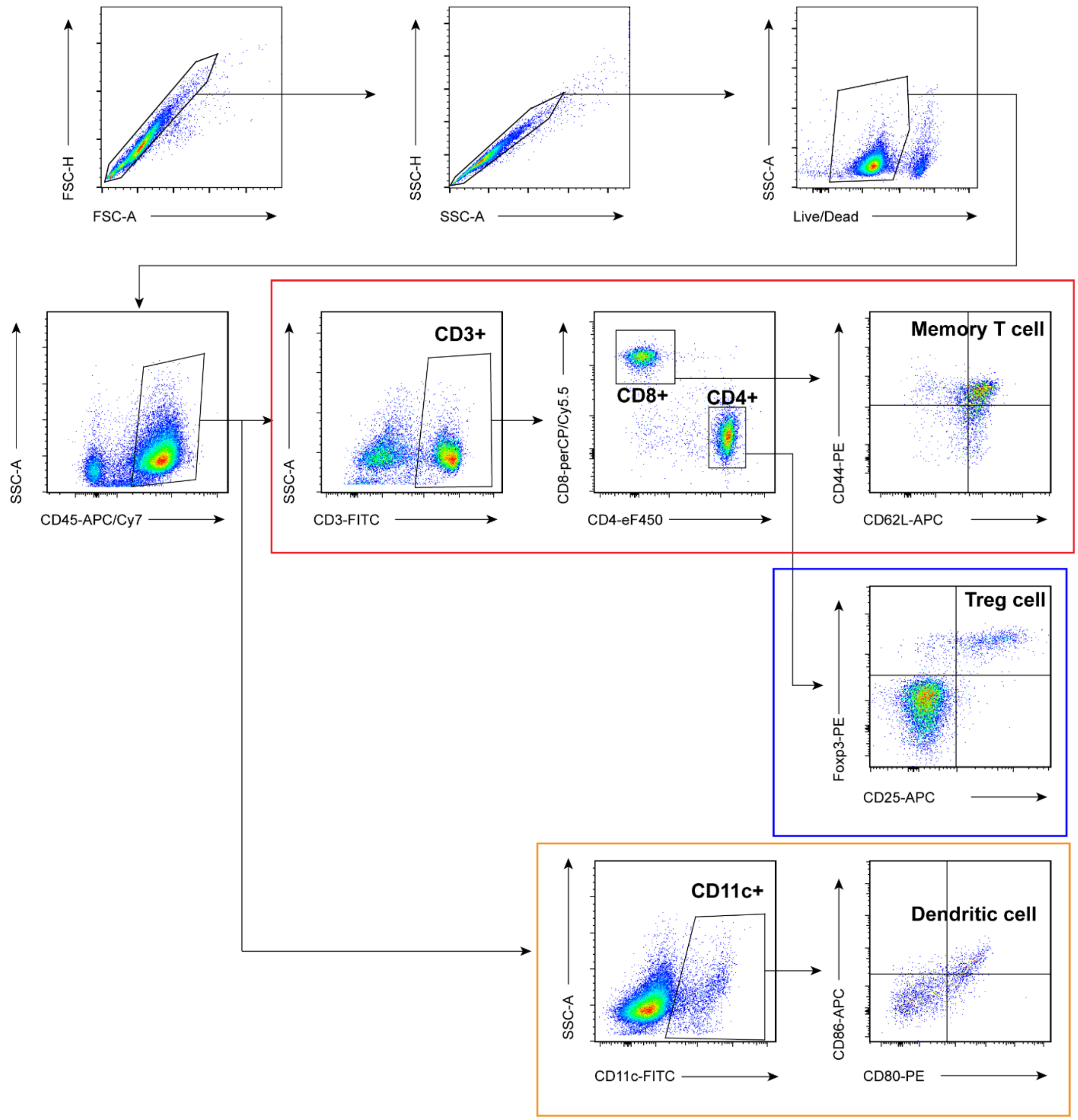

Fig. S46. Gating strategy for flow cytometry analysis of the immune responses. The figure depicts the gating strategies for $CD8^+$ memory cells, Treg cells, and Dendritic cells present in draining lymph node of tumor bearing mice.

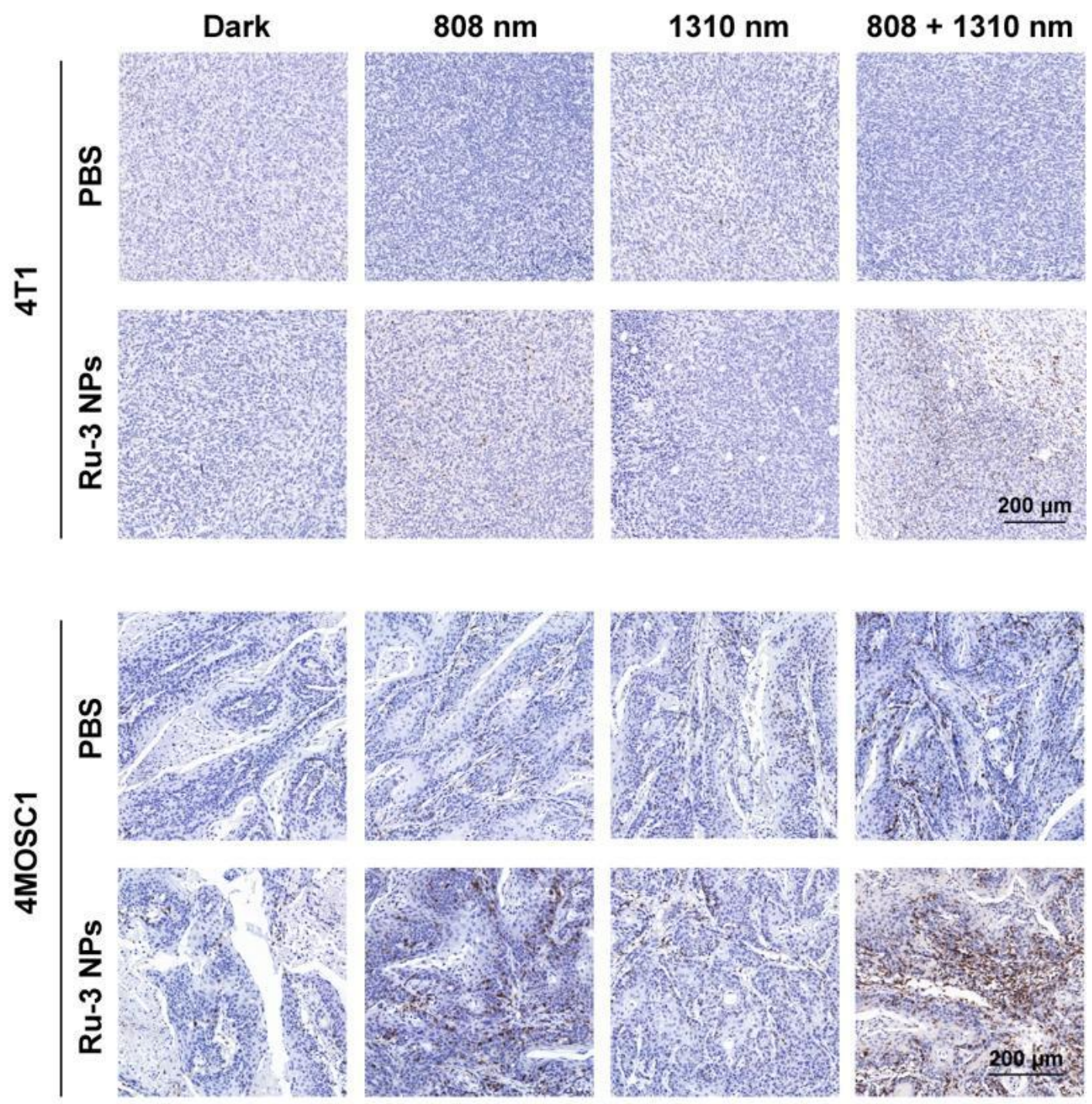

Fig. S47. PACE-enhanced CD8$^+$ T cell infiltration in tumor tissues. Representative IHC images of 4T1 and 4MOSC1 tumor tissues collected at the end point of treatment measuring CD8 level after various treatments. Scale bars, 200 μm.

Table S1. Slope indicating the degradation rate of the DPBF in DMSO/Water solution containing either ICG or H7.

	ICG	H7							
		**0%**	**20%**	**40%**	**50%**	**60%**	**70%**	**80%**	**95%**
**Slope**	-0.016	0.001	-0.001	-0.004	-0.005	-0.006	-0.006	-0.004	-0.002
$\frac{k_{PS} \times F_{ICG}}{k_{ICG} \times F_{PS}}$	/	0	0	0.224	0.273	0.487	0.428	0.541	0.080

The percentage indicates the water fraction. k is the slope obtained by linear fitting of the decreased absorption intensity of DPBF, and F is the absorption correction factor, which is given by F = 1-10^{OD} (OD at the irradiation wavelength).

Table S2. Slope indicating the degradation rate of the DPBF in DMSO/Water solution containing either ICG or Ru-1.

	ICG	Ru-1						
		0%	20%	40%	50%	60%	70%	95%
**Slope**	-0.016	0	0	0	-0.004	-0.004	-0.003	-0.001
$\frac{k_{PS} \times F_{ICG}}{k_{ICG} \times F_{PS}}$	/	0	0	0	0.281	0.281	0.211	0.070

The percentage indicates the water fraction. k is the slope obtained by linear fitting of the decreased absorption intensity of DPBF, and F is the absorption correction factor, which is given by F = 1-10^{OD} (OD at the irradiation wavelength).

Table S3. Slope obtained from the degradation curve of the DPBF in DMSO/Water solution containing either ICG or Ru-3.

	ICG	Ru-3							
		0%	20%	40%	50%	60%	70%	80%	95%
**Slope**	-0.016	0	-0.004	-0.036	-0.060	-0.025	-0.034	-0.026	-0.015
$\frac{k_{PS} \times F_{ICG}}{k_{ICG} \times F_{PS}}$	/	0	0.311	2.667	5.275	2.091	2.425	1.876	0.889

The percentage indicates the water fraction. k is the slope obtained by linear fitting of the decreased absorption intensity of DPBF, and F is the absorption correction factor, which is given by F = 1-10^{OD} (OD at the irradiation wavelength).

Table S4. The cellular Ru concentration evaluated by ICP-MS.

Fraction	V (mL)	V_0 (mL)	Element	C_o (μg/L)	f	C_1 (μg/L)
Mitochondria	1	10	Ru	0.058	10	0.58
The rest	0.2	10	Ru	0.117	50	5.85

V: sample volume; V_0: the volume after dilution; C_0: the elemental concentration in the test solution; f: dilution factor; C_1: the elemental concentration in the sample solution.

Table S5. Primers used in this work

Primer	Forward	Reverse
Gapdh	TGTGTCCGTCGTGGATCTGA	TTGCTGTTGAAGTCGCAGGAG
Il6	TAGTCCTTCCTACCCCAATTTCC	TTGGTCCTTAGCCACTCCTTC
Cd86	TCAATGGGACTGCATATCTGCC	GCCAAAATACTACCAGCTCACT
Nos2	GGAGTGACGGCAAACATGACT	TCGATGCACAACTGGGTGAAC
Tnf	TATGGCCCAGACCCTCACA	GGAGTAGACAAGGTACAACCCATC
Tgfb	TGATACGCCTGAGTGGCTGTCT	CACAAGAGCAGTGAGCGCTGAA
Arg1	CTCCAAGCCAAAGTCCTTAGAG	GGAGCTGTCATTAGGGACATCA
Cd206	CTCTGTTCAGCTATTGGACGC	TGGCACTCCCAAACATAATTTGA
